# The Roles of the Membrane-Anchored Glycoprotein RECK in Animal Development, Tumor Suppression, and Beyond

**DOI:** 10.3390/life16010104

**Published:** 2026-01-11

**Authors:** Makoto Noda, David Alexander, Tomoko Matsuzaki

**Affiliations:** 1 Department of Molecular Oncology, Kyoto University Graduate School of Medicine, Yoshida-Konoe-cho, Sakyo-ku, Kyoto 606-8501, Japan; matsuzaki.tomoko.7w@kyoto-u.ac.jp; 2 Department of Molecular Toxicology, Nagoya City University Graduate School of Medical Sciences, 1 Kawasumi, Mizuho-cho, Mizuho-ku, Nagoya 467-8601, Japan; dalexand@phar.nagoya-cu.ac.jp; 3 Institute of Laboratory Animals, Kyoto University Graduate School of Medicine, Yoshida-Konoe-cho, Sakyo-ku, Kyoto 606-8501, Japan

**Keywords:** RECK, cancer, metastasis, development, angiogenesis, ECM, MMP, WNT7

## Abstract

*RECK* was first reported as a transformation suppressor gene in 1998 and gradually gained attention as evidence indicating its reduced expression in a wide variety of human cancers accumulated. *RECK* encodes a membrane-anchored glycoprotein exhibiting protease inhibitor activity against matrix metalloproteases. Restored expression of RECK in cancer xenograft models suggests it suppresses tumor growth and/or metastasis. RECK was also found to be essential for mammalian embryogenesis, especially in the maintenance of tissue integrity as well as the development of neural and vascular systems. Due to its functional versatility during animal development, we only recently began to obtain formal experimental evidence that *RECK* is a bona fide tumor suppressor. In the meantime, mechanisms by which RECK expression is reduced in cancer cells have been explored. Various stimuli that alter RECK expression have also been described. Furthermore, recent findings in the clinic as well as in animal studies indicate the involvement of RECK in disorders other than cancer. The aim of this article is to summarize our current knowledge of RECK and assist future efforts to understand its nature and functions and to develop useful applications.

## 1. Introduction

In the late 1980s, evidence for tumor suppressor genes was still circumstantial [1,2]. Inspired by the groundbreaking approach by Shih and Weinberg [3] to detect and isolate cellular oncogenes by DNA transfection, Noda et al. [4] made the first attempt to isolate tumor suppressor genes by transfection of a cDNA-expression library, made by Okayama and Berg [5], into a transformed mouse fibroblast cell line. The phenotype used for screening was a morphological reversion (or “flat reversion”, which refers to increased adhesion to culture dishes) of transfected cells. The first gene isolated by this method was a novel *RAS*-related gene termed *Krev-1* (now known as *RAP1A*) [6]. *RECK*, the subject of this review, was isolated using an updated version of the expression vector used to transfect target cells. In the first study describing *RECK* [7], Takahashi et al. reported that RECK inhibited matrix metalloproteinase-9 (MMP9) and suppressed tumor metastasis in mouse xenograft models. They also reported that the expression of the endogenous *RECK* gene was suppressed after cell transformation [7,8]. Subsequent studies with clinical samples indicated that RECK expression tends to be lower in tumors with poorer prognoses [9,10,11,12]. In 2001, Oh et al. [13] described the phenotype of *Reck*-deficient mice, demonstrating its essential roles in maintaining tissue integrity and, in particular, supporting vascular and neural development. Although these earlier findings, cited in several reviews [14,15,16,17,18], suggested the involvement of RECK in tumor suppression as well as mammalian development, it was clear that many more studies had to be performed before we could understand how and to what extent RECK contributes to these events. Studies in the last quarter of a century have yielded a substantial amount of knowledge regarding RECK, providing at least partial answers to this question. Although some specific aspects of these studies have been reviewed [19,20,21,22,23,24,25,26,27], since our knowledge in this field is steadily accumulating and since the descriptions of RECK in the literature are complex, we believe it worthwhile to comprehensively overview (in a topically categorized way) what we have learned of this interesting protein to date in order to gain a fresh perspective on the functions of RECK.

## 2. Gene Structure and Polymorphisms

**Figure 1:**
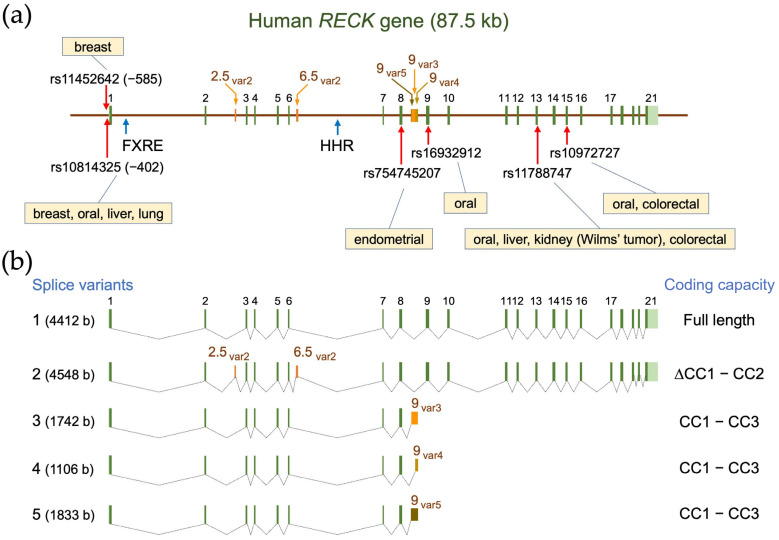
Human *RECK* gene and its transcripts. (**a**) Schematic representation of the human *RECK* gene. Coding exons are represented by green bars (with exon numbers above them) and untranslated sequences by the pale green box. Arrows in blue indicate the positions of a farnesoid X receptor binding element (FXRE) in intron 1 (see Section 8.3.5) and a hammerhead ribozyme (HHR) in intron 6. Arrows in red indicate the positions of SNPs and arrows in orange, ocher, or brown indicate the positions of alternative exons. Tumor types associated with each SNP are listed in the connected box (see Table S1 for references). (**b**) Structures of the authentic *RECK* mRNA (Variant 1) and four splice variants (Variants 2–5). Coding potential of each mRNA species is denoted on the right side. CC refers to cysteine knot domain and is described in Section 3.1. Variant 2 encodes a protein lacking the CC1 and CC2 domains, whereas variants 3 to 5 encode a protein consisting of the first three CC domains (CC1–CC3), followed by a unique amino acid sequence.

The human *RECK* gene, mapped to chromosome 9p13-p12, consists of 21 exons (Figure 1) and spans over 87.5 kb [28]. An evolutionary conserved hammerhead ribozyme (HHR) sequence is found in intron 6 [29], although its biological significance remains elusive. Eisenberg et al. (2002) [28] described 13 single-nucleotide polymorphisms (SNPs) within or around the major twenty one *RECK* exons. Table S1 lists the six SNPs, two SNPs in the promoter region of *RECK*, and four SNPs in the coding region, associated with cancer (see Figure 1 for their positions). Lei et al. (2007) [30] examined two SNPs, rs11452642 and rs10814325, in the proximal upstream region of *RECK*, together with SNPs in six other genes, in breast cancer patients. They found that patients heterozygous (T/C) at the rs10814325 site exhibited higher survival rates than homozygote (T/T) patients, suggesting that the C allele is protective against cancer. In contrast, the study by Chung et al. (2011) [31] suggested that the T allele at this site is protective against oral cancer among betel-quid-chewers and smokers. Subsequent data on liver cancer (hepatocellular carcinoma; HCC) [32,33] and lung cancer (non-small cell lung cancer; NSCLC) suggested a protective role for the T allele. Another study with a relatively small number (*n* = 30) of patients with HCC associated with hepatitis C virus (HCV), however, indicated no effect of this SNP on cancer formation [34]. Reasons for the apparent discrepancies among these studies and whether the two upstream SNPs affect *RECK* expression remain to be clarified.

Among coding-region SNPs, four have been associated with carcinogenesis. Shivakumar et al. (2019) [35] performed a genome-wide association study (GWAS) for Lynch syndrome (LS) patients: LS patients carry mutations in mismatch-repair genes. They found a prominent association of *RECK* gene variations with endometrial cancer. They identified eight SNPs/variations in *RECK*, including a G to C SNP at rs754745207 in exon 8: they noted that the same mutation in endometrial cancer was also listed in the COSMIC database (https://cancer.sanger.ac.uk/cosmic/login, accessed on 29 December 2025).

In the aforementioned study on oral cancer, Chung et al. (2011) [31] suggested protective roles of three coding-region SNPs against lifestyle-related oral cancer: namely, G at rs16932912 (exon 9), A at rs11788747 (exon 13), and T at rs10972727 (exon 15). For the exon-9 SNP, the protective role of G was supported by another study by Zhang et al. on ameloblastoma [36]. For the exon-13 SNP, the protective role of A was supported by studies on liver cancer [32,37] and Wilms’ tumor [38] (a rare kidney disease in children), although opposite results (protective role for the G allele) was obtained in studies on liver cancer [39] and on colorectal cancer [40]. For the exon-15 SNP, the protective role of the T allele, initially described by Chung et al. [31], was not supported by the study on colorectal cancer [40]. As seen in Table S1, some groups found that the major SNP alleles were protective while other groups found that the minor alleles were protective. The contrasting findings indicate that more studies are needed to clarify which types of cancer and to what extent these coding region SNPs contribute to or protect against cancer formation.

How could these coding-region SNPs affect the RECK protein? The exon-8 SNP (rs754745207) induces a substitution (from alanine to proline) at residue 168, which resides in the L3 loop of the CC3 domain. This substitution might reduce the flexibility of the loop and affect the conformation of alpha-helices in CC3 (see Section 3.3), although consequences of this substitution remain to be experimentally elucidated. The exon-9 SNP (rs16932912) induces a substitution (valine to isoleucine) at residue 275, which resides between the CC4 and CC5 domains. In this case, Zhang et al. [36] reported that the level of RECK protein was reduced in ameloblastoma tissues carrying the A allele at this site. How this substitution leads to reduced protein level is an interesting question that needs to be addressed.

SNPs in exon 13 (rs11788747) and exon 15 (rs10972727) are both synonymous variations (no changes in amino acid sequence). Recent studies indicate that synonymous mutations are not necessarily neutral and may affect the level of mRNA or protein through multiple mechanisms [41]. Thus, examining the effects of these SNPs on the levels of RECK mRNA and protein should be an important next step.

Notably, while a *RECK* ortholog is absent in the genome of the nematode (*Caenorhabditis elegans*), *RECK* is present and well-conserved from the fruit fly (*Drosophila melanogaster*) to mammals [42,43,44]. It is remarkable that this gene is found as a single gene per haploid genome in many organisms, including zebrafish [45] which is known to have the most genes with two copies per haploid genome due to teleost whole genome duplication. Hence, *RECK* seems to be dispensable for the life of nematodes but essential for survival of insects and higher organisms, and once acquired, this gene seems to face strong evolutionary pressure to keep it single, providing interesting clues to the function and regulation of this gene.

Five splicing variants (Variant 1–5; Figure 1b) are listed in the NCBI database. Variant 1 (also termed “canonical RECK” or “long RECK”; NM_021111) corresponds to the authentic (best-characterized) species of *RECK* mRNA. Variant 2 (NM_001316345) contains two extra exons (exons 2.5_vas2_ and 6.5_var2_ in Figure 1a) in introns 2 and 6, respectively, and has the potential to encode a protein lacking the CC1 and CC2 domains (see Section 3.1 for a discussion of protein domains). However, the initiation codon in this mRNA is not in a good context of the Kozak consensus sequence, and consequently, whether such a protein is actually produced in vivo remains to be confirmed. Variants 3 to 5 (NM_001316346, NM_001316347, NM_001316348) are similar in that they all result from alternative splicing from exon 8 into one of three ancillary exons (9_var3_, 9_var4_, and 9_var5_ in Figure 1a) present in intron 8. These variants share a common 3′-end (i.e., poly-A site) and encode the first three CC domains (212 amino acid residues) followed by COOH-terminal peptides of different amino acid sequences and lengths (36, 8, and 13 amino acid residues, respectively). Two variants termed “RECK-B” and “RECK-I” by Trombetta-Lima et al. [46] probably correspond to Variants 3 and 5, respectively. Likewise, the isoform termed “short RECK” in some reports corresponds to Variant 5. The relative abundance of the non-full-length species (i.e., Variants 2–5) may deviate in certain diseases [47,48] and may affect cellular behavior (see Section 6.2.1).

## 3. Protein Structure and Properties

### 3.1. Primary Structure, Predicted Domains, and Molecular Shape

**Figure 2:**
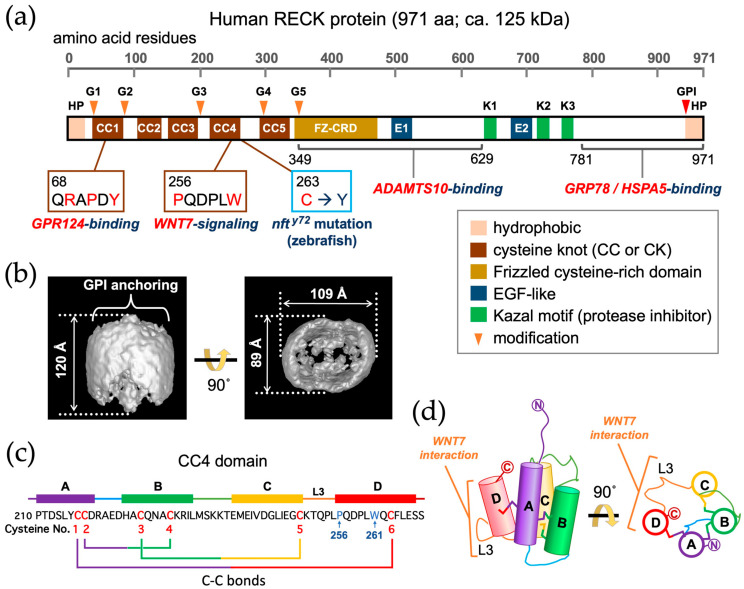
RECK protein. (**a**) Domain organization of the human RECK protein. The top bar and the color legend in the gray box below indicate the positions of motifs and domains predicted by the amino acid sequence. Regions (brackets) and residues (brown boxes) involved in protein interactions and a site of mutation found in a zebrafish mutant (blue box) are indicated under the bar. Abbreviations: HP, hydrophobic region; CC, cysteine knot domain; FZ-CADR, frizzled cysteine-rich domain; E, epidermal growth factor (EGF)-like motif; K, Kazal motif; G, N-glycosylation site. (**b**) Shape of the RECK dimer deduced by transmission electron microscopy and image analysis. (**c**) Amino acid sequence of the CC4 domain. Top diagram indicates the positions of four alpha-helices (A–D). Three pairs of cysteine residues forming CC-bonds are indicated below the sequence. (**d**) A cartoon roughly indicating the relative positions of four alpha-helices and three CC-bonds based on the 3D-structure determined by Chang et al. [44]. Colors of the helices (cylinders) and the connecting CC-bonds (hooks) match those in panel (**c**). L3 indicates L3 loop.

The human RECK protein consists of 971 amino acid residues that are typically detected as a broad band of 110–125 kilodaltons (kDa) in immunoblot assays under reducing conditions [7,49]. It is rich in cysteine (9%) and contains hydrophobic regions at both the NH_2_- and COOH-terminal ends (HP in Figure 2a). The NH_2_-terminal hydrophobic region (26 residues) is a signal peptide typical for extracellular proteins. The COOH-terminal hydrophobic region (ca. 29 residues) serves as a signal for glycosylphosphatidylinositol (GPI) modification [50] (see Section 4).

The initial homology search of RECK predicted three serine protease inhibitor-like domains in the middle portion of the protein (K1–K3 in Figure 2a): the first one (K1; residues 635–654) completely matches the consensus Kazal motif (CX_7_-C-X_6_-Y-X_3_-C-X_2,3_-C; see Figure 5 in Kawamura et al., 1998) [51], whereas the second (K2; residues 716–735) and third ones (K3; residues 754–772) partially match the motif [7]. Two separate regions in the middle showing weak homology to the epidermal growth factor-like repeat were also detected (E1 and E2 in Figure 2a; residues 493–523 and 676–709) [7]. The NH_2_-terminal one-third of the protein contains five repeats of a putative cysteine knot motif (C_2_-X_7–8_-C-X_3_-C-X_12–22_-C-X_9–12_-C; see Figure 3A in Chang et al. [44]) that comprise residues 37–84, 104–141, 151–197, 216–263, and 292–338 in the 971 amino acid RECK protein (CC1-CC5 in Figure 2a) [7]. Note that some authors abbreviate the “CC domain” as “CK domain” [52]. The NH_2_-terminal one-third of RECK also contains five potential N-glycosylation sites, asparagines at positions 39, 86, 200, 297, and 352 (G1–G5 in Figure 2a) [7].

An early study by Oh et al. [13] indicated that transfection of an *RECK*-expression vector into the human fibrosarcoma cell line HT1080 reduced the amounts of active MMP2 and MMP9 in culture supernatant as detected by gelatin zymography. Experiments to elucidate the domain of RECK required for this activity using a series of point mutants and deletion mutants of *RECK* cDNA were not so informative, since the activity was lost in every mutant. The activity was also lost by some of the N-glycosylation site mutations (Shimizu et al. [53]). This led to the speculation that RECK may be readily misfolded and inactivated when amino acid substitutions or deletions were introduced, and consequently, production and purification of the near-full-length, fully glycosylated recombinant RECK protein are required for biochemical characterizations. This goal was achieved by expressing NH_2_-terminally His-tagged RECK protein (RECK-His) in mouse embryo fibroblasts with null mutations in RECK and the proteases MMP2 and MT1-MMP, which are known to cleave the RECK protein [49]. Single-particle reconstruction of purified RECK-His using transmission electron microscopy and extensive image analyses revealed a unique cowbell-like shape (an oval surface with one end open and the other end closed) formed by RECK dimers [49] (Figure 2b). RECK was found to competitively inhibit MMP7-mediated cleavage of fibronectin (see Section 6.1).

In contrast to the system described above, Chang et al. [54] produced a COOH-terminal RECK fragment (termed K23; size: ~22 kDa) starting from residue 606 and containing the K2 and K3 domains (but not the K1 domain). The purified recombinant protein directly interacted with MMP9 and inhibited MMP9 activity in vitro, and the recombinant protein also suppressed the production of MMP9 by human lung cancer cell lines in culture. In addition, the recombinant RECK protein suppressed invasion by human lung cancer cell lines in culture. Of note, a larger fragment (K123) containing all three Kazal motifs failed to show these activities, suggesting that the K1 segment somehow hinders the activities of K23. Chang et al. also reported that full-length RECK inhibited MMP9 activity and production, suggesting that the inhibitory effect of the K1 segment on K23 is somehow suppressed in full-length RECK. The work raised the possibility that certain functions of RECK sub-fragments may be demonstratable even when excised out of the RECK protein. It remains unclear, however, if the 3D-structure of K23 reasonably resembles that of the corresponding region of the intact RECK protein.

Another structural domain of RECK called frizzled cysteine-rich domain (FZ-CRD) was detected by a domain prediction tool, HHpred, although its functional significance remains to be elucidated [55].

Raising reliable antibodies against RECK has been a challenge. The mouse monoclonal antibody 5B11D12 raised against bacterially expressed COOH-terminal fragments of the human RECK protein (residues 801–971) which detects the monomer band of ~125 kDa protein [7] has been the first choice for many years despite its limitations; this reagent often gives rise to high molecular weight background bands in immunoblot assays and is difficult to use in mouse histology (since it is mouse IgG). A more recently developed rabbit monoclonal antibody, D8C7 (Cell Signaling Technology #3433, Danvers, MA, USA), may solve these problems.

Of note, RECK is structurally unrelated to the other group of well-characterized endogenous MMP inhibitors, the tissue inhibitor of metalloproteinase (TIMP) family, consisting of four members (TIMP-1 to 4) [56]. Mice lacking all TIMPs (quadruple mutant) may survive more than 10 days after birth [57], which is in sharp contrast to the mid-gestation death of *Reck*-deficient mouse embryos, suggesting substantial differences between the biological functions of these two groups of endogenous MMP inhibitors.

### 3.2. RECK-Interacting Proteins

Several potential RECK-interacting proteins have been reported (Table S2). Mori et al. [58] detected the interaction of an oncoprotein, Tgat (through its unique COOH-terminal 15 amino acid residues), with RECK (residues 160–641) by yeast two-hybrid screening, followed by confirmation by co-immunoprecipitation experiments. Tgat is a splice variant of TRIO, a guanine nucleotide exchange factor (GEF) for the small G-protein Rho, which is localized to the cytosol. It is well-known that cytosolic proteins are synthesized by cytosolic ribosomes and remain in the cytosol after synthesis, while extracellular proteins are synthesized by ribosomes attached to the ER (the rough endoplasmic reticulum), which are processed in the ER, transported to the Golgi, sorted into vesicles associated with the secretory pathway, and secreted. When extracellular proteins are internalized, they remain within membrane vesicles and, in the absence of specific mechanisms that are exhibited by proteins such as protein toxins and viruses, they do not enter the cytosol [59,60]. Consequently, cytosolic proteins and secretory proteins do not come into contact with each other, except when proteins in the ER are targeted for degradation by a pathway known as Endoplasmic Reticulum-Associated Protein Degradation (ERAD) [61,62]. Thus, the proposed interaction between the extracellular (RECK) and cytosolic (Tgat) proteins may need to be verified as to where and how the interaction occurs physiologically. Fluorescently tagged proteins or Förster Resonance Energy Transfer (FRET) may be useful in addressing this issue.

Chen et al. [63] identified a protein co-precipitated with RECK as glucose-regulated protein-78 (GRP78) by liquid chromatography tandem mass spectrometry (LC-MS/MS). The binding of full-length as well as a COOH-terminal fragment of RECK (residues 781–971; see Figure 2a) with GRP78 was also confirmed by co-immunoprecipitation assays. GRP78 (also known as HSPA5) is a member of the HSP70 family involved in the folding and assembly of proteins in the ER and is sometimes called “a master regulator of ER homeostasis”. Since GRP78 contains a signal peptide, its subcellular localization is topologically consistent with that of RECK; indeed, their colocalization in cultured cells was demonstrated by co-immunofluorescent staining. In neuroblastoma cells, overexpression of RECK (but not of RECK lacking the COOH-terminal region) increased the phosphorylation of PERK and eIF-2a [hallmarks of the unfolded protein response (UPR) that occurs during ER stress] and sensitized the cells to a cytotoxic anticancer drug, cisplatin. These effects of RECK were suppressed by GRP78 overexpression. Although the work sheds new light on the function of RECK, how the direct binding of RECK and GRP78 affects their functions remain to be clarified. For instance, it is unclear whether the observed effect of GRP78 to suppress phosphorylation of PERK and eIF-2a reflects suppression of RECK function or suppression of ER stress independently of RECK.

Walsh et al. [64] used an antibody array (against extracellular or membrane-bound proteins, *n* = 119) to identify proteins that were co-immunoprecipitated with RECK from breast cancer cell lysates and detected five proteins: β1-integrin (ITGB1), galectin-1 (GAL1), interleukin-6 receptor (IL6R), gp130 (IL6ST), and urokinase-type plasminogen activator receptor (uPAR). These and other data led the authors to propose that RECK regulates STAT3 signaling. Subcellular localization of these proteins matches that of RECK, and their interactions with RECK are feasible and intriguing. Whether these interactions affect RECK’s function and which domains of RECK are involved in these interactions are among the questions to be addressed in future studies.

Functional links among RECK, GPR124, and WNT7 were first described by Vanhollebeke et al. [65]. A physical association between RECK and GPR124 was first demonstrated by Vallon et al. [66]. Cho et al. [67] identified a domain of RECK (CC1) involved in this interaction, and three amino acid residues critical for the interaction (Figure 2a, red residues in the left callout box). Eubelen et al. [52] first suggested direct binding of RECK with WNT7 and identified a region of RECK (CC4-CC5) involved in this interaction; Cho et al. [68] reported two amino acid residues in CC4 critical for this interaction (Figure 2a, red residues in the second callout box). See Section 6.5 for further discussion on the roles of RECK and GPR124 in WNT7 signaling.

Matsuzaki et al. [43] using a yeast two-hybrid screen detected a disintegrin and metalloproteinase with thrombospondin motifs 10 (ADAMTS10) as a potential RECK interactor (see Section 6.1 for more details). Their two-hybrid assays with sub-fragments indicated the involvement of a central region of RECK in this interaction (see Figure 2a).

Lin et al. [69] reported that collapsin response mediator protein 2 (CRMP2; also known as DPYSL2) is downregulated in various types of breast cancers and that CRMP2 overexpression suppressed cell invasion. In an attempt to elucidate its mechanism of action, they detected potential interaction of CRMP2 with RECK by co-immunoprecipitation. CRMP2 is a cytosolic phosphoprotein involved in the regulation of cytoskeletal dynamics, and its interaction with the extracellular protein RECK is counterintuitive as discussed above. Thus, the biological significance of this interaction awaits further evaluation.

Using a mouse model of liver fibrosis, Wei et al. [70] demonstrated the ability of RECK to suppress carbon tetrachloride-induced acute liver injury and to co-immunoprecipitate with Kelch-like ECH-associated protein 1 (KEAP1; a component of E3 ubiquitin ligase complexes). They proposed that RECK might sequester KEAP1 to liberate and stabilize a transcription factor, E2-related factor 2 (Nrf2), which is crucial for cellular defense against oxidative stress. Although the reported biological activity is intriguing, the RECK–KEAP1 interaction has the same topological problem as discussed above for the RECK–Tgat interaction (extracellular vs. cytosolic). Therefore, the physiological relevance of the proposed RECK–KEAP1 interaction requires further evaluation.

Heiden et al. [71] performed unbiased screening for RECK-associated proteins in a mouse brain endothelial cell line (bEnd.3). They expressed biotin-tagged RECK (achieved by co-expression of BAP-tagged RECK and a bacterial biotin–ligase in the cell) followed by chemical crosslinking, streptavidin pulldown, and identification of dissociated proteins by mass spectrometry. This method detects proteins present in close proximity to RECK but cannot distinguish between direct and indirect association. Nevertheless, they confirmed the association of RECK with GPR124, WNT7A, and some, but not all, components of WNT receptors (see Section 6.5 for more details). The total of 135 “RECK-associated” proteins they detected includes molecules relevant to cell–cell adhesion, cell–matrix adhesion, growth factor binding, and transmembrane receptor protein kinase activity (see Table S3 of Heiden et al., 2025 [71]).

### 3.3. Three-Dimensional Structure of the CC4 Domain

Chang et al. [44] determined the crystal structure of the CC4 domain of mouse RECK at 1.65 Å resolution and found that it folds into a compact four-helix bundle with three disulfide bonds (Figure 2c,d). They could confirm the surface locations of critical residues that were shown in previous mutagenesis studies [67] to mediate GPR124 binding and WNT7A/WNT7B recognition and signaling. Surprisingly, homology searches at both the sequence and structural levels detected no other cell-surface or secreted domains in vertebrates that resemble the CC domain, a pattern that is in striking contrast to other ancient domains of similar sizes, such as the epidermal growth factor, fibronectin type 3, immunoglobulin, and thrombospondin type 1 domains, which are found in hundreds of proteins. Their database searches also indicated that (1) RECK homologs are present as a single gene in a wide variety of metazoan species such as snails, mussels, sea anemones, corals, and sponges but absent in nematodes, plants, fungi, and choanoflagellates, (2) the CC domains are found only in the RECK protein, and (3) CC domains usually repeat five times, with one exception in the sponge *Amphimedon queenslandica*, which has a RECK homolog with three CC domains.

Taking advantage of the uniqueness of the RECK CC domains, Zhu et al. [72] used phage display to screen a library of CC4 variants (having substitutions in helices B and C) and successfully obtained clones exhibiting high-affinity binding to several cell surface proteins. Their study demonstrated the utility of this domain as a scaffold to generate general purpose protein-binding reagents.

WNT proteins are known to be enzymatically lipidated by porcupine (PORCN) in the endoplasmic reticulum and bind the WNT ligand secretion mediator (WLS) protein for intracellular transport and secretion. Qi et al. [73] used cryo-electron microscopy to determine the structure of the ternary complex, consisting of WLS, WNT7A, and the CC4 domain of RECK (WNT7A-WLS-RECK^CC4^) at 3.2 Å resolution. The data indicated that the interface between WNT7A and RECK^CC4^ is of substantial size (>700 Å^2^) and that the loop connecting the third and fourth helices of CC4 (termed the L3 loop; Figure 2d) plays a crucial role in mediating this interaction. They also found that the residues of WNT7A that are responsible for RECK^CC4^ recognition are conserved in WNT7B but not in other WNT family proteins (*n* = 17), highlighting the specificity of the interaction between WNT7 and RECK. Their results also indicate that the interaction between RECK and WNT7A does not interfere with binding of WNT7A with its classical receptor components, frizzled (FZD) and low-density lipoprotein receptor-related Protein 5 and 6 (LRP5/6) [73,74].

## 4. Subcellular Localization

RECK is a GPI-modified protein (Figure 2). GPI anchors RECK to the plasma membrane, and RECK is released from the cells when the cells are treated with phosphatidylinositol-specific phospholipase C (PI-PLC) [7]. Immunofluorescent staining of fibroblasts, however, revealed that in addition to its cell surface localization, a substantial fraction of RECK was also localized in the subnuclear region of the cell [75]. The dynamic nature of RECK’s subcellular localization was demonstrated by time-lapse video-microscopy of the cells expressing RECK tagged with green fluorescent protein (GFP-RECK) [75]. Abundant and transient association of GFP-RECK with membrane ruffles and intracellular vesicles moving from the cell periphery toward the perinuclear region could be observed (see Supplementary Movie 1 to Morioka et al., 2009 [75]), suggesting that the perinuclear RECK is probably associated with intracellular membranes and includes both newly synthesized proteins being transported to the cell surface and the proteins that have been internalized within membrane vesicles.

Miki et al. [76] found that RECK could bind two metalloproteases, MT1-MMP and CD13, and proposed a model, with some supporting evidence, that RECK negatively regulates the activity of these proteases on the cell surface by promoting their internalization through an endocytic pathway that involves the “GPI-anchored protein-enriched early endosomal compartments (GEECs)”.

## 5. Tissue Distribution

In their initial study, Takahashi et al. [7] performed RNA blot hybridization and could detect *RECK* mRNA of about 4.6 kb in a wide variety of human organs as well as non-tumorigenic cells (such as human MRC5 and mouse NIH3T3) but not in tumor-derived cell lines or NIH3T3 cells that were transformed by various oncogenes (*ras*, *fos*, *myc*, *src*, *fms*, *fes*, and *mos*) [7]. This finding has been extended in two ways: (1) to examine clinical specimens to see if RECK downregulation could be a prognostic indicator (see Section 7) and (2) to elucidate the mechanism by which RECK is downregulated in cancer cells (see Section 8).

In general, RECK expression tends to be high in embryos and declines after birth. In rodent embryos, RECK expression is abundant in mesenchymal cells, e.g., fibroblasts [7], vascular cells (both endothelial and mural) [13,77,78], somites (myotome and sclerotome) [79,80], neuroepithelium [13,81], maxillofacial areas (where osteogenesis and tooth development are active) [82,83,84,85], and neuromuscular junctions [86]. In adult rodents, RECK expression remains relatively high in certain organs, such as the lung [87], pituitary gland (anterior lobe), and arcuate nucleus of hypothalamus [88], and can be induced in the hippocampus after transient cerebral ischemia [89,90]. RECK expression is also upregulated in the human mammary epithelium-derived cell line (MCF10A) after epithelial–mesenchymal transition (EMT) [91] induced by transforming growth factor beta (TGFβ). Abundant RECK expression is also detectable in some cells of maternal organs, such as uterine epithelium [77], trophoblasts [92], and the ovary [93].

These expression patterns are largely consistent with the known functions of RECK to regulate proteolysis and cell migration, and to promote some specific signaling events (see Section 6).

## 6. Physiological Functions

### 6.1. Protease Regulation

Although Kazal motifs [94] in the RECK amino acid sequence predicted serine protease inhibitor activity, experimental evidence indicated that RECK regulates matrix metalloproteinases (Table 1). The initial clue to this activity was found by gelatin zymography, and consequently, early studies mainly focused on its effects on gelatinases GELA and GELB (now known as matrix metalloproteinases MMP2 and MMP9, respectively) and MT1-MMP (also known as MMP14), an enzyme-activating pro-MMP2 [7,13]. In later studies, the spectrum of RECK targets expanded to other members of the MMP family (e.g., MMP7) as well as members of other metalloproteinase families (e.g., CD13, ADAM10, and ADAMTS10; see Table 1). As mentioned above (Section 3.1), full-length RECK competitively inhibits MMP7-mediated cleavage of fibronectin [49]. Since MMP7 has no accessory domains besides the catalytic domain, it is likely that RECK directly interacts with the catalytic domains of these proteases as a pseudo-substrate and that it may inhibit most, if not all, of the MMP family members, given the high degree of structural similarity in their catalytic domains.

RECK is also known to reduce the amount of several metalloproteases produced by cells. Proposed mechanisms of this effect include transcriptional repression (MMP9) [95], suppression of secretion (MMP9) [7], and enhanced internalization (MT1-MMP, CD13) [76].

Studies on the mechanism of defective neural development in *Reck*-deficient mice (see Section 6.6 for more detail) led to the finding that RECK inhibits ADAM10-mediated shedding of Notch ligands (i.e., Delta and Jagged) [81].

Through yeast two-hybrid screening of a cDNA library using RECK as a bait, ADAMTS10 was detected [43] (see Section 3.2). In vitro, RECK protects ADAMTS10 from fragmentation after chemical activation of its proform, and ADAMTS10 interferes with RECK inhibition of MT1-MMP. In cultured cells, RECK increases the amount of ADAMTS10 associated with the cells. Of note, *ADAMTS10* [96,97] is mutated in patients of a rare connective tissue disorder, the Weill–Marchesani syndrome (WMS) [98]; mutations in the fibrillin-1 (*FBN1*) gene are also found in patients of WMS [99], suggesting functional interaction between ADAMTS10 and FBN1. Fibrillin microfibrils are essential components of connective tissues, consisting of their core glycoproteins (FBNs; ~350 kDa) and several associated proteins [100,101], and have an extensible beaded-chain structure found in many elastic tissues by electron microscopy; they also serve as scaffolds for elastin fiber formation [102] and as tissue reservoirs for latent forms of TGFβ family members [103]. Mice with reduced RECK expression and mice lacking MT1-MMP show similar abnormalities in fibrillin fibers [104] (also see Section 9.4). Experiments with mutant dermal fibroblasts indicated that MT1-MMP protects RECK from degradation while RECK promotes proteolytic processing of MT1-MMP, supporting the idea that RECK and MT1-MMP play cooperative roles in fibrillin microfiber formation.

### 6.2. Cell Migration, Invasion, and Metastasis

#### 6.2.1. Findings with Normal Cells (Table S3)

Several lines of evidence indicate that RECK plays an important role in the controlled directional migration of mesenchymal cells (Table S3). Morioka et al. [75] reported that mouse embryo fibroblasts (MEFs) derived from *Reck*-deficient mice showed decreased spreading, ambiguous anterior–posterior polarity, and increased speed and decreased directional persistence in migration compared to their RECK-reconstituted counterparts. *Reck*-deficient MEFs failed to form discrete focal adhesions, had increased levels of GTP-bound Rac1 and Cdc42, and showed a marked decrease in the level of detyrosinated tubulin, which is a hallmark of stabilized microtubules. *Reck*-deficient MEFs also showed elevated gelatinolytic activity and decreased fibronectin fibrils associated with the cell. This phenotype was largely suppressed when the cells were plated on fibronectin-coated dishes. These findings suggest that RECK regulates pericellular extracellular matrix (ECM) degradation, thereby allowing the cells to form the proper cell–substrate adhesions required to maintain robust anterior–posterior polarity during migration. In addition to regulating MMP activity, RECK is a membrane-anchored protein that exhibits dynamic movement in its subcellular localization, and this property is also likely to be involved in the temporally and spatially coordinated attachment–detachment cycles between the ventral surface of the cell and the underlying substrate required for substrate-dependent cell migration.

Lee et al. [105] performed selective sequencing of mRNA 3′-ends to compare quiescent and proliferating human dermal fibroblasts and found that in proliferating cells, *RECK* variant 5 (termed “short *RECK*”) was more abundant than the full-length variant 1 (termed “long *RECK*” see Figure 1b). In quiescent cells, on the other hand, the short *RECK* mRNA was less abundant than the long *RECK* mRNA. Their survey of public databases indicated that short *RECK* is more abundant in established cell lines and various cancer tissues than corresponding normal tissues. Knockdown of short RECK in dermal fibroblasts resulted in slower migration than the control in Matrigel, whereas knockdown of long RECK resulted in faster migration, indicating that short RECK functionally counteracts long RECK and promotes cell migration in Matrigel. Interestingly, pull-down assays indicated that short RECK binds long RECK at the domain containing Kazal motifs and inhibits MMP9 binding. Fluorescence complementation assays indicated that short RECK is associated with long RECK in the ER, Golgi, and on the cell surface.

**Figure 3:**
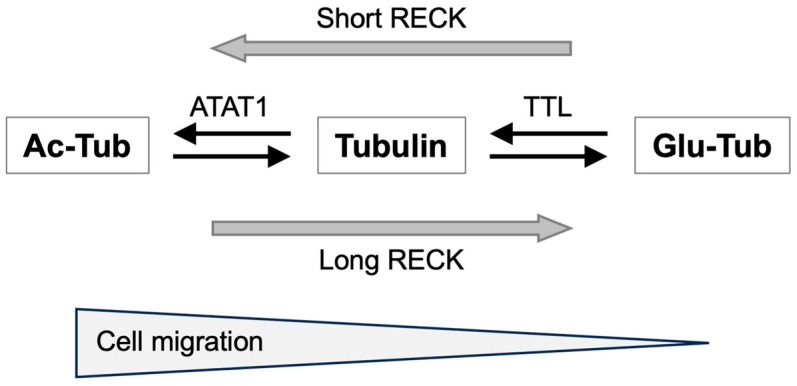
Effects of RECK isoforms on tubulin modifications. Abbreviations: Ac-Tub, acetylated tubulin; Glu-Tub, detyrosinated tubulin; ATAT1, α-tubulin acetyltransferase-1; TTL, tubulin tyrosine ligase. Based on Lee et al., 2019 [106].

The same group also reported [106] that short RECK and long RECK have opposing effects on two types of tubulin post-translational modifications, acetylation (generally associated with migration/malignancy) and detyrosination (generally associated with stability). They found that knockdown of long RECK resulted in an increase in the level of acetylated tubulin (Ac-Tub) and a decrease in the level of detyrosinated tubulin (Glu-Tub), which is consistent with the observation in *Reck*-deficient MEFs [75] (see above). Ac-Tub formation is catalyzed by tubulin acetyltransferase 1 (ATAT1) while Glu-Tub can be converted to unmodified tubulin by tubulin tyrosine ligase (TTL). Knockdown of ATAT1 and LLT (to mimic short RECK knockdown) resulted in slower migration of normal dermal fibroblasts. Knockdown of long RECK resulted in faster cell migration than the control while further knockdown of ATAT1 and LLT in these cells resulted in slower migration than the control, indicating that changes in these tubulin modifications have a direct impact on cell migration. MMP inhibitors and integrin inhibitors did affect the level of Ac-Tub but not the level of Glu-Tub, indicating that the effects of RECK on tubulin modifications (Figure 3) cannot be fully explained by MMP regulation.

Gutierrez et al. reported [107] that TGFβ1 downregulates RECK, that RECK downregulates β1-integrin expression, and that *Reck*^+/−^ mice show accelerated skin wound healing. They suggest that RECK plays a key regulatory role in skin wound contraction.

In addition, RECK is reported to suppress cell migration of normal cells associated with some disorders: for instance, cardiac fibroblasts associated with fibrosis and adverse remodeling in hypertensive heart diseases [108], aortic smooth muscle cells associated with neointimal thickening in hyperplastic vascular diseases [109] (see Section 10.1), and mesenchymal stem cells associated with breast cancer [110].

#### 6.2.2. Findings with Tumor Cells (Table 2)

Gene manipulations, such as overexpression and knockdown, in cultured cells and model animals have implicated RECK in suppression of tumor invasion and metastasis. In the original paper identifying these activities, Takahashi et al. [7] reported that RECK overexpression in mouse melanoma (B16-BL6) and human fibrosarcoma (HT1080 and its metastatic subline, RZmet-2) cell lines suppressed Matrigel invasion in vitro as well as experimental and spontaneous tumor metastasis in nude mice without affecting their proliferation or cell motility in vitro. Suppression of Matrigel invasion by RECK was also found in later studies using tumor-derived cell lines of various origins, although its effects on cell proliferation and motility seem to depend on the cell lines or experimental conditions employed (see Table 2).

Srivastava et al. [111], using the genetic model of tumor invasion in the fruit fly (i.e., clonal induction of *Ras^V^*^12^ plus loss of a cell polarity gene, *scrib*, in the eye imaginal disc) developed by Pagliarini and Xu [112], found that overexpression of both TIMP and RECK are required to prevent the basement membrane degradation prerequisite for tumor invasion in this model.

Although suppression of invasion by RECK can be explained by ECM protection, involvement of other mechanisms cannot be ruled out. For instance, Yuki et al. [91] observed increased expression of integrin alpha 5 after RECK overexpression in A549 (lung cancer) cells, suggesting effects on integrin signaling. Intact chemotactic activity supported by RECK [75] may also ameliorate the invasive behavior of cancer cells. Metastasis, on the other hand, is a multi-step process; hence, the effects of RECK on more diverse processes should also be linked to suppression of metastasis. For instance, in addition to inhibition of MMP activity [7,19,20,21,22,23,24,25,26,27,54], RECK suppression of tumor angiogenesis [13,64] and suppression of EMT [113] very likely contribute to metastasis suppression.

**Table 2:** Effects of RECK on the behavior of tumor cells.

Tumor Type	Method *	System/Cell Line	Effects (Assay)	Reference		
				First Author	Year	PMID
mouse melanoma human fibrosarcoma	OE	B16-BL6 HT1080, Rzmet-2	Suppression of invasion (Matrigel) and metastasis (tail vein, spontaneous) with no effects on cell proliferation and motility	Takahashi	1998	9789069
*Ras^V12^*/*scrib^-/-^* tumor cells	OE	Drosophila larval eye imaginal disc	Suppression of basement membrane degradation after co-overexpression with TIMP in vivo	Srivastava	2007	17301221
pituitary adenoma	KD	HP-75	Promotion of tumor invasion and proliferation (realtime-imaging/suspension culture in PuraMatrix gel containing collagen-IV)	Yoshida	2008	18493720
glioblastoma	OE	T98G	Suppression of migration (scratch), invasion (matrigel), and proliferation (soft agar)	Silveira Corrêa	2010	20127710
pancreatic epithelioid carcinoma	OE	PANC-1	Suppression of invasion (Transwell)	Tian	2010	20635007
pancreatic ductal carcinoma	KO	KC mouse	EMT, invasion, and liver metastasis	Masuda	2023	37712427
osteosarcoma	OE	SaOS-2	Suppression of invasion (Matrigel), cell proliferation (collagen-1 gel), tumorigenic growth and bone destruction (orthotopic transplantation into nude mice); promotion of cell adhesion to collagen-1	Clark	2011	21287525
prostate carcinoma	OE	DU-145	Suppression of invasion (Matrigel)	Rabien	2012	22025325
ameloblastoma	OE	hTERT^+^-AM	Suppression of migration (scratch) and invasion (Matrigel)	Liang	2014	24646032
lung carcinoma	OE	A549	Lower migration speed and increased directional persistence on FN in the presence of TGFβ (random migration). Upregulation of integrin-alpha-5; Suppression of cell proliferation and TGF-beta-induced invasion (Matrigel).	Yuki	2014	24691523
breast cancer	OE	(1) LM2-4175 (2) MDA-MB-231	Suppression of experimental metastasis to the lung [(1) tail vein] and spontaneous metastasis to the lung and liver [(2) orthotopic]; no effects on tumor growth in vitro and in vivo	Walsh	2015	24931164
	KD	Hs343T, Hs606T	Promotion of invasion (Matrigel)			
cervical cancer	OE	SiHa, SW756	Suppression of invasion (Matrigel)	Herbster	2021	34066355
ovarian cancer	KD	A2780, SKOV3	Increased viability (apoptosis markers) and mesenchymal phenotype (EMT markers)	Zheng	2021	33941323

* OE: overexpression; KD: knockdown; KO: knockout.

### 6.3. Cell Proliferation (Table S4)

RECK suppression of cell proliferation has been described in several reports (Table S4). Formal evidence indicating that RECK suppresses tumor formation in vivo was obtained only recently using mutant animals (see Section 9.4). Prior to this, the effects of RECK on cell proliferation have been examined in culture or transplantation experiments using cell lines in which the RECK gene had been manipulated (“Method” in Table S4). Several studies also addressed the mechanisms of the effects observed. For instance, Hong et al. suggested that RECK binds HER2/ERBB to prevent its dimerization in breast cancer cells [114] while in gastric cancer cells RECK inhibits ADAM17-mediated Notch1 cleavage and downstream signaling [115]. Notably, the latter activity seems to contradict the activity of RECK to support Notch signaling in neural precursor cells (see Section 6.6).

RECK has also been implicated in cellular senescence. Kitajima et al. [116] reported that mouse embryo fibroblasts (MEFs) derived from *Reck*-knockout embryos could be readily immortalized. Likewise, Yoshida et al. [117] reported that acute re-expression of RECK in the colon cancer cell line SW620 using an adenoviral vector resulted in cellular senescence accompanied by SKP2-downregulation and p27-upregulation. In addition, Lee et al. [118] found that knockdown of RECK in HEK293 cells resulted in activation of EGFR and decreased expression of CDK inhibitors (p16, p21, and p27), accompanied by increased cell proliferation and tumorigenicity.

RECK can also augment the effects of chemotherapeutic agents to kill cells. Chen et al. [63] reported that RECK overexpression enhanced cisplatin-induced cell death of neuroblastoma cell lines. They suggest that RECK binds the heat shock protein GRP78 through its C-terminal region and enhances endoplasmic reticulum (ER) stress (see Section 3.2 for more details). Hong et al. [119] reported that RECK overexpression increased the sensitivity of breast cancer (SKBR3) cells to anticancer drugs such as cisplatin, camptothecin, and etoposide with activation of ATM/ATR pathways and increased formation of γ-H2AX foci. They suggest that RECK inhibits HER2/ERBB signaling, thereby attenuating the expression of JAB1 and RAD51 to impede DNA repair. Interestingly, these anticancer drugs are known to activate the *RECK* promoter (see Section 8.4.4).

### 6.4. Vascular and Limb Development (Table S5(1,2))

**Figure 4:**
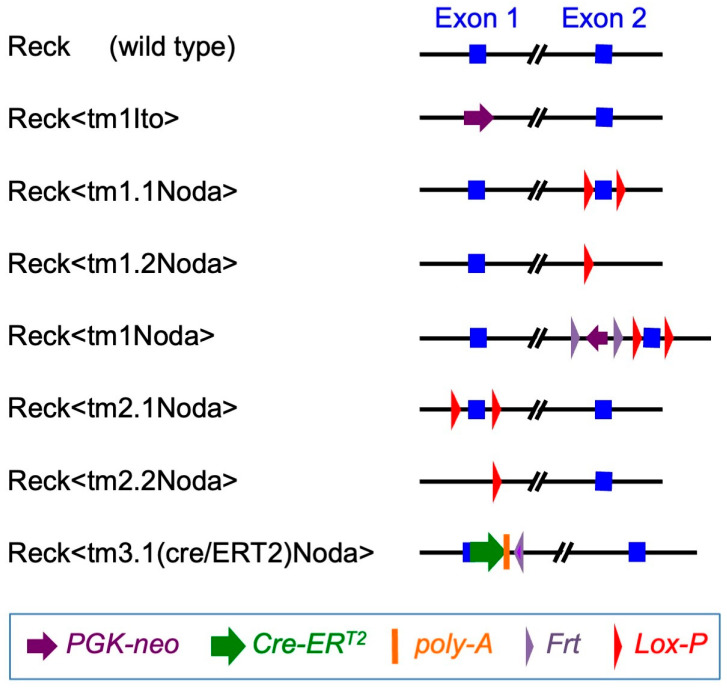
*Reck* mutant alleles generated in mice. Schematic illustration of the first two exons (blue boxes) in the wild-type allele (top) and compositions of seven *Reck* mutant alleles. Elements are not to scale. See Table S6 for allele name and reference literature.

Conspicuous phenotypes of global *Reck*-knockout embryos (Reck<tm1Ito>/Reck<tm1Ito>; Figure 4) include the arrest of vascular development after vasculogenesis: in RECK-knockout embryos, vasculogenesis occurs, but maturation of the vasculature is compromised [13]. At that stage, the primary capillary plexus becomes refined into dendritic structures through several mechanisms such as sprouting, intussusceptive angiogenesis, pruning, and luminal growth [120]. The mechanism by which RECK contributes to angiogenesis was first studied using cultured cells. Oh et al. [121] reported that TIMP2, another endogenous MMP regulator, suppressed migration of human microvascular endothelial cells (hMVECs) and this was associated with interaction of Crk with C3G, resulting in activation of RAP1 and upregulation of RECK. Miki et al. [122], on the other hand, reported that in human umbilical vein endothelial cells (HUVECs), RECK knockdown resulted in defective vascular tube formation and cellular senescence, which was associated with beta-1-integrin activation, decreased autophosphorylation of focal adhesion kinase, and increased expression of the cyclin-dependent kinase inhibitor p21/CIP1/CDKN1A.

**Figure 5:**
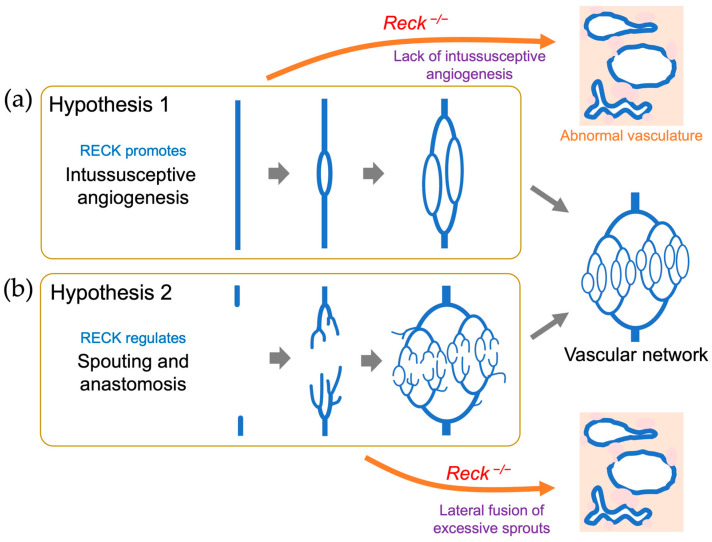
Two hypotheses on the role of RECK in vascular network formation in mice. Abnormal vasculature found in *Reck*-deficient mice can be explained by two alternative models: (**a**) lack of intussusceptive angiogenesis, (**b**) lateral fusion of small vessels. See Chandana et al., 2010 [77] and Almeida et al., 2015 [78] for more details.

Conditional knockout mice provided a more unbiased view of how RECK affects vascular development in vivo. Chandana et al. [77] used tamoxifen-inducible global *Reck* knockout embryos (Reck<tm1.1Noda>/Reck<tm1.1Noda>; CAG-CreER; Figure 4) and found that tamoxifen treatment of these animals in utero from embryonic day 11 (E11) resulted in smaller embryos with severe hemorrhage throughout the body at E15.5 with 100% penetrance. Histological examinations of pregnant, wild-type female mice revealed that RECK was abundantly expressed in the cells associated with blood vessels undergoing angiogenesis or remodeling in the uteri. Some of the RECK-positive vessels showed morphological features consistent with intussusceptive angiogenesis (lateral splitting of the vessel). Transfection of a vector expressing a small hairpin RNA against *Reck* into the uterus tissue severely disrupted the formation of blood vessels. Similar defects were found in the vasculature of global *Reck*-knockout embryos at E10.5 [13] as well as in inducible *Reck* knockout embryos (mentioned above) at E15.5 [77]. These observations led to the hypothesis that RECK plays a role in intussusceptive angiogenesis (Figure 5a).

On the other hand, Almeida et al. [78] characterized cell-type-selective *Reck* knockout mice and found that the lack of *Reck* in Sm22-positive (vascular mural) cells was largely responsible for the mid-gestation lethality found in global *Reck*-knockout embryos while the lack of *Reck* in Tie2-positive (vascular endothelial) cells led to late embryonic lethality with severe intracranial hemorrhage. The mechanism of the latter phenotype was clarified when the role of RECK in WNT7 signaling was discovered (see Section 6.5). Experiments using cultured aortic explants indicated that global *Reck* deficiency resulted in increases in the number, length, and thickness of outgrowing sprouts that seemed to undergo frequent lateral fusion [78], suggesting that the vascular phenotype of *Reck*-deficient embryos may result from abnormal sprouting angiogenesis, i.e., formation of excessive, unstable sprouts followed by their lateral fusion; Figure 5b).

**Figure 6:**
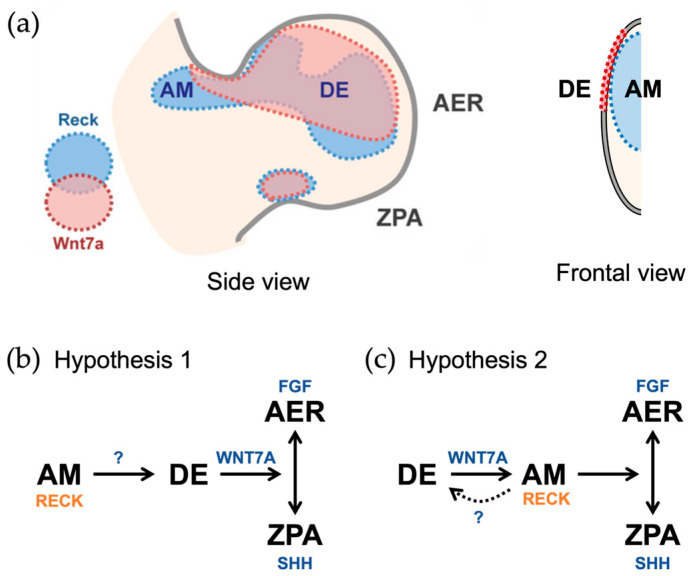
Two hypotheses on the role of RECK in limb patterning in mice. (**a**) Patterns of expression of *Reck* (blue) and *Wnt7a* (red) in the mouse limb bud at around E11.5. Abbreviations: AM, anterior mesenchyme; DE, dorsal ectoderm; AER, apical ectodermal ridge; ZPA, zone of polarizing activity. Limb abnormalities found in mice with reduced RECK expression, which are reminiscent of those found in *Wnt7a*-deficient mice, suggest two alternative mechanisms of RECK’s action: (**b**) RECK expressed by AM is required for the health of DE which produces WNT7A; (**c**) RECK in the AM is required for the signaling triggered by WNT7A produced by the DE. Abbreviations: FGF, fibroblast growth factor; SHH, sonic hedgehog. See Yamamoto et al., 2012 [123] for more details.

The role of RECK in limb development was found serendipitously. While establishing an *Reck*-floxed mouse line, Yamamoto et al. [123] unexpectedly obtained a hypomorphic *Reck* allele (Reck<tm1Noda>; Figure 4); mice hemizygous for this allele (Reck<tm1Noda>/Reck<tm2.2Noda>; Figure 4), termed Reck-Hypo mice, express RECK protein at a reduced level (about 20% or less of wild-type mice) but are viable and exhibit peculiar limb abnormalities, including right-dominant, forelimb-specific defects in postaxial skeletal elements and frequent outgrowth of nail-like protrusions on the dorsal tips of all limbs [123]. The limb phenotypes are reminiscent of those found in *Wnt7a*-deficient mice [124], providing an early indication of a possible functional link between RECK and WNT7A. This possibility was substantiated by the recent study by Wang et al. [125] demonstrating genetic interaction between *Reck* and *Wnt7a* in terms of limb phenotypes. Since *Reck* is expressed in the anterior mesenchyme (AM; Figure 6a) and the dorsal ectoderm (DE) covering the AM is severely damaged in Reck-Hypo mice, Yamamoto et al. [123] speculated (Figure 6b) that RECK might be required for the health of the DE and production of WNT7A, which is the morphogen responsible for establishing the dorsoventral as well as anterior–posterior polarities of the limb bud [126]. However, given the recent findings on the role of RECK as an important component of the WNT7 receptors (see Section 6.5) and that the AM is the target tissue receiving DE-derived WNT7A in the limb bud [126], the major role for RECK in the AM might be to bind WNT7 (Figure 6c). The damaged DE in Reck-Hypo mice, however, may suggest bidirectional interactions between the AM and the DE (dotted arrow in Figure 6c): for instance, WNT7A produced by the DE stimulates the AM to produce some factor(s) supporting the health of the DE.

### 6.5. WNT7 Signaling (Table S5(3))

Gene targeting studies by Stenman et al. [127] (2008) and Daneman et al. [128] (2009) demonstrated the importance of *Wnt7a* and *Wnt7b* for angiogenesis in the central nervous system (CNS). Kuhnert et al. [129] (2010) reported striking vascular defects and embryonic lethality of mice lacking GPR124 (also known as ADGRA2 and TEM5 [130]), a G-protein-coupled receptor whose ligand was unknown. Subsequent studies by Zhou and Nathans [131] (2014) and Posokhova et al. [132] (2015) demonstrated that GPR124 functions as a ligand-specific coactivator of canonical WNT signaling in the CNS vasculature.

A functional link between GPR124 and RECK was discovered independently by two groups. First, Vanhollebeke et al. [65] took a reverse-genetic approach (i.e., targeted mutation) in zebrafish and found that *gpr124*-deficient fish had defects in the development of dorsal root ganglia (DRG) as well as cerebral vasculature. By screening several candidate genes previously reported to be involved in DRG development by morpholino-mediated knockdown, they found that only *Reck*-targeting morpholino gave rise to a phenotype very similar to that of Gpr124-deficient fish in both DRG and cerebral vasculature. These results were in agreement with those of Prendergast et al. [133] who initially reported that *reck* was involved in DRG development (see Section 6.6). Second, Vallon et al. [66] took a biochemical approach to find GPR124-interacting proteins. A major protein that co-purified with GPR124 (using anti-GPR124 affinity chromatography) from lysates of rat brain blood vessels was identified as RECK. Both groups used the TOP-flash reporter assay to show that GPR124 and RECK cooperate in enhancing intracellular signaling, known as “canonical WNT” or beta-catenin signaling, triggered by WNT7A or WNT7B.

The importance of RECK in brain angiogenesis in zebrafish was also demonstrated independently by Ulrich et al. [134], who took a forward-genetic approach to screen mutants exhibiting brain-specific vascularization deficits (termed *no food for thought*, or *nft* in short). One such mutant, *nft^y^*^72^, turned out to be a *reck* mutant with a cysteine-to-tyrosine substitution at residue 254, with the position corresponding to cysteine-263 (at the COOH-terminal end of the CC4 domain) in the human RECK protein (Figure 2a; see below for further discussion).

Cho et al. [67] found that deletion of exon 2 in the mouse *Reck* gene [77] (Reck<tm1.2Noda>/Reck<tm1.2Noda>; Figure 4) gave rise to a hypomorphic allele less active than the allele described by Yamamoto et al. [123] (see Section 6.4). Using this allele, they demonstrated genetic interactions between *Reck* and *Gpr124* or *Ndp* (the gene encoding Norrin, a factor critical for retinal angiogenesis [135]). They also identified a stretch of amino acid residues in the RECK protein (Q^68^RAPDY^73^) important for GPR124 binding (Figure 2) and demonstrated that the alanine scanning mutation Q^68^RAPDY^73^ to A^68^AAAAA^73^, when introduced in mice (allele name: *Reck^Cr^*), partially inactivated RECK function in CNS angiogenesis, which is in agreement with the partially reduced activity of *Reck^Cr^* to support WNT7 signaling in the TOP-flash assay [132].

**Figure 7:**
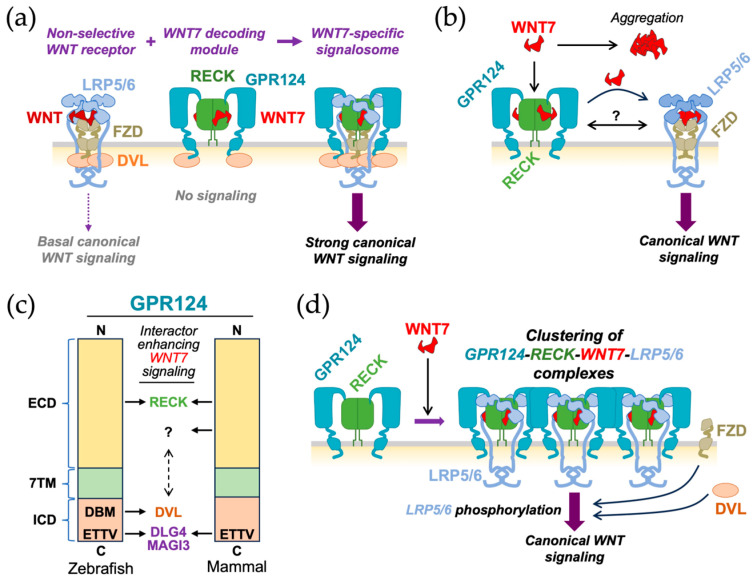
The role of GPR124 in RECK-mediated WNT7 signaling. (**a**) A model based on the findings reported by Eubelen et al. [52]. The FZD-LRP5/6 complex serves as a WNT-dependent signaling module with low ligand selectivity and a basal level of canonical WNT signaling (**left**), while the RECK-GPR124 complex selectively binds WNT7 without signaling, acting as a WNT7-decoding module (**middle**). WNT7 induces physical association between these two modules and consequent DVL recruitment to transmit strong canonical WNT signaling (**right**). (**b**) A model based on the findings reported by Vallon et al. [66]. This model proposes that nascent WNT7 tends to form biologically inactive aggregates unless rapidly trapped by RECK-GPR124 complexes. This complex then relays the active monomeric ligands to the signaling receptor (FZD-LRP5/6). Physical association between RECK-GPR124 and FZD-LRP5/6 (double-headed arrow) is a possibility. (**c**) Comparison between zebrafish and mammalian GPR124 [141]. Both zebrafish (**left**) and mammalian (**right**) GPR124 proteins can interact with RECK through their ECDs and with DLG4 and MAGI3 through the ETTV motifs in their ICDs. Only zebrafish GPR124 can interact with DVL through its ICD (i.e., DBM domain), while a soluble ECD fragment of mammalian GPR124 can support WNT7 signaling, suggesting that the ECD of mammalian GPR124 can functionally substitute for the DVL-binding activity of zebrafish GPR124 which is missing in the mammalian GPR124-ICD. (**d**) A model based on the findings reported by Heiden et al. [71]. In this model, RECK constitutively associates with GPR124, and WNT7 triggers formation of the GRP124-RECK-WNT7-LRP5/6 core complex as well as clustering of core complexes due to the ability of GPR124 to form multimers. RECK is usually found in its dimeric form [49]. In panels (**a**,**b**,**d**), the name and the symbol of each protein are color matched. These figures are intended as an aid for the verbal explanation given in Section 5; the shapes of the proteins as well as their stoichiometry and steric configurations are largely hypothetical.

Eubelen et al. [52] reported several lines of evidence indicating that RECK directly binds WNT7A/WNT7B to confer ligand specificity to the core WNT receptor complex, Frizzled/LRP5/6 (FZD/LRP5/6) (Figure 7a). Cho et al. [68] found that two evolutionally conserved amino acid residues (P256 and W261) in the CC4 domain (Figure 2a,c) were required for WNT7A-FZD-GPR124-RECK complex formation and signaling. When they introduced the P256A/W261A mutations into mouse *Reck* by gene editing (allele name: *Reck^P256A^*^,*W261A*^), homozygous mutants exhibited mid-gestation lethality reminiscent of *Reck*-null mice [13]. These and other data suggest that the *Reck^P256A^*^,*W261A*^ allele is functionally null, implying that the most critical function of RECK at the mid-gestation stage in mice is to enhance WNT7 signaling in vascular endothelial cells. However, previous findings by Almeida et al. [78] using tissue-specific gene targeting indicated that *Reck* inactivation in vascular mural cells (rather than endothelial cells) was critical for mid-gestation lethality (see Section 6.4), and their data implicated excessive proteolysis in the phenotype. Hence, the cause of death of the global *Reck*-deficient embryos remains controversial.

Given the 3D structure of CC4 and the positions of residues P-256 and W-261 and the L3 loop critical for WNT7 interactions (see Section 3.3; Figure 2c,d), we can now speculate on how the zebrafish *nft^y72^* mutation phenocopies a *Wnt7* mutation. Since the *nft^y72^* mutation occurs at the last conserved cysteine residue (cysteine No. 6 in Figure 2c) within alpha-helix D of CC4, it is likely that the resulting loss of the disulfide bond (the C-C bond) between alpha-helices A and D could disrupt the correct configurations of P-256 and W-261 in helix D and loop L3 (connecting alpha-helices D and C), thereby disrupting the interaction of RECK with WNT7.

How could RECK enhance WNT7 signaling? Lipid-modified (hydrophobic), monomeric, and active WNT7A/WNT7B ligands tend to form inactive aggregates in aqueous solution. Vallon et al. [66] reported that RECK binds these ligands and keeps them monomeric and active (Figure 7b). This model nicely explains the unexpected finding by Li et al. [136] that *Reck* knockout in *Foxg1*-positive neural precursor cells (NPCs), a source of WNT7 ligands, and *Reck* knockout in *Tie2*-positive vascular endothelial cells, a receiver of WNT7 ligands, gave rise to very similar phenotypes in mice: that is, neonatal death with forebrain hemorrhage [67,78]. This is understandable if we assume that newly produced, lipid-modified, monomeric WNT7 ligands are rapidly trapped and stabilized by the RECK on the surface of neural precursor cells and delivered to the RECK on the surface of adjacent endothelial cells to achieve successful receptor binding and downstream signaling. This raises the interesting possibility that RECK serves as a mediator of neurovascular association in the brain.

WNT7 ligands are capable of transmitting signals through two pathways: the RECK-dependent pathway required for CNS angiogenesis/BBB-formation and an RECK-independent (but FZD-dependent) pathway evoking more ubiquitous effects. As a step toward the clinical application of this line of findings, Martin et al. [137] generated a mutant WNT7A (WNT7A^K190A^) with augmented RECK/GPR124-dependent signaling and minimum FZD5-dependent signaling. The gene could be expressed efficiently in mouse brain using an adeno-associated viral (AAV) vector as a BBB-specific WNT activator. Notably, AAV delivery of WNT7A^K190A^ proved effective in mitigating glioblastoma expansion as well as ischemic stroke infarction in mouse models. Since, WNT7A^K190A^ showed reduced stability, the authors speculated that its binding with RECK might increase its stability, leading to selective augmentation of RECK/GPR124-dependent signaling [137].

How does WNT7 induce brain angiogenesis? Using zebrafish, Schevenels et al. [138] found that MMP25, a protease capable of cleaving type IV collagen, is a critical target induced by WNT7 signaling via the GPR124-RECK complex in endothelial tip cells. The finding seems to suggest the dual roles for RECK in this context: RECK, a negative regulator of MMPs, is used to activate the expression of an MMP. Parab et al. [139] also used zebrafish to demonstrate that *Reck* inactivation leads to defects in the glut1-positive vasculature (with the blood–brain barrier) but not in the plasmalemma vesicle-associated protein (PLVAP)-positive vasculature (fenestrated) in the choroid plexus and that the latter requires VEGF signaling, shedding new light on the functional and developmental heterogeneity of brain vasculature. Chen et al. [140] reported that an RNA helicase, DDX24, activates GPR124/RECK-mediated WNT signaling in brain endothelial cells in zebrafish and that it involves upregulation of GPR124 and WNT7 but not RECK.

What is the role of GPR124 in this system? Cho et al. [67] used a cell-based binding assay to demonstrate that a multi-protein complex consisting of WNT7, FZD, LRP5/6, GPR124, and RECK is involved in efficient WNT7 signaling. Eubelen et al. [52] used Crispr/Cas9-medicated gene disruption and found that FZDs, LRP5/6, and GPR124 were dispensable, but RECK was required for WNT7A binding to HEK293 cells, while all these components were essential for WNT7 signaling. Moreover, RECK was found to inhibit WNT7 signaling in the absence of GPR124, raising the possibility that GPR124 switches ON the inert ligand–receptor complex. Their data also indicate that the intracellular domain (ICD) of GPR124, which is essential for its function in zebrafish, can interact with Dishevelled (DVL), a cytoplasmic adaptor protein involved in canonical WNT signaling (Figure 7a).

On the other hand, Vallon et al. [66] proposed that RECK (complexed with GPR124) on WNT7-producing cells binds and relays WNT7A to the classical WNT receptor complex (FZD-LRP5/6) to transmit downstream signaling (Figure 7b). Moreover, they found that a soluble, extracellular domain (ECD) fragment of GPR124 could fully support WNT7/RECK-mediated WNT signaling in HEK293 cells, which apparently contradicts the finding by Eubelen et al. [52] that the ICD of GPR124 was essential for its activity in zebrafish. This debate was followed up by America et al. [141] who compared the activities of zebrafish and mammalian GPR124 and found that the conserved C-terminal four amino acids (ETTV; a PDZ-binding motif) interacted with DLG4 and MAGI3 and enhanced the basal activity (i.e., WNT7 signaling and phenotypic rescue of gpr124-deficient zebrafish) of both zebrafish and mammalian proteins; however, the less conserved “DVL-binding motif (DBM)” was essential for the basal activity of zebrafish GPR124 but not of mammalian orthologs. They also found that, unlike the mammalian ECD fragment (see above), the zebrafish ECD fragment failed to support WNT7 signaling, leading to the speculation that the mammalian ECD might have acquired an activity that functionally substitutes for the DVL-recruiting activity associated with the zebrafish ICD (Figure 7c). Yuki et al. [142] reported that mice homozygous for a *Gpr124* mutation that truncates the ICD (termed *Gpr124*^∆*C*^) showed attenuated WNT signaling, but some animals were born and survived. This phenotype, which is milder than the phenotype of corresponding zebrafish mutant, might reflect the loss of C-terminal ETTV motif reported to be required for the full activity of GPR124 [141].

Heiden et al. [71] used a mouse brain endothelial cell line (bEnd.3) and made two interesting observations. First, knocking out either *Reck*, *Gpr124*, or *Lrp5/6* abolished WNT7 signaling, while knocking out all FZD family members or all DVL family members did not completely abolish WNT7 signaling. Second, their unbiased screen for RECK-associated proteins (see Section 3.3) indicated (1) constitutive association with GPR124, (2) WNT7-dependent and GPR124-independent association with LRP5/6, (3) no association with FZD family members or DVL family members. Overall, their data support the model that, rather than directly transmitting intracellular signaling, mammalian GPR124 promotes WNT7-induced LRP5/6 phosphorylation and downstream WNT signaling by promoting the clustering of the GPR124-RECK-WNT7-LRP5/6 core complex through its ECD binding of RECK and presumably forming homodimers/oligomers like other G protein-coupled receptors [143] (Figure 7d). This model nicely explains the ability of the mammalian GPR124 ECD to functionally substitute for the DVL-recruiting activity of zebrafish GPR124 ICD (“?” in Figure 7c) postulated by America et al. [141]

Thus, researchers agree that RECK directly binds WNT7 and that RECK, GPR124, and LRP5/6 play central roles in WNT7 signaling; however, the mechanisms of actions of GPR124, FZDs, and DVLs in WNT7 signaling remain controversial. Some of the discrepancies may be due to the difference in experimental systems (e.g., animal species, cell types) and techniques (e.g., gene manipulation in vivo, reporter assay in cultured cells, protein co-precipitation, protein crosslinking). Involvement of multiple mechanisms is also a feasible reason for the discrepancies in the reported data.

### 6.6. Neural Development (Table S5(4))

**Figure 8:**
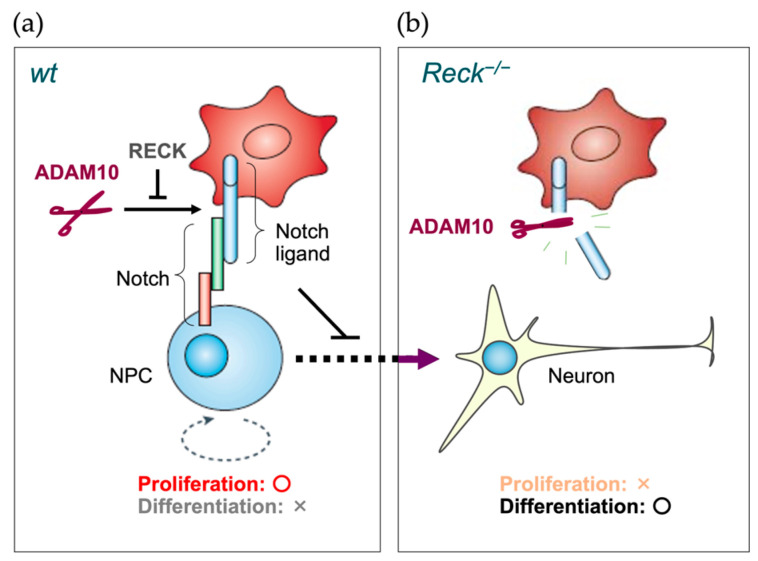
A role for RECK in neurogenesis. (**a**) RECK promotes proliferation and suppresses differentiation of NPCs by inhibiting ADAM10-mediated shedding of Notch ligands. (**b**) Absence of RECK results in precocious neuronal differentiation of NPCs. See Muraguchi et al. [81] for more details.

A prominent feature of *Reck*-deficient mice is their fragile neural tube with thinner-than-normal neuroepithelium [13]. Muraguchi et al. [81] found that this phenotype was similar to that of *Hes1*/*Hes5* double knockout mice. Since *Hes1* and *Hes5* are direct targets of, and transcriptionally activated by, Notch signaling, this observation implicated RECK in Notch signaling. Notch signaling is known to suppress neuronal differentiation and to promote self-renewal of neural precursor cells (NPCs) and RECK was found to be expressed in Nestin-positive NPCs in normal mouse embryos. Delta and Jagged expressed in surrounding cells are known to act as juxtacrine Notch ligands. Biochemical evidence suggested that RECK inhibits ADAM10-mediated shedding of Notch ligands, thereby supporting proliferative Notch signaling in NPCs. According to this model (Figure 8), the lack of RECK would result in insufficient proliferation and precocious neuronal differentiation of NPCs, which nicely explains the thin and fragile neuroepithelium found in *Reck*-deficient mice. At later developmental stages, RECK in NPCs is involved in WNT7 signaling as discussed above [136] (Section 6.5), indicating that RECK plays multiple roles even in single types of cells at different developmental stages.

**Figure 9:**
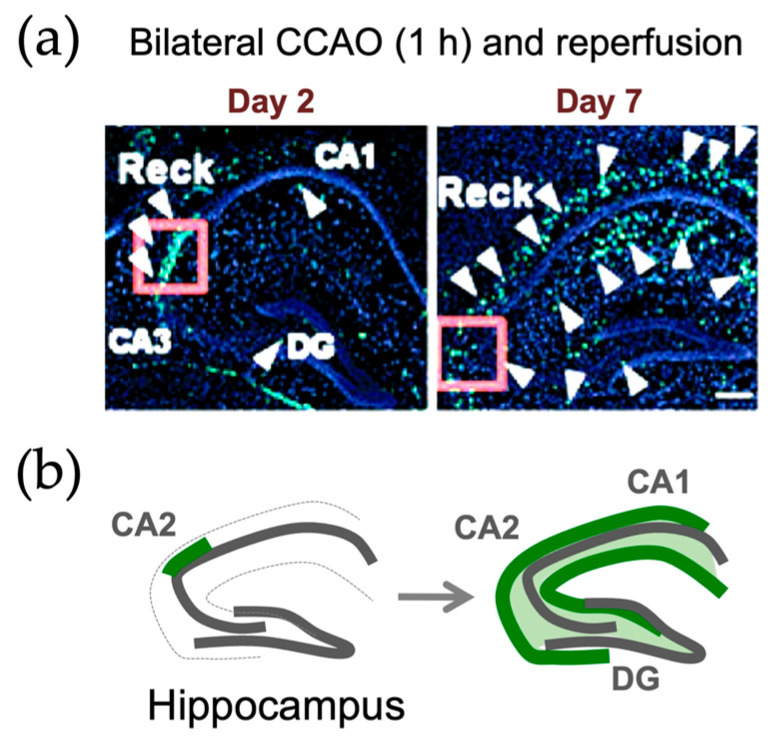
RECK-positive cells in the hippocampus after transient brain ischemia. (**a**) RECK immunoreactivity (green dots in the areas indicated by arrowheads) in the mouse hippocampus on day 2 and day 7 after transient cerebral ischemia (bilateral common carotid artery occlusion) followed by reperfusion (1 h) [89]. (**b**) Summary of the findings. RECK-positive cells are confined to the CA2 region on day 2 but are found in wider areas on day 7.

In adult mice, Wang et al. [89] found by immunohistochemistry that RECK was upregulated in the hippocampus and penumbra of the subventricular zone after transient cerebral ischemia. Most of the RECK-positive cells found on day 2 after transient ischemia were positive for Nestin as well as Ki67 and localized to the CA2 region of the hippocampus. On day 7 after transient ischemia, the RECK-positive cells increased in number, extended processes, expressed a reactive astrocyte marker (GFAP) as well as a neuronal marker (NF200), and were widely distributed in the hippocampus (Figure 9). In *Reck*^+/−^ mice, tissue damage and cell death after cerebral ischemia were augmented, and functional recovery was retarded. Hence, RECK may not only help protect tissue integrity after ischemia but also promote adult neurogenesis and tissue repair in response to tissue damage in the brain. Matsuzaki et al. [90] generated a mouse line carrying a new allele of *Reck* (Reck<tm3.1(cre/ERT2)Noda>; Figure 4) in which a regulatable Cre recombinase (CreERT2) is expressed under the control of the *Reck* promoter. They used this allele in a Cre reporter (mTmG) mouse to find that RECK was upregulated in the hippocampus after ischemia at the transcriptional level. In a more recent study using single-cell RNA sequencing, Zhang et al. [144] found that in a rat model of transient cerebral ischemia, repetitive transcranial magnetic stimulation (a potential treatment after stroke) upregulated *Reck* mRNA in vascular smooth muscle cells, suggesting that vascular RECK may also play a role in tissue repair and/or functional recovery after ischemic brain damage.

As for the peripheral nervous system, Prendergast et al. [133] attempted to identify genes affecting cell fate specification in the neural crest by performing a forward genetic screen for mutations causing DRG deficiencies in zebrafish. They identified *reck* as the target of several mutations, termed *sensory deprived* (*sdp*), found in this screen. Based on detailed observations of these mutant fish, the authors proposed that RECK was essential for proper migration of sensory neuron precursors.

Park et al. [145] provided evidence indicating that the six-transmembrane protein glycerophosphodiester phosphodiesterase 2 (GDE2) acts as an enzyme releasing RECK from the cell surface by GPI-anchor cleavage, thereby attenuating Notch signaling and promoting differentiation of spinal motor neurons. Hence, RECK seems to play multiple roles in neural development as well as brain homeostasis depending on stage and area.

### 6.7. Musculoskeletal Development (Table S5(5,6))

**Figure 10:**
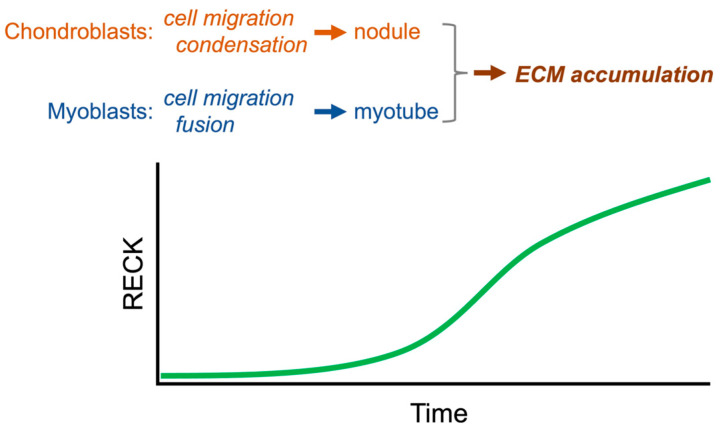
Profiles of RECK expression during chondrogenic and myogenic differentiation. *RECK* mRNA in a chondrogenic cell line and RECK protein in a myogenic cell line show similar profiles during differentiation: low expression in the early stage and progressive increase in the later stage. This may reflect the negative effects of RECK on active cell migration and condensation (chondrocytes) or fusion (myoblast) in the early stage and the positive effects of RECK on ECM accumulation in the later stage of differentiation (e.g., type II collagen in the case of cartilage formation and basement membrane in the case of myotube maturation).

Before conditional knockout mice were established, attempts were made to determine the tissue distribution of RECK in mouse embryos at various developmental stages as well as in adult mice to obtain clues to its physiological functions. Using in situ hybridization (ISH) to detect *Reck* mRNA in mouse embryos, Kondo et al. [80] found that the sclerotome and condensing cartilage were the most prominent sites of *Reck* expression at stages E13.5 and E14.5. The result was later confirmed by lineage tracing [90]. In the chondrogenic cell line ATDC5, both RECK overexpression and RECK depletion resulted in suppression of cartilaginous condensation. Experimental evidence implicated RECK in suppression of cell migration in the early stage of chondrogenic differentiation, and promotion of ECM accumulation in the later stage of chondrogenic differentiation (Figure 10).

Echizenya et al. [79], on the other hand, found that developing skeletal muscle (MRF4-positive cells) was the most prominent site of RECK expression in mice at E13.5 and E14.5 as detected by immunohistochemical (IHC) staining. In the myogenic cell line C2C12, *Reck* promoter activity was repressed by MyoD but activated by MRF4. Myotube formation by this cell line was suppressed by RECK overexpression. When whole embryo cells were cultured, the cells from *Reck*-null embryos formed myotubes more efficiently than the cells from normal embryos. Since MyoD and MRF4 are known to function at early and late stages of myogenic differentiation, respectively, the above observations suggest that RECK suppresses early events (e.g., myoblast fusion) and promotes late events (e.g., ECM accumulation) during myogenic differentiation (Figure 10).

Although the overall RECK expression declines after birth, relatively abundant RECK expression persists in some confined regions in adult mice such as in several areas in the brain (Section 6.6 and Section 6.9) and neuromuscular junctions (NMJs). Time course studies by Kawashima et al. [86] focusing on the mouse diaphragm indicated that RECK immunoreactivity became concentrated around the NMJs in the late embryonic stage (from around E18.5). Since NMJ maturation, which involves nerve apposition on nicotinic acetylcholine receptor clusters and secondary fold formation (invagination of the post-synaptic membrane), occurs by E18.5, RECK may have a role in NMJ formation and/or maintenance, possibly by protecting pericellular components, such as synaptic basal laminae and cell surface molecules, from proteolytic degradation.

Pézeron et al. [146] reported that Drosophila *Reck* contains an enhancer that contains binding sites for the transcription factor Suppressor of Hairless (Su(H)) and is a direct target of Notch signaling in muscle progenitor cells. *Reck* knockdown during embryogenesis resulted in flight deficiency (“held out wing”), suggesting that RECK is required for proper wing muscle development in the later stages of development. As described in Section 6.6 and Figure 8, RECK inhibits ADAM10-mediated shedding of Notch ligands and consequently promotes Notch signaling in the mouse. It will be of interest to determine whether RECK is also directly regulated by Notch signaling in mammalian cells (e.g., muscle progenitors, NPCs) to form a feedback loop.

Gutiérrez et al. [147] found transient upregulation of RECK during myogenic differentiation of C2C12 cells. RECK knockdown resulted in reduced Notch signaling, enhanced Myogenin and Myosin expression, and thicker myotubes. They also found that muscle damage in mice transiently upregulated RECK, MT1-MMP, MMP2, and MMP9 expression and that myofiber regeneration was accelerated in *Reck^+/−^* mice with reduced fibrotic ECM accumulation, suggesting an adverse effect of RECK on muscle regeneration. These findings are not necessarily inconsistent with the dual roles of RECK during muscle differentiation discussed above (Figure 10).

Mahl et al. [148] studied the effects of RECK on the migration and differentiation of human mesenchymal stem cells (hMSCs). Their data suggest that RECK suppresses cell migration and promotes osteogenic differentiation in this system. Costa Fernandes and Zambuzzi [85] used the mouse cell line MC3T3 and also found increased RECK expression after osteogenic differentiation.

Although these findings support the idea that RECK is involved in musculoskeletal development, more direct evidence with gene manipulation in vivo is lacking. Given the multiple lines of *Reck*-floxed mice (Figure 4, Table S6) and various gene editing techniques available today, studies to better assess the roles of RECK in these processes are increasingly feasible.

### 6.8. Regulation of Blood Cell Mobilization from Bone Marrow (Table S5(7))

Vagima et al. [149] focused on the role of RECK in blood cell development. Circulating human CD34^+^ cells (hematopoietic stem cells) had higher MT1-MMP and lower RECK compared with bone marrow (BM) cells. Treatment of mice with G-CSF increased MT1-MMP expression and decreased RECK expression in BM cells in a PI3K/Akt-dependent manner. The mobilization of human CD34^+^ cells in chimeric NOD/SCID mice by G-CSF was inhibited by anti-MT1-MMP treatment, while RECK neutralization promoted motility and egress of BM CD34^+^ cells, suggesting that the mobilization of hematopoietic progenitor cells is regulated by the balance between MT1-MMP (positive) and RECK (negative). This model also awaits validation using genetically engineered animals.

### 6.9. Supporting Somatic Growth (Table S5(8))

**Figure 11:**
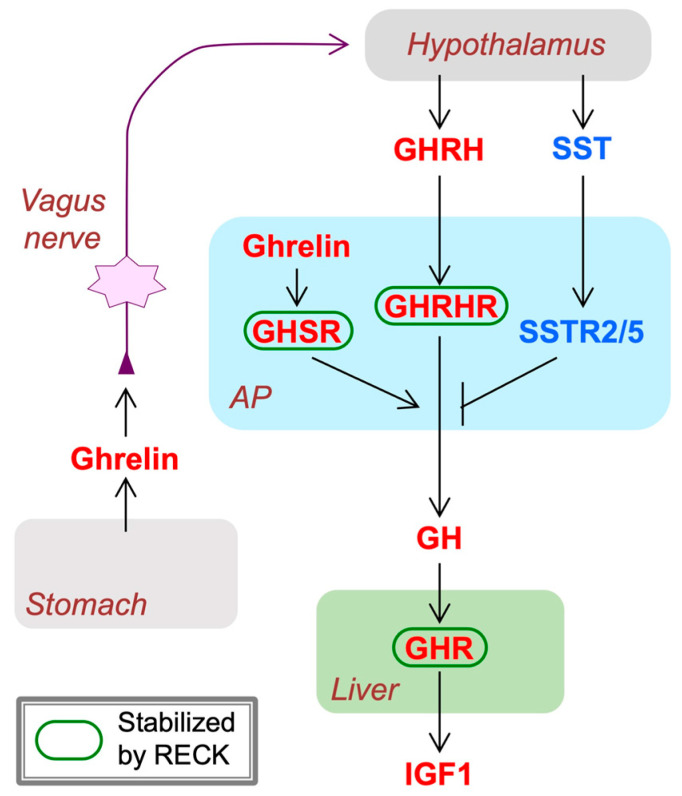
Effects of RECK on the GH/IGF1 axis. Mutant mice expressing RECK at about 20% of the normal level exhibit reduced (~90%) body weight. In the mutant mice, three membrane receptors (green ellipses) involved in the regulation of somatic growth were found to be destabilized. Abbreviations: GHRH, growth-hormone-releasing hormone; SST, somatostatin; GHSR, growth hormone secretagogue receptor; GHRHR, growth-hormone-releasing hormone receptor; SSTR, somatostatin receptor; GH, growth hormone; GHR, growth hormone receptor; IGF1, insulin-like growth factor 1. See Ogawa et al. [88] for more details.

Ogawa et al. [88] found persistent and abundant expression of *Reck* in the anterior pituitary gland (AP) and the arcuate nucleus of the hypothalamus (ARH) in the adult mouse brain. These organs are known to play roles in somatic growth control mediated by growth hormone (GH) and insulin-like growth factor 1 (IGF1): the pathway is termed the GH/IGF1 axis (Figure 11). They also found that two lines of viable *Reck* mutant mice, Reck-Hypo mice (Reck<tm1Noda>/Reck<tm2.2Noda>; Figure 4; see Section 6.4) and mice in which *Reck* was conditionally inactivated around day 10 after birth (using Reck<tm2.1Noda>/Reck<tm3.1(cre/ERT2)Noda> mice, termed iKO; Figure 4), showed smaller-than-normal body size. In both mutant mice, the expression of three receptor proteins involved in the GH/IGF1 axis (GHRHR and GHSR in the AP and GHR in the liver) was increased at the mRNA level but decreased at the protein level, suggesting decreased stability (degradation) of these surface proteins. These results indicate that in mice older than 10 days, RECK is not essential for their survival but contributes to somatic growth by stabilizing multiple components of the GH/IGF1 axis. It is tempting to speculate that since larger animals need stiffer bones and tissues, RECK coordinates both somatic growth at the organismal level and ECM accumulation at the tissue level.

## 7. Altered Expression in Tumors

In the initial RNA blot hybridization experiments, *RECK* mRNA (~4.6 kb) could be detected in a wide variety of normal human organs and in human fibroblasts (MRC5) as well as in a mouse fibroblast-derived cell line (NIH3T3), but not in a human fibrosarcoma cell line (HT1080) or NIH3T3 cells transformed by various oncogenes [7], suggesting that the RECK gene might be sensitive to malignant transformation. It has been found since then that RECK expression indeed tends to be downregulated in many types of cancer cells compared to their normal counterparts (Table 3). In most of these cancers, the extent of RECK downregulation is associated with poorer prognoses. The prevalence of RECK downregulation in various cancers is also evident from several gene expression profile datasets; although, for data with Affymetrix GeneChip arrays, the probe “205407_at” works for human *RECK* mRNA, for unknown reasons, many other probes do not match the *RECK* sequence.

Mechanisms of RECK downregulation in cancer cells is an important issue, since it may provide some clues not only to the mechanism of carcinogenesis but also to novel approaches to cancer diagnoses and prevention. Findings so far indicate that RECK can be downregulated in cancer cells by multiple mechanisms as summarized below.

### 7.1. Reduced Gene Expression

#### 7.1.1. Early Findings

**Figure 12:**
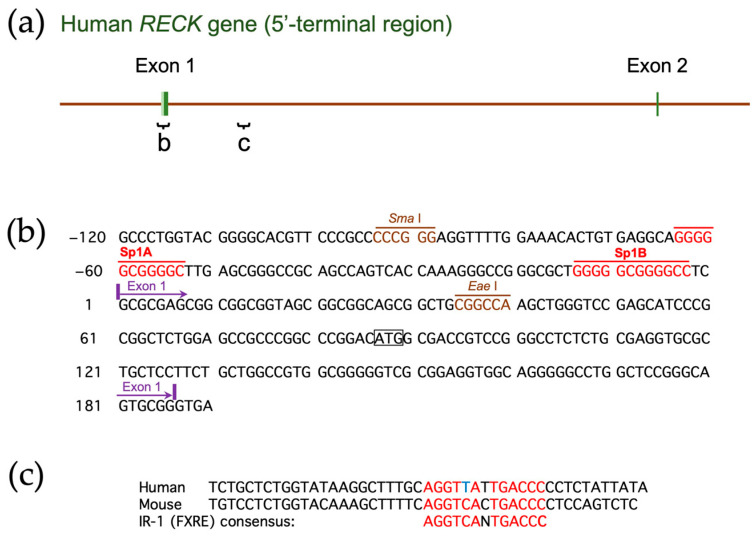
Cis-regulatory elements of the *RECK* gene. (**a**) Relative positions of the area shown in (**b**,**c**). (**b**) Nucleotide sequence around exon 1 of the human *RECK* gene. Sequences in rodents are highly similar in this region. The initiation codon is boxed, and the positions of two restriction sites (Sma1, Eae1), two Sp1 sites (Sp1A, Sp1B), and the beginning and end of exon 1 are indicated. (**c**) Nucleotide sequences around the farnesoid X receptor binding site (FXRE; highlighted in red) in the intron 1 of human and mouse *RECK* gene.

Sasahara et al. [8] reported that the *HRAS^G12V^* oncogene suppressed the expression of RECK in mouse and rat fibroblast cell lines and that two Sp1 sites (Sp1A and Sp1B) in the upstream proximal region of the *Reck* gene (Figure 12b) were involved in this regulation. Hsu et al. [150] reported that the *HER2/neu/ERBB2* oncogene also suppressed *RECK* expression through the proximal Sp1 sites and that this suppression was accompanied by increases in HDAC1 and phospho-Sp1 bound to these sites and an increase in phospho-ERK protein in the cell, suggesting the involvement of activated RAS/ERK signaling pathway, SP1 phosphorylation, and HDAC1 recruitment in this suppression. Through comparison between the copy number alterations (CNAs) and gene expression changes found in three types of tumors (glioblastomas, bladder tumors, and breast tumors), Lee et al. [151] found that the gene set termed the “RECK pathway” was negatively correlated with increased copy number of the p15.4 to p15.5 region of chromosome 11, supporting the idea that increased activity of *HRAS* (residing on chromosome 11p15.5) led to RECK downregulation.

#### 7.1.2. Transcription Factors

Chang et al. [152] reported that overexpression of Sp1 or Sp3 enhanced *Reck* promoter activity in Drosophila SL2 cells and that an oncogenic *HRAS* mutant (*Ha-ras^Val12^*) increased the binding of histone deacetylase 1 (HDAC1) to the Sp1B site (Figure 12b) through ERK signaling. Using a technique called suppression subtractive hybridization PCR (SSH PCR), Yeh et al. [153] identified *RbAp46/RBBP7* as a gene upregulated by oncogenic *Ha-ras^Val12^*. Their data implicate RbAp46 in the recruitment of HDAC1 to the proximal Sp1 sites. Recent data by Yoshida et al. [154] indicate that an HDAC inhibitor, DSK638 (see Section 8.4.2), upregulates RECK through the two proximal Sp1 sites and that DSK638 exerts this activity probably by converting KLF2, a repressor binding at these sites, into an activator.

These findings highlight the importance of the proximal Sp1 sites in *RECK* downregulation associated with *RAS* activation. However, given the wide variety of tumor types exhibiting decreased RECK expression (see Table 3), it is feasible that many other cis-regulatory elements and transcription factors playing roles in tumor-associated RECK downregulation are yet to be discovered.

#### 7.1.3. Epigenetic Control

Ning and Ma [155] reported that knockdown of *enhancer of zeste homolog 2* (*EZH2*) in breast cancer cell lines (Hs-578T, MDA-MB-231) resulted in upregulation of *RECK* mRNA with a concomitant decrease in the level of H3K27me^3^, a repressive histone modification, associated with an upstream region (ca. −1.5 kb) of the *RECK* gene. Their data are consistent with the model that ERK signaling downregulates *RECK* expression by suppressing the AKT-mediated phosphorylation of EZH2 at serine-21, which is known to suppress the methyltransferase activity of EZH2, thereby increasing H3K27me^3^ associated with the *RECK* promoter.

**Figure 13:**
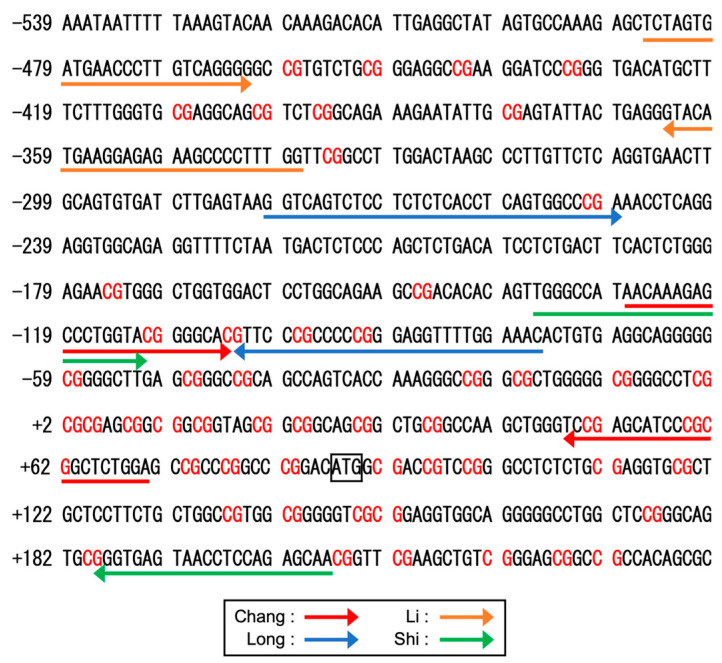
Positions of primers used in methylation studies of the human *RECK* gene. Residues are numbered relative to the transcription start site. The initiation codon is boxed, and CpG dinucleotides are highlighted in red. See Table S8 for primer sequences and literature.

Chang et al. [156] reported that oncogenic Ha-ras^Val12^ upregulates DNMT3b through activation of ERK signaling, thereby inducing *RECK* promoter methylation and consequent suppression of *RECK* transcription in a mouse fibroblast cell line, NIH3T3. The correlation among *RAS* mutations, *RECK* promoter methylation, and RECK downregulation was also confirmed in clinical samples of lung [157] and colon [158] cancer. Subsequent studies revealed that *RECK* silencing by DNA hypermethylation was found at a substantial frequency in a wide variety of cancers (Table S7; for methods, see Table S8 and Figure 13). In the case of breast cancer, Hill et al. [159] performed a genome-wide hypermethylation study on clinical samples and found that hypermethylation of six genes (RECK, SFRP2, UAP1L1, ACADL, ITR, and UGT3A1) was inversely correlated with relapse-free survival. This finding is of particular importance not only because RECK was detected by this unbiased genome-wide screening but also because the results strongly suggest that RECK is a cancer recurrence suppressor.

CpG methylation is an important mechanism for maintaining gene repression which is initially triggered by other mechanisms, such as repressor binding and histone modifications. In the aforementioned study, Chang et al. [156] used NIH3T3 cells to find that DNA demethylation by 5-azacytidine (AZC) alone fully reactivated *RECK* gene expression that was suppressed by the *RAS* oncogene. On the other hand, Shi et al. [160] showed that DNA demethylation with 5-aza-2′-deoxycytidine (5-azadC) was insufficient to reactivate RECK expression in three breast cancer cell lines (MCF7, T47D, and ZR-75-1). Instead, they found that an HDAC inhibitor, MS275, could reactivate RECK expression in one cell line (MCF7) but not the others and that the MS275-responsiveness of RECK expression was better correlated with the absence of methylation in the proximal promoter/exon-1 region (green arrows in Figure 13) than that in the intron 1 region described by Hill et al. [159]. Importantly, RECK expression could be reactivated in T47D and ZR-75-1 cells after treatment with both 5-azadC and MS275, suggesting the potential utility of combination therapy in treating tumors carrying methylation in the proximal promoter/exon-1 region. These results also support the idea that CpG methylation is one of multiple mechanisms by which RECK expression is downregulated in actual cancer cells.

### 7.2. Regulation by Non-Coding RNAs

#### 7.2.1. MicroRNAs

Chan et al. [161] found that microRNA-21 (miR-21) was overexpressed in glioblastoma and that miR-21 knockdown induced apoptosis in glioblastoma cell lines. They subsequently identified *RECK* and *TIMP3* mRNAs as targets of miR-21 [162]. In studying molecular changes induced by embryo implantation in the mouse uterus, Hu et al. [163] found upregulation of miR-21 at the implantation sites, and they also identified *Reck* mRNA as a target of miR-21. Following these two studies, a number of papers describing the role of miR-21 in the regulation of RECK expression (>40 papers) as well as papers describing over 20 groups of microRNAs regulating *RECK* expression (>90 papers) have been published (Table 4). Typically, these studies demonstrated the effects of overexpression and/or knockdown of each microRNA (1) on the expression of endogenous RECK, and (2) on the luciferase expressed from a transfected vector in which a predicted target site of the microRNA or its mutant (negative control) is placed downstream of the luciferase gene. The second assay is designed to test whether the effects on endogenous RECK expression observed in the first assay are due to direct targeting by microRNA.

#### 7.2.2. LncRNAs

Long non-coding RNAs (lncRNAs) are non-coding RNAs longer than 200 bases and exert their bioactivities via various mechanisms. One mechanism is to bind and inhibit microRNAs, which is known as the “sponging” effect. Su et al. [164] took advantage of this activity and engineered an adenoviral vector to express an artificial lncRNA (termed interfering long non-coding RNA or i-lncRNA) designed to target eight groups of microRNAs (miR-17, 19, 20, 21, 125, 146, 155, and 221/222). This virus could upregulate PTEN, p27^kip1^, TIMP3, and RECK, downregulate p38/MAPK, survivin, CDK4, and MYC, and suppress tumor growth in a xenograft model, demonstrating the potential value of such lncRNAs in cancer therapy. Subsequent studies revealed the activities of several endogenous lncRNAs to upregulate RECK (Table S9), probably by inhibiting microRNAs targeting *RECK* mRNA (see Table 4), or to downregulate RECK by recruiting EZH2 to the RECK promoter (see Section 7.1.3). In the pioneering work mentioned above, Su et al. [164] presented data implicating miR-17, 125, and 155 in downregulation of RECK expression, although it has yet to be determined whether these microRNAs directly target *RECK* mRNA.

### 7.3. Altered Protein Stability

Lin et al. [69] reported that the cytosolic phosphoprotein CRMP2 is downregulated in breast cancer cells and that CRMP2 binds and stabilizes RECK (see Section 3.2). In addition, Zhou et al. [165] reported that the E3 ubiquitin ligase SIAH1 is frequently upregulated in gastric cancer and that SIAH1 promotes migration and invasion of cancer cells by triggering proteasome-mediated degradation of the RECK protein. Although these models are conceptually intriguing, both CRMP2 and SIAH1 are cytosolic proteins and in different subcellular compartments from RECK as discussed in Section 3.2.

### 7.4. Involvement of Other Signaling Pathways

Wei et al. [166] reported that double knockdown of STAT3 and p27 in gastric cancer cell lines results in reduced invasive activity, decreased SP1 expression, and increased RECK expression. A cause–effect relationship between SP1 and RECK in this system remains to be established since previous studies [150,152] indicated that the amount of SP1 in the cell is not simply correlated with *RECK* promoter activity and that histone deacetylation and SP1 phosphorylation play important roles in oncogene-induced *RECK* repression. Nevertheless, the finding by Wei et al. [166] that STAT3 suppresses RECK expression and the finding by Walsh et al. [64] that RECK suppresses STAT3 activation (see Section 3.2 and Section 9.2) raise the interesting possibility that RECK and STAT3 form a negative feedback loop. Since multiple candidate STAT3 consensus sequences (CACGTG) are found in the upstream region of *RECK* gene, it would be interesting to test whether any of these sites, rather than the Sp1 sites, are directly involved in this regulation.

As discussed in Section 6, *Reck* may be activated by Notch signaling in Drosophila muscle progenitor cells [146] while RECK supports Notch signaling in mouse neural precursor cells [81]. It is presently unknown whether these mechanisms operate in a tissue-specific manner, and research has focused only on animal development so far. In carcinogenesis, however, Notch signaling is known to play dual roles [167], and hence, it would be worth investigating whether there is any interplay between RECK and Notch during carcinogenesis.

## 8. Stimuli and Substances That Affect RECK Expression

Early findings that RECK was downregulated in a wide variety of cancer cells (see Section 7) motivated researchers to find stimuli that influence RECK expression in various cells. Such knowledge may help us understand the nature of malignancy and develop novel approaches to cancer therapy and prevention. Here we attempt to categorize and summarize the findings in this area.

### 8.1. Physical Stimuli and Environmental Conditions (Table S10)

RECK could be upregulated after the cells were exposed to electromagnetic waves such as non-ablative laser [168] and gamma rays [169]. In the case of gamma rays, TGFβ-signaling was implicated in the phenomenon. A photodynamic therapy, known to promote production of reactive oxygen species, was also found to induce RECK expression [170]. In contrast to gamma ray, X-rays were found to downregulate RECK expression [171]. Given these intriguing data, it would be interesting to test the effects of electromagnetic waves with a greater variety of wave lengths and doses on RECK expression.

Hatta et al. [172] found that the level of RECK expression in mouse embryo fibroblasts was cell density-dependent. Their results with protein kinase inhibitors implicated SRC and FAK in this regulation. Whether RECK expression is also cell density-dependent in other types of cells is presently unknown; however, in assessing the level of RECK expression in cultured cells, the possibility of its density dependence needs to be kept in mind.

*RECK* expression is also influenced by ambient oxygen concentration. Using cultured cell lines (i.e., HEK293, HRAS-transformed MCF10A, and HT1080), Lee et al. [173] and Jeon et al. [174] found that hypoxia downregulates *RECK* expression in an HDAC1- and HIF1α-dependent manner and that a reverse hypoxia response element (HRE) in the upstream region of *RECK* (−2345 to −2333) is involved in this repression. In a later study, Jeon et al. also found that this effect requires three MAP kinases (ERK, JNK, and p38) [175]. Loayza-Puch et al. [176], on the other hand, reported that RECK is downregulated by hypoxia as well as a hypoxia mimetic agent, deferoxamine, in a colon cancer cell line (SW620); in that system, RECK downregulation was attributed to the *RECK*-targeting microRNAs miR-372/373 that were upregulated by TWIST1 in response to HIF1α stabilization. Zhang et al. [177,178] reported that another hypoxia mimetic agent, CoCl_2_, downregulated RECK in cancer-derived cell lines (786-0, HepG2, SiHa) whereas it upregulated RECK in non-malignant cell lines (HK-2, HMEC-1, and HL-7702). Even though the identity of HL-7702 has recently been challenged [179], whether contrasting responses of RECK to hypoxia between normal and cancer cells can be found in other systems is an interesting issue. Ferrigno et al. [180] observed downregulation of RECK with concomitant activation of three MAP kinases after transient (1 h) ischemia and reperfusion (1 h) in the rat liver. This indicates that hypoxia downregulates RECK in normal tissues, at least in the liver. Hypoxia may affect RECK expression also in a non-cell-autonomous fashion. Ren et al. [181] reported that extracellular vesicles secreted by bone marrow-derived mesenchymal stem cells subjected to hypoxia could downregulate RECK in lung cancer cell lines (A549, H23), probably by extracellular vesicle delivery of *RECK*-targeting microRNA, miR-21 (see Section 7.2.1).

### 8.2. Microbial Infection (Table S11)

Liu et al. [182] reported that in a human nasopharyngeal carcinoma cell line, latent membrane protein 1 (LMP1), a metastasis-promoting protein encoded by Epstein–Barr virus, repressed *RECK* gene by activating ERK signaling, and ERK signaling in turn repressed RECK expression in a manner dependent on the proximal SP1B site in the RECK promoter (Figure 12a,b).

Subsequently, two other viruses were found to upregulate RECK expression: canine distemper virus in a canine macrophage/monocytic tumor cell line and Newcastle disease virus (strain D90) in a human oral squamous cell carcinoma cell line (Table S11). Mechanisms of RECK induction by these viruses remain unknown.

Qin et al. reported [183] that the bacteria *Mycoplasma pneumoniae* induced MMP9 secretion from bronchial epithelial cells with a concomitant increase in phospho-SP1 and a decrease in RECK, which would promote inflammatory responses.

Persistent infection with high-risk strains of human papilloma virus (HPV) is a risk factor of cervical cancer, and the HPV oncoproteins, E6 and E7, are known to bind and inactivate the major tumor suppressor proteins p53 and RB, respectively. In an attempt to find additional effects of these oncoproteins during cervical carcinogenesis, Cardeal et al. [184] co-expressed E6 and E7 of HPV16, a high-risk strain, in human foreskin keratinocytes and found that RECK was downregulated. Thus, RECK is a target not only of several cellular oncoproteins but also of DNA tumor virus oncoproteins.

### 8.3. Polypeptides

#### 8.3.1. Secreted Polypeptides (Table 5(1–3))

Two peptide hormones were reported to suppress RECK expression: vasoactive intestinal peptide (VIP) in human prostate cancer cell lines [185] and angiotensin II in mouse cardiac fibroblasts [108]. In the case of angiotensin II, a signaling cascade involving NOX4, ERK, and SP1 was implicated.

The activities of four growth factors and four cytokines to modulate RECK expression via diverse mechanisms have been described. In pancreatic stellate cells, TGFβ upregulates RECK by protecting RECK from aspartic protease(s)-mediated degradation [186]. In human osteoarthritic chondrocytes, IGF1 upregulates, and IL-1 and TNFα downregulate *RECK* mRNA [187]. In mouse cardiac fibroblasts, IL-18 downregulates RECK protein in an SP1-dependent manner [188], suggesting regulation at the transcriptional level. In human aortic smooth muscle cells, PDGF-BB-mediated downregulation of RECK was attributed to the upregulation of two microRNAs (miR-221 and miR-222) that target *RECK* mRNA [189]. Although the mechanisms remain unknown, VEGF and IL-32a upregulate RECK in vascular cells (Table 5(2,3)).

**Table 5:** Peptides and proteins affecting RECK expression.

Category	Molecule	System	Effects on RECK *	First Report(s)		
				First Author	Year	PMID
**1. Peptide hormone**	vasoactive intestinal peptide	human prostate cancer cell lines (LNCaP, PC3)	D	Fernández- Martínez	2009	19189304
	angiotensin II	mouse cardiac fibroblasts	D	Siddesha	2013	24095877
**2. Growth factor**	TGFβ1	rat pancreatic stellate cells	U	Lee	2008	18300271
	IGF1	human osteoarthritic chondrocytes	U	Kimura	2010	20395433
	VEGF	human microvascular endothelial cell line (HMEC1)	U	Clark	2011	21287525
	PDGF-BB	human aortic smooth muscle cells	D	Higashi	2019	30716386
**3. Cytokine**	IL-1, TNFα	human osteoarthritic chondrocytes	D	Kimura	2010	20395433
	IL-18	mouse cardiac fibroblasts	D	Siddesha	2014	24265116
	IL-32α	mouse left common carotid artery	U	Son	2017	28740544
**4. Protease regulator**	TIMP2	human microvascular endothelial cells	U	Oh	2006	16491114
	TIMP1	xenopus laevis embryo	D	Nieuwesteeg	2014	24616631
	TMPRSS4	human hepatocellular carcinoma cell lines (BEL-7402, MHCC97L)	D	Wang	2015	26190376
**5. E3 ubiquitin ligase**	SKP2	human gastric carcinoma cell line (MGC803)	D	Wei	2013	23333463
	TRAF3IP2	aorta of Apoe^−/−^ mice	U	Sakamuri	2016	27237075
	EMI1/FBOX5	human breast cancer cell lines (MDA-MB-231, SUM149PT)	U	Kuang	2023	38041032
**6. C-type lectin**	HIP/PAP/REG3	humman pancreatic stellate cells	D	Li	2009	19077460
	CLEC19A	human and rat glioma cell lines (U87, C6)	U	Mohajerani	2024	38167030
**7. Gene expression regulator**	MyoD	mouse fibroblast cell line (C3H10T1/2)	D	Echizenya	2005	16007210
	MRF4		U			
	FXR	liiver of Fxr^−/−^ mice	U	Peng	2014	24291500
	MCPIP1	human clear cell renal cell carcinoma cell lines (Caki-1, Caki-2)	U	Gorka	2020	32971087
**8. Proteasome component**	ADRM1	human ovarian cancer cell line (OAW42)	D	Fejzo	2011	21432940
**9. Cytoskeletal regulator**	PHACTR1	Human umbilical vein endothelial cells (HUVECs)	D	Jarray	2015	26362351
**10. Chaperon**	mortalin/GRP75/ HSPA9	human hepatocellular carcinoma cell lines (HepG2, HCCLM3)	D	Teng	2021	34876128
**11. Lipoprotein**	oxidatively modified LDL	human aortic smooth muscle cells	D	Chandrasekar	2023	37830075
**12. Neuronal protein**	CRMP2/DPYSL2	human cell lines (MDA-MB231, HEK293T)	U	Lin	2020	32778769
**13. Metabolic enzyme**	NQO1	human cervical cancer cell lines (SiHa, CaSki)	D	Wattanathavorn	2024	39733409

* U: upregulation; D: downregulation.

#### 8.3.2. Protease Regulators (Table 5(4))

Oh et al. [190] found that treatment with TIMP2 upregulated RECK in the mouse fibroblast cell line NIH3T3. They suggested the involvement of SRC inactivation downstream of integrin signaling in this phenomenon. Nieuwesteeg et al. [191] found that microinjection of TIMP1 mRNA into Xenopus embryos at the one-cell stage resulted in downregulation of *RECK* mRNA at stage 30 (~1.5 days embryo). Although the mechanism of this downregulation remains unknown, their experiments with *TIMP1* mRNA fragments demonstrated that its C-terminal portion was sufficient for this effect.

Wang et al. [192] found that TMPRSS4, a pro-uPA-activating enzyme upregulated in various cancers, downregulated RECK and induced EMT when overexpressed in human hepatocellular carcinoma cell lines. These effects were suppressed by an MEK inhibitor, U0126, suggesting the involvement of ERK signaling. The serine protease uPA (urokinase-type plasminogen activator) converts plasminogen into plasmin, another serine protease involved in thrombolysis as well as maturation of several molecules (e.g., MMPs, TGFβ), affecting ECM remodeling, angiogenesis, and inflammation; uPA itself promotes tumor invasion and metastasis [193]. How TMPRSS4 activates ERK and to what extent RECK downregulation contributes to the biological effects of TMPRSS4 remain unknown.

#### 8.3.3. E3 Ubiquitin Ligases (Table 5(5))

Yoshida et al. [117] reported that acute RECK overexpression downregulated *SKP2* (an E3 ubiquitin ligase gene) mRNA in a human colon cancer cell line (see Section 6.6). Interestingly, Wei et al. [166] reported that knockdown of SKP2 resulted in upregulation of RECK protein in a gastric cancer cell line, suggesting a negative feedback loop between RECK and SKP2. Two other E3 ubiquitin ligases have also been implicated in upregulation of RECK. First, the level of *Reck* mRNA was markedly reduced in the aorta of male *Traf3ip2*^−/−^; *Apoe*^−/−^ double knockout mice as compared to those of male *Apoe^−/−^* mice (no significant difference in female mice) [194]. Second, *RECK* mRNA was decreased when EMI1 was knocked down in two breast cancer cell lines [195]. This finding is counterintuitive since EMI1 is oncogenic and RECK is a tumor suppressor. In all these cases, mechanisms of altered gene/protein expression remain unknown.

#### 8.3.4. C-Type Lectins

C-type lectins are characterized by their calcium-dependent carbohydrate-binding domains; some members of this family play roles in cell–cell adhesion, immune response to pathogens, and apoptosis. Two less-studied family members, HIP/PAP/REG3 and CLEC19A, modulate RECK expression (Table 5(6)). These findings suggest that RECK may be a target (or effector) of these C-type lectins, but the mechanisms through which they modulate RECK expression remain unknown.

#### 8.3.5. Gene Expression Regulators (Table 5(7))

*Reck* promoter activity could be modulated by myogenic transcription factors in C3H10T1/2 cells (see Section 6.7) [79]; MyoD-mediated repression involves the Sp1B site in the *Reck* promoter (Figure 12b).

Another transcription factor implicated in *RECK* gene regulation is the farnesoid X receptor (FXR; also known as NR1H1), a member of the ligand-activated nuclear receptor superfamily. FXR heterodimerizes with retinoid X receptor alpha (RXR-alpha, also known as NR2B1) and binds to specific DNA response elements (FXREs) such as an inverted repeat of the canonical AGGTCA half-site interspaced by one nucleotide (Figure 12c; the element termed IR-1) [196,197]. Peng et al. [198] reported that an IR-1 site found in the first intron of *Reck* (Figure 12c) is involved in this regulation.

Gorka et al. [199] reported that *RECK* mRNA is upregulated by the anti-inflammatory protein Monocyte Chemoattractant Protein-Induced Protein 1 (MCPIP1), also known as Regnase-1 and ZC3H12A, in renal cell carcinoma cell lines. Since Regnase-1 is an endoribonuclease known to degrade multiple mRNAs involved in immune cell activation, it is likely that Regnase-1 degrades mRNA(s), encoding certain protein(s) that can downregulate RECK.

#### 8.3.6. Miscellaneous Proteins (Table 5(8–13))

The chromosomal region 20q13 is frequently amplified in ovarian cancers. Fejzo et al. [200] identified the Adhesion-Regulating Molecule 1 (*ADRM1*) gene as a critical target located in this region and found that knockdown of ADRM1 resulted in upregulation of *RECK* mRNA in an ovarian cancer cell line. ADRM1 is a proteasomal component acting as a ubiquitin receptor to recruit a deubiquitinating enzyme, ubiquitin carboxyl-terminal hydrolase L5 (UCHL5), and hence ADRM1 is expected to affect the degradation of a wide range of proteins.

Associations between certain small nucleotide polymorphisms (SNPs) in the phosphatase and actin regulatory protein (PHACTR1) locus at chromosome 6p24.1 and multiple cardiovascular diseases have been reported. Jarray et al. [201] found, using a proteome profiler array, that knockdown of PHACTR1 in HUVEC upregulated RECK, TIMP1, and TIMP2.

A mitochondrial chaperon, Mortalin (also known as GRP75 and HSPA9), has been characterized as a protein overexpressed in multiple cancers. Teng et al. [202] found that mortalin overexpression resulted in RECK downregulation and mortalin knockdown resulted in RECK upregulation in hepatoma cell lines. In these three cases, the studies shed some light on the functions of the molecules of interest but provide little information on the mechanisms through which RECK expression is modulated.

NQO1 is an enzyme known to be highly expressed in various solid tumors and to support their malignant phenotype by reducing oxidative stress and modulating various signaling pathways. Wattanathavorn et al. [203] reported that knockdown of NAD(P)H quinone oxidoreductase 1 (NQO1) increased the level of RECK protein in cervical cancer cell lines, suggesting that RECK may be downregulated by oxidative stress. This model is consistent with the effects of H_2_O_2_ to reduce RECK expression (see Section 8.4.8) and several antioxidants to increase RECK expression (see Section 8.4.3).

### 8.4. Small Molecules

Small molecules that modulate RECK expression are of potential importance: the inducers may be useful for cancer therapy/prevention while the suppressors may increase the risk of cancer and other disorders in which RECK downregulation plays a role.

#### 8.4.1. NSAIDs (Table 6(1))

Nonsteroidal anti-inflammatory drugs (NSAIDs) exert antiangiogenic and anti-metastatic activity and suppress MMP activity. Liu et al. [204] found that two NSAIDs (NS398 and aspirin) upregulated RECK expression in a human lung cancer cell line (CL-1). Overexpression of cyclooxygenase-2 (COX-2) or treatment with prostaglandin E2 (PGE2) did not affect RECK expression, suggesting that this activity was COX-2-independent. On the other hand, Zhou et al. [205] reported that a COX-2-inhibiting NSAID (celecoxib) also upregulated RECK expression in an osteosarcoma cell line (MG-63). Hence, different NSAIDs upregulate RECK, although the mechanism of induction might vary among drugs and/or cell types.

**Table 6:** Small molecules affecting RECK expression.

Category	Compound	First Report(s)		
		First Author	Year	PMID
**Inducers**				
**1. NSAID**	NS398, aspirin salicylic acid	Liu	2002	12447698
		Siddesha	2014	24265116
	celecoxib	Zhou	2015	26592832
**2. HDAC inhibitor**	trichostatin A	Liu	2003	12810630
		Somanna	2016	27278287
	apicidin	Ahn	2012	22781396
	valproic acid	Chen	2012	22528797
	MS275	Shi	2016	27058625
	DSK638, JNJ-26482585, MS275, CI-994	Yoshida	2022	35149728
**3. Anti-DNA-methylation**	5-azacytidine	Chang	2006	16951151
**4. Flavonoid, polyphenol, antioxidant**	epigallocatechin-3-gallate	Kato	2008	18665171
		Chang	2014	25184134
		Zhou	2015	26299812
	black tea polyphenols	Murugan	2009	19528495
	eugenol	Manikandan	2010	20434464
	ellagic acid	Huang	2011	21573219
	RY10-4	Xue	2014	24300195
	icariin	Li	2015	25845681
	casticin	Yang	2017	28352361
	empagliflozin	Das	2020	31862399
	salvianolic acid B	Teng	2021	34876128
**5. Alcohol deterrent**	disulfiram	Murai	2010	21304177
**6. Anticancer drug**	Doxorubicin *, camptothecin, daunorubicin *, mechlorethamine *, mitoxantrone *, diaziquone, methotrexate *, paclitaxel *, raloxifene *, etoposide *	Murai	2010	21304177
	p-dodecylaminophenol	Takahashi	2013	23953690
	gambogic acid	Qi	2015	24532189
	vemurafenib	Sandri	2016	27436149
	LQB-118, paclitaxel	Martino	2023	36585169
**7. Steroid, triterpenoid**	raloxifene	Murai	2010	21304177
	estradiol-17β (E2) **	Zhang	2012	22302680
		Barneze Costa	2020	32911016
	tomatidine	Yan	2013	23566884
	raddeanin A	Xue	2013	23988447
	α-solanine	Shen	2014	25116803
	simvastatin	Gallelli	2014	25432084
	JSI-124 (cucurbitacin I)	Zhang	2015	25571964
	solasodine	Shen	2017	28283413
	nimbolide	Kowshik	2017	28515436
**8. Antipathogen**	pyrithione, thimerosal, gramicidin, haloprogin, albendazole, meclocycline, demeclocycline, minocycline, pyrimethamine, cycloheximide, hycanthone, doxycycline	Murai	2010	21304177
	β-asarone	Wu	2015	26502896
	anacardic acid	Nambiar	2016	27737732
	dihydroartemisinin	Shao	2017	28208619
	minocycline	Higashi	2019	30716386
**9. Natural dye**	alkannin	Mao	2019	31349748
	curcumin (EF24)	Jia	2019	30841433
		Zhou	2020	32081769
		Higashi	2024	39451191
**10. Alkaloid**	cephaeline, emetine, lycorine, harmine	Murai	2010	21304177
	harmine	Shen	2018	29510387
	sinomenine	Shen	2020	32349289
**11. Dietary substance, metabolic product**	menadione (vitamin K3)	Murai	2010	21304177
	docosahexaenoic acid	Siddesha	2014	24447911
**12. Others**	podophyllotoxin, trimeprazine, perhexiline, triamterene, triflupromazine, piperlongumine	Murai	2010	21304177
**13. Plant extract**	*Davallia bilabiata* (fern, GuSuiBu)	Liu	2017	27993633
	*Annona muricata* (soursop)	Drishya	2020	32661216
	Ruyan Neixiao Cream	Lin	2022	35094593
**Suppressors**				
**14. Dietary substance and metabolite**	ethanol	Yamamoto	2012	23213437
		Kisby	2021	34573170
	H_2_O_2_	Gallelli	2014	25432084
	glucose	Das	2020	31862399
**15. Industrial pollutant**	CdCl2	Yamamoto	2012	23213437
	TCDD, BDE-209	Oliveira Ribeiro	2022	36100121 38097007
**16. Flavonoid**	eupatilin	Fei	2019	31213900
**17. Alkaloid**	emetine	Kim	2015	26332055
**18. Steroid**	27-hydroxycholesterol	Shen	2020	31933392
**19. Crude extract**	*Drynaria fortunei* (fern, GuSuiBu)	Huang	2018	30298000
	cigarette smoke extract	Wang	2024	38387446

* FDA-approved oncology drug. ** Dose-dependent.

#### 8.4.2. HDAC Inhibitors (Table 6(2))

In general, histone deacetylation results in transcriptional repression, and hence histone deacetylase (HDAC) inhibitors are expected to activate gene transcription. Some HDAC inhibitors are also known to exert anti-metastatic and antiangiogenic activities. Liu et al. [206] found that an HDAC inhibitor, trichostatin A (TSA), could upregulate RECK expression. Subsequent studies uncovered the ability of other HDAC inhibitors to upregulate RECK (Table 6). These findings are consistent with the model proposed by Chang et al. in 2004 [152] that the recruitment of HDAC1 to the proximal Sp1 sites (Figure 12b) is involved in *RECK* gene repression after malignant transformation (see Section 7.1.2).

Through an unbiased screening of chemicals inducing *RECK* expression in fibrosarcoma cells, Yoshida et al. [154] found that an HDAC inhibitor, DSK638, was a potent RECK-inducing and metastasis-suppressing agent. Although some other compounds (e.g., JNJ-26482585, MS275, CI-994) sharing the same skeletal structure with DSK638 (i.e., benzamide) also induce RECK expression, they could not suppress, or could even promote, tumor metastasis in vivo. These results indicate that RECK-inducing activity is a good criterion for initial screening of anticancer/anti-metastatic drugs but is not sufficient for predicting their therapeutic/preventive efficacy in vivo. Based on their data using DSK638 as a positive control and JNJ-26482585 and MS275 as negative controls, Yoshida et al. proposed that the capabilities of compounds to induce (1) RECK expression and (2) cell–cell adhesion in suspension culture in vitro could be useful in finding compounds capable of suppressing metastasis in vivo.

In addition to these small molecules, some fullerene nanoparticles were found to inhibit or downregulate HDAC(s) and induce RECK expression [207,208].

#### 8.4.3. DNA-Methylation Inhibitors, Flavonoids, Polyphenols, and Antioxidants (Table 6(3,4))

Chang et al. [156] reported that 5′-azacytidine upregulated *RECK* in a lung cancer cell line carrying a mutant *KRAS* gene (see Section 7.1.3). Kato et al. [209] reported that epigallocatechin-3-gallate (EGCG) reduced *RECK* gene methylation and induced *RECK* expression in oral and cervical cancer cell lines. EGCG is a major flavonoid polyphenol of green tea that, after methylation by catechol-O-methyltransferase, inhibits DNA methyltransferase (DNMT). In subsequent studies, several flavonoids and (poly)phenolic compounds were found to induce RECK expression (Table 6(4)). Some of these compounds are known to act as antioxidants. Whether these compounds induce RECK expression through inhibition of DNA methylation, regulation of hyper-oxidation, or other mechanisms remains to be clarified.

#### 8.4.4. Anticancer Drugs (Table 6(5,6))

Since RECK is expressed in a wide variety of normal tissues and downregulated in cancer cells, RECK could be a good marker of “normalcy” useful for detecting compounds capable of normalizing cancer cells. Murai et al. [210] tested this idea by using a cell line harboring a reporter gene (*SEAP*) under the control of the *RECK* promoter to screen a small library of 880 bioactive compounds. Among the 34 small molecules found to enhance *RECK* promoter activity (>2-fold), the top-ranking compound (18-fold enhancement) was an alcohol deterrent, disulfiram. The anti-neoplastic activity of disulfiram has been a focus of a substantial number of studies which led to the proposal of its drug-repositioning [211]; however, the role and significance of RECK in its action has never been explored.

It is remarkable that among the 34 small molecules detected by Murai et al. (see above), eight were “oncology drugs” approved by the US Food and Drug Administration (FDA) (asterisks in Table 6(6)). Since the initial library contained 18 such compounds (18/880 = 0.0205), the enrichment after screening (8/34 = 0.235) was more than 11-fold and hence unlikely to represent mere coincidence; it is more likely that these classical anticancer drugs share the common, previously unappreciated, capability to induce RECK expression. Two other compounds detected in this screen and several other compounds found in subsequent studies are also classified as anticancer drugs (Table 6(6)). The mechanisms by which these compounds induce RECK expression remain largely unknown.

#### 8.4.5. Steroids/Triterpenoids (Table 6(7))

In 2010, Peters et al. [212] reported that surgical castration (removal of testicles) in male rats resulted in upregulation of RECK in stromal cells in the ventral prostate. A simple interpretation is that the loss of testosterone triggers this effect. Interestingly, the RECK-inducer screen by Murai et al. [210] detected raloxifene. Raloxifene is structurally distinct from steroids but capable of binding the estrogen receptor and inhibiting its function in some tissues (e.g., breast tissues) while activating its function in others (e.g., bone). Subsequent studies detected several steroid hormones as well as other compounds containing steroid (or triterpenoid) structures that are capable of modulating RECK expression (Table 6(7,18)).

Estradiol-17-beta (also known as E2) is the most potent estrogen produced by the ovary. Barneze Costa et al. [213] reported that E2 at physiological concentrations (10^−8^ M) downregulated *RECK* mRNA in mesenchymal stem cells, whereas E2 at a lower concentration (10^−9^ M) upregulated *RECK* mRNA in these cells. This finding underscores the importance of performing dose–response experiments to fully appreciate the effects of various agents on RECK expression (or any responses for that matter).

Notably, four compounds in this category (alpha-solanine, JSI-124, solasodine, and nimbolide) were reported to downregulate miR-21 (an microRNA targeting RECK). Whether other members of this category affect the expression of miR-21 (or any other microRNA targeting RECK) is therefore an important question.

#### 8.4.6. Antipathogens (Table 6(8))

The *Reck*-inducer screen by Murai et al. [210] also detected a number of drugs against various pathogens, such as bacteria, fungi, and parasites. Subsequent papers reported a few other compounds in this category as RECK inducers. For instance, Higashi et al. [189] demonstrated that minocycline induced endogenous RECK expression in a dose-dependent manner in normal human aortic smooth muscle cells. They also demonstrated that minocycline serves as an antioxidant to suppress the expression of two *RECK*-targeting microRNAs, miR-221 and miR-222. Whether such mechanisms are involved in the actions of other RECK-inducing antioxidants (see Section 8.4.3) is an interesting question.

#### 8.4.7. Miscellaneous RECK Inducers

The *Reck*-inducer screen by Murai et al. [210] detected yet other categories of compounds including several alkaloids, a dietary substance like menadione (vitamin K3), and several other drugs (Table 6(10–12)). Natural dyes, such as alkannin and curcumin, were reported to modulate *RECK* expression (Table 6(9)). Some herbal medicines, such as *Davallia bilabiata* extract (a GuSuiBu formula), *Annona muricata* (soursop), and Ruyan Neixiao Cream were reported to induce *RECK* expression, although their active components have yet to be identified. Of note, *Drynaria fortunei* extract, another formula of GuSuiBu, was found to downregulate RECK (Table 6(19)), illustrating the complexity of traditional remedies.

Nevertheless, given the considerable number and variety of reported *RECK*-inducing small molecules, comparisons of their structures, chemical properties, and the biological processes that they affect are expected to provide some clues to mechanisms underlying *RECK* upregulation.

#### 8.4.8. Substances Suppressing RECK Expression (Table 6(14–19))

Yamamoto et al. reported that RECK protein was downregulated when a chondrogenic cell line (ATDC5) was treated with 0.5 M ethanol (a dietary substance) or 10 mM CdCl_2_ (an environmental pollutant) [123]. Kisby et al. [214] reported that *Reck* mRNA was downregulated in the central nucleus of the amygdala in rats subjected to chronic intermittent exposures to ethanol vapor, a model of alcohol dependence. The mechanisms of RECK downregulation in these systems as well as the effects of ethanol on RECK expression in other tissues need to be explored.

Gallelli et al. [215] reported that one effect of H_2_O_2_ (oxidant) was to downregulate RECK in a lung cancer cell line. This supports the idea that the aforementioned flavonoids and polyphenols (Table 6(4)) might upregulate RECK through their antioxidant activity (see Section 8.4.3).

Das et al. [216] reported that when a human kidney cell line (HK-2), maintained in serum-free medium containing 5.6 mM glucose for 16 hr, was placed in serum-free medium containing 25 mM glucose, RECK protein was markedly downregulated after 12 h but returned to the control levels at 48 h. The authors attributed this effect to concomitant upregulation of miR-21 which could be reversed by Empagliflozin, a selective sodium glucose cotransporter-2 (SGLT-2) inhibitor. The findings could be of clinical importance in terms of diabetes control.

Regarding environmental pollutants, Silva Filho et al. [217] reported the ability of two persistent organic pollutants, 2,3,7,8-tetrachlorodibenzo-para-dioxin (TCDD) and decabromodiphenyl ether (BDE-209), to downregulate *Reck* mRNA in a mouse melanoma cell line. Since RECK downregulation may be causally involved in cancer and several other disorders (see Section 7 and Section 10), environmental pollutants could be important risk factors for such disorders. Since our current knowledge in this area is quite limited (Table 6(15)), more systematic surveys of RECK-affecting pollutants are needed.

The effects of a flavonoid (eupatilin), alkaloid (emetine), and steroid (27-hydroxycholesterol) in downregulating RECK have also been reported, which is of interest in terms of structure–activity relationship, for several members of each group are known to upregulate RECK.

#### 8.4.9. Chemical Carcinogens

Finally, RECK downregulation has been noted in several chemical carcinogenesis studies with animals (Table S12). In all these cases, however, it is unclear whether RECK was downregulated as a primary effect of the carcinogen or as a consequence of cell transformation (see Section 7).

## 9. Mechanisms of Tumor Suppression

Early studies implicated RECK in suppression of tumor angiogenesis, invasion, and metastasis but not in tumor growth [7]. Later studies, however, demonstrated the capability of RECK to suppress tumor growth and began to unveil multiple mechanisms of tumor suppression by RECK. Here, we summarize our current knowledge of this aspect of RECK activity.

### 9.1. Suppression of ECM Degradation, Cell Migration, and Invasion

Two matrix metalloproteinases, MMP2 and MMP9 (also known as gelatinases GELA and GELB, respectively), were initially characterized as enzymes secreted by metastatic tumor cells and capable of digesting type IV collagen, a major component of the basement membrane [218]. RECK was found, in the initial study, to inhibit MMP9 in vitro and to suppress Matrigel invasion and metastasis when overexpressed in metastatic tumor cell lines [7]. Subsequent studies revealed that RECK regulated several metalloproteases (Table 1) and suppressed migration, invasion, and metastasis of various types of tumor cells (Table 2). These findings support the model that RECK suppresses tumor invasion and metastasis by inhibiting ECM-degrading enzymes.

As discussed in Section 6.2, RECK is essential for directional migration of normal cells, and in that case, the ability of RECK to protect ECM receptors (i.e., integrins that are central components of focal adhesions) [49,91,104] may also be important. Focal adhesions (FAs) are molecular complexes that function to anchor actin stress fibers to the ECM and to sense the tensile force between the cell and substrate, which is known to be required for the growth of FAs [219]. Fibronectin links cell surface integrin to collagen fibers, the major component of the ECM. The findings so far suggest that RECK stabilizes FAs and anterior–posterior cell polarity by protecting integrins as well as fibronectin, thereby enabling directionally persistent cell migration. This model may explain, at least in part, the deregulated (or selfish) behavior of tumor cells in which RECK expression is downregulated.

RECK is relatively abundant in mesenchymal cells in normal mouse embryos (see Section 5). In contrast, RECK expression is lower in tumor cells with higher malignancy (see Section 7) which often show a mesenchymal phenotype due to epithelial–mesenchymal transition (EMT). How can we reconcile these paradoxical trends of RECK expression between normal animals and tumor tissues? To address this question, Yuki et al. [91] induced EMT in a non-tumorigenic human epithelial cell line, MCF10A, with TGFβ and found upregulation of RECK with concomitant downregulation of E-cadherin, a hallmark of EMT. In multiple cancer-derived cell lines, however, induction of EMT (namely, E-cadherin downregulation) was not accompanied by RECK upregulation. When RECK expression was reconstituted in a lung cancer cell line, migration speed was decreased while directional persistence was increased on fibronectin-coated dishes after TGFβ-induced EMT, with concomitant upregulation of a fibronectin receptor component, integrin-α_5_. These findings support the model that in cancer cells, EMT is uncoupled from RECK expression, leading to their aberrant behavior.

### 9.2. Suppression of Tumor Angiogenesis

When fibrosarcoma cells artificially overexpressing RECK were subcutaneously inoculated into nude mice, a prominent histological feature of the resulting tumors was reduced tumor angiogenesis as compared to the control tumors (with low RECK expression) [13]. Hanahan and colleagues previously proposed that the mobilization of VEGF by MMP9 produced by macrophages [220] or neutrophiles [221] turns the angiogenic switch ON during carcinogenesis. In addition, degradation of the ECM by MT1-MMP [222] or MMP25 [137] expressed by endothelial tip cells was reported to be essential for angiogenesis. A feasible model to explain the above finding with fibrosarcoma cells would be that RECK antagonized these MMPs to suppress tumor angiogenesis.

Walsh et al. [64] found that the metastatic potential of human breast cancer cell lines was decreased by RECK overexpression and increased by RECK knockdown (Table 2). They also found that RECK could associate with multiple cell surface receptors (IL-8, β1-integrin, galectin-1, IL-6RA, gp130, uPAR; see Section 3.2), suppress cytokine signaling (IL6, 10, 8, VEGF, HIF1-alpha), decrease phospho-STAT3, and downregulate uPA and VEGF production. Based on these findings, they proposed that RECK suppresses the STAT3-dependent angiogenic switch.

As discussed earlier (Section 6.5), RECK seems to have distinct functions in endothelial cells and mural cells. RECK in endothelial cells enhances ligand-specific signaling of WNT7A/B, whereas RECK in mural cells seems to stabilize blood vessels. Although the dynamics of RECK expression in vascular cells during tumor angiogenesis remain unknown, observations with cultured cells indicate that the level of RECK expression could be influenced by various factors, such as cell density, growth factors/cytokines, ambient oxygen concentration, and the cell type (see Table S10 and Table 5). In studying or discussing the roles of RECK in angiogenesis, we need to always make it clear in terms of which cell type (tumor, endothelial, mural, or other cell type) RECK is being studied.

### 9.3. Suppression of Cell Proliferation

The activities of RECK on suppression of cell proliferation and induction of apoptosis and cellular senescence have been reported (Table S4). Proposed mechanisms of these effects include suppression of the dimerization of a growth factor receptor (HER2) [114,118]; inhibition of ADAM17-mediated Notch cleavage/activation [115]; upregulation of cell cycle regulators [116]; downregulation of an E3 ubiquitin ligase (SKP2) [117]; enhancement of ER-stress response [63]; and suppression of DNA damage repair [119]. Yu et al. [223] reported that RECK promotes the growth of pancreatic tumor cells by enhancing WNT7 signaling, although the finding disagrees with previous reports [113,224]; the reason for this discrepancy remains unknown.

### 9.4. Response to Therapy

Shen et al. [225] performed whole exome sequencing of HCC tissues and matched blood samples from patients who underwent transarterial chemoembolization (TACE) and found that the rate of *RECK* somatic mutations was significantly higher in tumors exhibiting a poor response to TACE. Although the clinical utility of this treatment may now be debatable, an exceptionally high frequency of *RECK* mutations was detected in HCC in this study (11%), which warrants further studies in other cohorts, and it should also be interesting to know the exact natures (e.g., positions, biological effects) of these mutations and how these mutations (presumably in tumor cells) affect TACE outcome.

### 9.5. Novel Findings in Model Animals

For 20 years, RECK has been classified as a tumor suppressor based on two lines of observations: (1) RECK is downregulated in various types of cancer compared to their normal or less malignant counterparts (see Section 7) and (2) RECK suppresses various attributes of cancer cells as demonstrated using cultured cells or in transplantation experiments (see Section 6.3). Recently, additional evidence indicating that reduced RECK expression results in increased tumorigenesis in vivo began to emerge.

In 2018, Kumari et al. [45] reported that in zebrafish, heterozygous *reck* mutation not only accelerated the incidence of nerve tissue tumors induced by heterozygous rpL35 (a ribosomal protein gene) or tp53 mutation but also shifted the spectrum of tumor types to an increase in peripheral primitive neuroectodermal tumors. Remarkably, these tumors had loss of heterozygocity (LOH) in the *reck* locus, indicating that *reck* is inactivated following Knudson’s two-hit theory [226], just like classical tumor suppressor genes, in this system.

In 2023, Masuda et al. [113] reported that in a mouse model of pancreatic ductal adenocarcinoma (PDAC) called KPC, pancreatic *Reck* deletion dramatically augmented the spontaneous development of PDAC with a mesenchymal phenotype, which was accompanied by increased liver metastases and decreased survival, providing the first experimental evidence that RECK is a bona fide tumor suppressor in mammals. Their experimental data further suggest that RECK suppresses EMT of PDAC cells in this system.

In 2024, Matsuzaki et al. [227] reported that in RECK-Hypo mice (expressing RECK at <20% of the normal level; see Section 6.4), the incidence of spontaneous pulmonary adenomas was substantially increased. They also compared the growth rates of a syngeneic, tumorigenic cell line transplanted into RECK-Hypo and normal control mice and found a higher growth rate in the RECK-Hypo mutant mice, demonstrating non-cell-autonomous tumor suppression by RECK. A typical approach in previous studies was to manipulate (overexpress, knockdown, or knockout) the *RECK* gene in human tumor cell lines that were then transplanted into immunodeficient mice. The experiment by Matsuzaki et al. was unique in two ways: they manipulated the *Reck* gene in host mice rather than tumor cells and used immunocompetent mice as recipients. Although the exact mechanism of non-cell-autonomous tumor suppression by RECK is yet to be elucidated, a contribution by the host immune system is conceivable. Indeed, they found an increased proportion of regulatory T cells in the spleen and elevated levels of TGFβ1 in the peripheral blood of RECK-Hypo mice compared to the normal mice, suggesting an immune-suppressive tumor microenvironment in these mice. The latter finding is consistent with their previous finding [104] that RECK is required for the formation of proper fibrillin fibers, a major ECM component known to serve as a tissue reservoir for precursors of TGFβ-family cytokines.

These and forthcoming model systems directly demonstrating the tumor suppressor activity of RECK will accelerate our understanding of its mechanisms of actions as well as the development of novel clinical applications based on RECK.

## 10. Involvement in Non-Neoplastic Disorders

Altered RECK expression has been found in a variety of disorders and physical conditions other than cancer (Table S13), as well as in animal disease models (Table S14). The alteration in RECK expression can be a cause, result, or independent phenomenon of the diseases/conditions. Causal involvement of RECK has been suggested so far in the six diseases summarized in the following sections: the number may increase as studies advance.

Whether the altered expression of RECK is a cause or a result of a disease is important when one attempts to manipulate RECK expression (see Section 8) to improve or prevent the condition. When decreased RECK is one of the causes of a disease, reagents that upregulate RECK may be useful. It should be noted, however, that RECK-inducing reagents may also increase the risk of diseases that are promoted by RECK upregulation. In a similar manner, when increased RECK is one of the causes of a disease, reagents that downregulate RECK may be useful, although such reagents may increase the risk of other diseases that are suppressed by RECK.

In addition, when decreased RECK is a result of a disease, decreased RECK may be a part of the defense and/or repair response required for recovery from the disease. In such cases, RECK-inducing reagents may worsen the condition. For instance, Tang et al. [228] reported that RECK was downregulated in the rat brain after transient cerebral ischemia and that the treadmill exercise (rehabilitation known to accelerate functional recovery) enhanced RECK downregulation, although the causal involvement of RECK downregulation in functional recovery from cerebral ischemia has yet to be established.

Thus, it is important to understand the exact roles of RECK in diseases that upregulate or downregulate RECK.

### 10.1. Cardiac Fibrosis

Systematic studies by Chandrasekar’s group have revealed that some substances that promote (Angiotensin-II, IL-18) or suppress (acetylsalicylic acid, docosahexaenoic acid, HDAC inhibitors) cardiac fibrosis in mice may modulate the expression of RECK in cardiac fibroblasts and that RECK downregulation may be causally involved in this disorder [108,188,229,230]. They also explored the mechanisms by which RECK expression could be modulated by these and other substances as well as the roles of RECK in vascular [109,189,194,231,232,233], renal [216], and hepatic [24] diseases.

### 10.2. Restenosis After Vascular Angioplasty

MMP2 and MMP9 have been implicated in restenosis after vascular angioplasty. In the rabbit carotid artery balloon injury model, Liu et al. [234] observed transient upregulation followed by significant downregulation of *RECK* mRNA and sustained upregulation of *MMP2* and *MMP9* mRNAs in the injured tissues. They also found that in human vascular smooth muscle cells, RECK knockdown resulted in upregulation of *MMP2* and *MMP9* mRNAs, suggesting the suppressive effect of RECK on restenosis.

### 10.3. Osteoporosis

Zhao et al. [235] reported that in mesenchymal stem cells from the osteoporosis model mice generated by bilateral oophorectomy, downregulation of miR-21 and upregulation of RECK were observed. RECK knockdown in vivo with a lentiviral vector expressing RECK shRNA (transpatellar intramedullary injection into the femoral bone marrow) improved the condition, suggesting a causal involvement of RECK in this disorder.

### 10.4. Alzheimer’s Disease

ADAM10, the alpha secretase for amyloid precursor protein (APP), cleaves APP to generate neuroprotective soluble APPα (sAPPα), which precludes the generation of amyloid beta (Aβ), a defining feature of Alzheimer disease (AD) pathophysiology. The findings by Nakamura et al. [236] suggest that glycerophosphodiester phosphodiesterase 2 (GDE2) promotes ADAM10-mediated APP cleavage by shedding RECK from the neuronal cell surface. In the AD brain, GDE2 is often abnormally sequestered inside neurons and membrane-tethered RECK tends to be elevated. Genetic ablation of GDE2 in mice resulted in increased membrane RECK and an AD-like phenotype including reduced sAPPα, increased Aβ, and synaptic protein loss. Genetic reduction of RECK in this system improved the conditions, restored the balance of APP processing, and rescued synaptic protein loss. These findings support the idea that RECK promotes AD.

### 10.5. Metabolic Dysfunction-Associated Steato Hepatitis (MASH)

The transcription factor FXR is known to play an essential role in maintaining lipid and carbohydrate homeostasis. FXR-null mice display abnormal bile salts, triglyceride levels, and impaired insulin sensitivity. Activation of FXR by synthetic agonists results in significant protection from cholestasis, atherosclerosis, liver fibrosis, and inflammation. Peng et al. [198] demonstrated that *RECK* was a target of FXR in mammals (see Section 8.3.5), that *Reck* mRNA was downregulated in the liver of FXR-deficient mice, and that FXR agonists (WAY-362450, GW4064) could upregulate the expression of *Reck* mRNA and protein in the liver of wild-type mice as well as in mouse hepatocytes in culture. When wild-type mice were fed a methionine/choline-deficient diet for 4 weeks to induce MASH, the levels of FXR and RECK in the liver were reduced but could be restored by WAY-362450. These findings support the idea that RECK, acting downstream of FXR, suppresses MASH.

Palladini et al. [237] reported contrasting data, indicating that RECK protein was upregulated in the liver of rats fed a methionine/choline-deficient diet for 2 weeks, although the effect became smaller at later time points (4 and 8 weeks). Whether the discrepant findings in these two studies reflect the difference in animal species or other factors remains unclear.

Di Pasqua et al. [238] reported that a high-fat diet downregulated liver RECK mRNA and protein in LEP (ob/ob) mice. An FXR agonist, INT-787, restored RECK expression and alleviated MASH-associated symptoms, supporting the idea that RECK suppresses this disease.

Dashek et al. [239] examined clinical samples from human patients and found that the level of RECK expression was significantly lower in the livers of patients with MASH and was inversely correlated with the severity of the disease. They also found that RECK expression was downregulated when MASH was induced by a Western diet in wild-type mice and that hepatocyte-specific RECK overexpression significantly reduced hepatic pathology in this model. They went on to generate a new *Reck* mutant allele, in which exons 2 and 3 are flanked by two loxP sites, and found that hepatocyte-specific *Reck* inactivation resulted in increased liver inflammation and fibrosis even in the animals fed the control (non-Western) diet [240].

Taken together, these findings indicate that RECK suppresses MASH (at least in mice and humans) and warrants further investigation of RECK inducers (such as FXR agonists; also see Table 6) as a promising approach in preventing this disease.

### 10.6. Chronic Obstructive Pulmonary Disease (COPD)

Li et al. [241] reported that the levels of RECK mRNA and protein were lower than the control in lung tissues (airway epithelial cells) from a rat model of COPD induced by cigarette smoke.

By examining clinical tissue samples, Wang et al. [242] found that RECK expressed by airway epithelial cells in the lung was significantly diminished in COPD patients. The levels of RECK in sputum and plasma were also significantly decreased in COPD patients. The level of RECK was inversely correlated with the levels of IL-6 and IL-8 in the plasma. Cigarette smoke extract (CSE) downregulated RECK in bronchial epithelial cells, and recombinant RECK protein suppressed CSE-stimulated migration of neutrophiles and secretion of IL-6 and IL-8, indicating a suppressive role of RECK in COPD pathogenesis. An earlier finding indicated that IL-18 suppressed RECK expression in cardiac fibroblasts [188]. It would be interesting to determine whether IL-18 suppresses RECK expression in airway epithelial cells and whether RECK suppresses IL-18 production in cardiac fibroblasts, for such observations may reveal a negative feedback loop between RECK and IL-18.

## 11. Perspective

**Figure 14:**
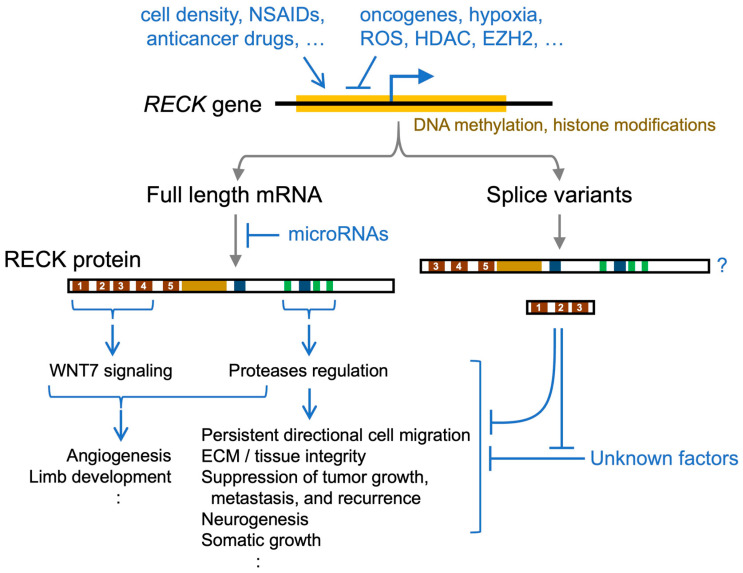
Summary of our current knowledge on what RECK does and how RECK expression is regulated.

RECK is a unique GPI-anchored multidomain protein that regulates the proteolytic cleavage of pericellular proteins, affecting the interactions among them. The expression of RECK is sensitive to various extracellular stimuli and affects cellular proliferation, migration, and gene expression (Figure 14). Devastating effects of *Reck* deficiency in mouse embryos indicate indispensable roles of RECK during mammalian development. Although previous studies have yielded substantial knowledge concerning the nature and functions of RECK, it is likely that we have solved only a small part of the whole puzzle to date. We therefore end this review with a list of outstanding open questions, hoping to suggest possible starting points for further explorations.

### 11.1. Molecular Functions

The ability of RECK to regulate proteolysis likely underlies a large part of its biological activities such as suppression of cell migration and proliferation. In this respect, how the spatiotemporal regulation of RECK affects the dynamic process of ECM remodeling to determine cell fate and behavior is a basic question of interest yet to be fully addressed. Multiple lines of evidence indicate that protease substrates that are protected by RECK extend beyond ECM components and include other cell surface proteins such as transmembrane receptors. It is important to clarify the repertoire of such substrates and to what extent their stability depends on RECK.

Recent studies revealed the unexpected activity of RECK in binding WNT7 and its ability to greatly enhance its downstream signaling (see Section 6.5). This activity is probably independent from RECK regulation of proteolysis. This raises the question as to how many independent molecular functions RECK has and which domains are responsible for the respective functions. Our knowledge of the functions and mechanisms of actions of the shorter *RECK* splice variants is limited. Mechanisms regulating *RECK* alternative splicing are also unknown. The effect of RECK on modulating gene expression has been reported by many studies, and yet the mechanisms (or pathways) by which this cell surface molecule affects gene expression remain unknown. Although some cytoplasmic proteins have been reported as RECK-binding partners (Table S2), the physiological relevance of such interactions needs to be tested more rigorously (see Section 3.2).

### 11.2. Biological Functions

At the cellular level, ample evidence indicates that RECK suppresses cell migration and matrix invasion. Some studies suggest that RECK enables cells to perform directionally persistent migration. While the involvement of fibronectin, integrins, and focal adhesions has been suggested, exactly how RECK makes directionally persistent migration possible remains unclear. Likewise, the mechanisms of how RECK affects cell proliferation, cell cycle, and EMT remain largely unknown.

At the tissue and organ level, a relatively large number of studies focusing on the roles of RECK in the development of vascular and neural systems have been reported. In contrast, our knowledge regarding its roles in musculoskeletal systems is limited. As for the functions of RECK in the brain, although our attention has been focused on brain angiogenesis in recent years, RECK is also known to affect neuronal differentiation and adult neurogenesis, and studies in this area should also be expanded.

### 11.3. Regulation of RECK Expression

Except for SP1 family members and FXR, little is known about the transcription factors directly regulating *RECK* expression. Likewise, besides DNA methylation and EZH2, little is known about epigenetic control of *RECK* gene expression. How its alternative splicing is controlled also remains largely unknown. *RECK* mRNA is a potential target of many microRNAs as well as some other non-coding RNAs (Table 4 and Table S9). However, non-coding RNAs often have multiple targets, and it is not always clear how significant the contribution of RECK-targeting by a given non-coding RNA is in the biological activities of that non-coding RNA.

Reagents that affect *RECK* expression may provide tools to explore the mechanisms of *RECK* gene regulation. For instance, in an attempt to find targets of an *RECK* inducer (DSK638), KLF2 (a transcription factor recognizing SP1 sites) was found to be a key transcription factor associated with switching *RECK* gene expression ON (in the presence of the drug) and OFF (in the absence of the drug; see Section 7.1.2 and Section 8.4.2). Thus, knowledge of transcription factors and signaling pathways that regulate *RECK* expression should facilitate the discovery of new *RECK*-regulating drugs.

A recent paper by Higashi et al. [233] indicates that EF24, a curcumin analog with improved bioavailability and bioactivity, suppresses IL-18-induced proliferation and migration of human aortic smooth muscle cells and that this involves MAPK-mediated upregulation of miR-342 which targets DNMT1, thereby inducing *RECK* expression due to promoter hypomethylation. In this case, several pieces of the puzzle (e.g., curcumin, MAPK signaling, DNMT1, and promoter methylation) fit into a coherent picture.

### 11.4. Clinical Relevance

The role of RECK as a tumor suppressor was initially suggested by gene manipulation studies using tumor xenograft models in immunodeficient animals. These studies have been supplemented by determination of RECK expression in human cancers, which have shown RECK downregulation in a variety of cancer specimens (Table 3). In addition, the recent finding by Shen et al. [225] that the rate of *RECK* somatic mutations was significantly high in HCC cases exhibiting a poor response to TACE agrees with the premise that RECK has tumor-suppressive properties. More studies using immunocompetent animals to explore the mechanisms of tumor suppression by RECK need to be carried out.

Altered RECK expression is also associated with many other diseases and with the animal models of these diseases (Tables S13 and S14). It is important to determine whether altered RECK expression is a cause, result, or irrelevant phenomenon of the disease, for instance, by using experimental model systems. Association of SNPs in the *RECK* locus with a disease of interest may be a valid approach to investigate the possible relevance of RECK to the disease, although the actual effects of each SNP on gene function need to be elucidated experimentally. Once the relevance of RECK to the disease of interest is established, studies on the mechanism of RECK’s action in that disease may lead to the discovery of new molecular functions of RECK and possible therapeutic/preventive agents for the treatment of the disease.

### 11.5. Applications

RECK-inducing drugs may be useful in treating or preventing diseases caused by RECK downregulation, and RECK-downregulating drugs may be useful in managing diseases caused by RECK overexpression. However, the effects of RECK inactivation as well as RECK overexpression in model animals should be carefully studied before moving in this direction, since such knowledge is needed in predicting potential side effects and recognizing the appropriate drug targeting in clinical applications. Notably, little is known about the effects of RECK overexpression in model animals.

Since RECK downregulation, methylation, and silencing have been found in many types of tumors, and since tumors with relatively high residual RECK expression tend to show better prognoses, RECK-inducing drugs are expected to be useful in treating or preventing cancers with low RECK expression. In the case of mammary tumors, RECK gene methylation is significantly correlated with recurrence of the disease, suggesting the potential utility of RECK-inducing drugs in adjuvant therapy. Several plant-derived components or crude plant extracts were found to show RECK-inducing activity (Table 6); long-term intake of such herbs may be a feasible approach in cancer chemoprevention once their safety is confirmed.

In the late 1990s and early 2000s, small molecular MMP inhibitors (MMPIs) were tested as anticancer drugs with disappointing results [243,244]. RECK is fundamentally different from these classical MMPIs not only in its molecular size but also its localized action near the cell surface (through GPI-anchoring), as well as its presence in normal tissues. These properties give us good reasons to expect that RECK-inducing drugs can be less toxic than classical MMPIs. Two pilot screenings of chemical libraries with cells harboring RECK-promoter-reporter constructs detected several compounds, including disulfiram and DSK638, capable of suppressing spontaneous skin-to-lung metastasis with no obvious acute toxicity in a mouse model (see Section 8.4.2 and Section 8.4.4). This line of study is worth extending in two ways: to evaluate the candidate compounds in more and/or better preclinical models and to extend the screening to find more and/or better therapeutic compounds.

Given our abundant knowledge of RECK-targeting microRNAs (Table 4), nucleic acid medicines targeting microRNAs might be another feasible approach. In this respect, the adenoviral vector expressing artificial lncRNA that targets multiple microRNAs [164] is interesting. Extracellular vesicles, such as exosomes, could be another strategy for delivering microRNAs into the cells. Interestingly, Liu et al. [245] found that the hydrogel containing exosomes secreted by mesenchymal stem cells from giant panda umbilical cords accelerated skin wound healing in mice and that the exosomes contained high levels of miR-21-5p. They speculate that miR-21-5p contributes to the accelerated wound closing by downregulating RECK (and PDCD4), thereby affecting cell migration, proliferation, and differentiation (see Section 6.2, Section 6.3 and Section 6.7).

In conclusion, although RECK is a unique molecule of interest in terms of both basic science and clinical applications, its regulation and mechanisms of actions are yet to be fully understood. For instance, we are not sure why mice with reduced RECK expression show reduced body size (see Section 6.9), why an *RECK* orthologue is found in *Drosophila* but not in *C. elegans*, why *RECK* (containing the unique CC domains) is conserved as a single gene in animals carrying *RECK* orthologues, and why this protein should carry two apparently unrelated functions, to regulate proteases and to enhance WNT7 signaling, in one molecule. These puzzles await solutions, and the answers may lead to important clinical applications.

## Figures and Tables

**Table 1:** Proteases regulated by RECK.

Enzyme	Substrates	Effects of RECK/Findings	References		
			First Author	Year	PMID
MMP2	gelatin (zymography)	Reduction of the active form	Oh	2001	11747814
		Downregulation	Herbster	2021	34066355
MMP7	fibronectin	Enzyme inhibition	Omura	2009	19022775
MMP9	oligopeptide	Enzyme inhibition	Takahashi	1998	9789069
	n.s.	Inhibition of transcription	Takagi	2009	19208844
MT1-MMP/ MMP-14	oligopeptide	Enzyme inhibition	Oh	2001	11747814
	n.s.	Enzyme binding and internalization (HT1080 fibrosarcoma)	Miki	2007	17329256
	n.s.	Protein upregulation (Xenopus A6 cells)	Wilson	2019	31799217
MMP17b	n.s.	Co-localized in dorsal root ganglia in zebrafish; co-immunoprecipitation with, and protease inhibition by, RECK in mammalian (COS-7) cells	Leigh	2013	24098510
CD13	n.s.	Enzyme binding and internalization (HT1080 fibrosarcoma)	Miki	2007	17329256
	n.s.	Co-localize with CD13 in caveolae clusters in cynoviocyte	Berg	2009	18648844
ADAM10	Notch ligands	Suppression of neuronal differentiation	Muraguchi	2007	17558399
ADAMTS10	fibrillins	Binding and protection of the enzyme	Matsuzaki	2018	30287421
				2021	32730638

n.s.: not studied or not specified.

**Table 3:** Altered RECK expression in neoplastic lesions in humans.

Tumor Type	First Report			Number of Subsequent Reports *
	First Author	Year	PMID	
**Lower expression in more malignant lesions**				
fibrosarcoma transformed fibroblasts	Takahashi	1998	9789069	
hepatocellular carcinoma	Furumoto	2001	11124835	4
pancreatic cancer	Masui	2003	12738734	1
breast cancer	Span	2003	12767082	11
non-small cell lung cancer	Takenaka	2004	15196549	10
colorectal cancer	Takeuchi	2004	15328199	6
prostate cancer	Ohl	2005	16211407	6
gastric cancer	Song	2006	16324834	5
ameloblastoma	Kumamoto	2006	16762015	1
cervical cancer	da Silva Cardeal	2006	17167534	3
esophageal cancer	Li	2007	18023103	4
oral cancer	Nagini	2009	19250857	1
neuroblastoma	Dong	2010	20579139	2
osteosarcoma	Xu	2010	20973064	1
ovarian cancer	Fejzo	2011	21432940	1
urothelial carcinoma/bladder cancer	Wittschieber	2011	21613799	3
nasopharyngeal carcinoma	Deng	2012	22844865	1
renal cell carcinoma	Rabien	2013	24131772	1
melanoma	Jacomasso	2014	24335752	1
oral squamous cell carcinoma	Yuan	2020	32269688	1
**Miscellaneous tumors or cells (PMID)**: hilar cholangiocarcinoma (16463672), glioma (16791855), peripheral nerve sheath tumors (16619545), pheochromocytoma, metastases (17957724), chondrosarcoma (20584302), skull base chordomas (19862564), cholangiocarcinoma (21076843), laryngeal cancer (21833719), middle ear squamous cell carcinoma (22178867), B-cell lymphoma (23141964), peripheral T-cell lymphoma (23833658), cutaneous squamous cell carcinoma (24356192), salivary adenoid cystic carcinoma (24765174), hepatoblastoma (25987077), myeloma (27936757), undifferentiated pleomorphic sarcoma (31509746), Wilms’ tumor (30841433), buccal mucosa squamous cell carcinoma (31618798), gallbladder cancer (32206004), endometrial carcinoma (32525042), head and neck cancer-associated fibroblasts (15313864)				
**Higher or heterogeneous expression in more malignant lesions**				
calcifying cyst odontogenic tumor	Prosdócimi	2014	24484176	
benign prostatic hyperplasia	Barbosa	2017	28506859	
gastric cancer	Yu	2024	38923741	

* Up to October 2025.

**Table 4:** MicroRNAs downregulating RECK.

MicroRNA	First Report(s)			Number of Reports *
	First Author	Year	PMID	
**a. Direct targeting validated**				
miR-7	Jung	2012	22761427	1
miR-15/16	Loayza-Puch	2010	20154725	4
miR-21	Hu	2008	18556655	43
	Gabriely	2008	18591254	
miR-25	Zhao	2014	24078004	2
miR-92	Lin	2013	23820254	3
miR-96	Zhang	2013	24366472	4
miR-135b	Li	2015	25537516	4
miR-181a-5p	Zhang	2023	36895489	1
miR-182	Hirata	2012	23226455	5
miR-195	Xu	2021	34790697	3
miR-200	Cheng	2016	27574450	5
miR-205 (mouse: miR-712)	Kim	2014	24812324	2
miR-221	Qin	2014	24269686	3
miR-222	Li	2012	22321642	2
miR-372/373	Loayza-Puch	2010	20154725	1
miR-374b-5p	Xie	2014	25516656	1
miR-497	Chen	2017	28098218	2
miR-544a	Zheng	2020	32525042	1
miR-590-5p	Shen	2016	27757042	3
**b. Direct targeting not validated**				
miR-125b	Shi	2012	22711523	1
miR-183	Silva	2015	26609496	2
miR-6516-3p	Tomita	2025	40045202	1

* Up to October 2025.

## Data Availability

No new data were created or analyzed in this study.

## Supplementary Materials

The following supporting information can be downloaded at https://www.mdpi.com/article/10.3390/life16010104/s1.

## Abbreviations

The following abbreviations are used in this manuscript:

ADAM	a disintegrin and metalloproteinase domain
ADAMTS	a disintegrin and metalloproteinase with thrombospondin motifs
COPD	chronic obstructive pulmonary disease
DRG	dorsal root ganglion
ECD	extracellular (or ecto) domain
ECM	extracellular matrix
EGFR	epidermal growth factor receptor
EMT	epithelial–mesenchymal transition
ERK	extracellular signal-regulated kinase
FA	focal adhesion
FXR	farnesoid X receptor
FZD	frizzled
GPI	glycosylphosphatidylinositol
GPR	G-protein coupled receptor
HCC	hepatocellular carcinoma
HDAC	histone deacetylase
HIF	hypoxia inducible factor
ICD	intracellular domain
LncRNA	long non-coding ribonucleic acid
LRP	low-density lipoprotein receptor-related protein
MASH	metabolic dysfunction-associated steatohepatitis
miR	micro ribonucleic acid
MMP	matrix metalloproteinase
NPC	neural precursor cell
NSAID	nonsteroidal anti-inflammatory drug
RECK	reversion-inducing cysteine-rich protein with Kazal motifs
SNP	single-nucleotide polymorphism
Sp1	specificity protein 1
TGFβ	transforming growth factor beta
TIMP	tissue inhibitor of metalloproteinase
WNT	wingless-type MMTV integration site
