# Supplementary material for: The Roles of the Membrane-Anchored Glycoprotein RECK in Animal Development, Tumor Suppression, and Beyond
Source: Life (Basel). 2026 Jan 11;16(1):104. doi: 10.3390/life16010104 (PMC12842694; doi:10.3390/life16010104)
Supplement: Supplementary file 1 [file life-16-00104-s001.zip › life-4048138-supplementary.pdf]

Supplementary Materials

Supplementary Tables

Table S1. Association of *RECK* SNPs with cancer

SNPs associated with cancer (The major alleles are shown in **bold**)			Cancer type (n, race/nationality)	Alleles in cancer**		Amino acid (position; domain)	Findings [OR (95%CI); p-value]	Reference		
ID	Flanking sequence	Position (GRCh38)		Protective	Promoting			First author	Year	PMID
**rs16932912, rs11788747, rs10972727, (and 10 others)**	(see reference)	(see reference)	healthy controls (157, caucasians)	n.a.	n.a.	(see reference)	Designed PCR primers for all major exons of *RECK*, and identified 13 SNPs in and around these exons.	Eisenberg	2002	12438739
**rs11452642**	ATTGAGGCTATA[–/T]GTGCCAAAGAGC*	upstream (chr9:36036414-36036415)	breat cancer (959, Swedish)	-	T	n.a.	The TT homozygocity, in conjunction with TIMP3 SNP (rs9619311; TT), was more frequent in tumors with negative staining for estrogen receptors [1.81 (1.03–3.21)] and progesterone receptors [2.10 (1.18–3.86)].	Lei	2007	17033924
**rs10814325**	GGACTAAGCCCT[**T**/C]GTTCTCAGGTGA	upstream (chr9:36036597)	breat cancer (959, Swedish)	C	T	n.a.	TC heterozygotes (n=150) showed better survival rate than TT homozygotes (n=782; p=0.02)			
			oral cancer [SCC] (341, Taiwanese male)	T	C		The TC or CC genotype was more frequent in patients than the control among people with betel quid chewing [7.62 (2.96–19.64); <0.0001]. Among patients with betel quid chewing, these genetypes were associated with lymph node metastases [2.26 (1.19-4.29); <0.05].	Chung	2011	21565829
			liver cancer [HCC] (135, Taiwanese)	T	C		The TC or CC genotype was more frequent in patients than in thealthy controls [1.85 (1.03–3.36)].	Chung	2012	22428065
			lung cancer [NSCLC] (304, Chinese)	T	C		The CC genotype was more frequent in patients than in healthy contorols [2.302 (1.514-3.501); 0.012].	Chen	2014	24510537
			liver cancer [HCC] (160, Chinese)	T	C		The CC genotype was more frequent in patients than in healthy controls [2.68 (1.35–5.34); 0.005].	Su	2014	25412941
			liver cancer [HCC+HCV] (30, Egyptian)	N/D	N/D		No significant difference in SNP frequency among HCV^+ HCC patients, HCV^+ cirrhosis patents, and healthy controls.	Fakhry	2016	27268601
**rs754745207**	GAAAATTATTGC[**G**/C]CCTCTATTAGT	exon 8 (chr9:36083487)	endometrial cancer (472+261, American)	G	C	Ala → Pro (168; CC3)	GWAS of Lynch syndrome patients (by exome sequencing) detected association of pooled RECK gene variations, including G to C at this site (p=0.0450) and rare variations at 7 other sites, with endometrial cancer.	Shivakumar	2019	31338326
**rs16932912**	GTTCACCCTGGA[**G**/A]TCACTGTACACC	exon 9 (chr9:36087879)	oral cancer [SCC] (341, Taiwanese male)	G	A	Val → Ile (275; between CC4 and CC5)	The GA or AA genotype was more frequent in patients than the control among people with betel quid chewing [25.33 (9.57–67.02); <0.0001] or smoking [18.57 (3.80–90.80); 0.0055].	Chung	2011	21565829
			ameloblastoma (30, Chinese)	G	A		The A allele was found in 11 samples and to associate with proliferation (Ki67-positive), invasion, and recurrence (p<0.05). The RECK protein level was lower in tumor tissues with the A allele than those without that allele (p<0.05).	Zhang	2017	28340422
**rs11788747**	TCCCTGTCTTCC[**A**/G]TACTTTTGTGTT	exon 13 (chr9:36105267)	oral cancer [SCC] (341, Taiwanese male)	A	G	Pro - Pro (520; E1)	The AG or GG genotype was more frequent in patients than the control among people with betel quid chewing [9.37 (3.92–22.39); <0.0001] or smoking [6.87 (1.57–30.05); 0.0049].	Chung	2011	21565829
			liver cancer [HCC] (135, Taiwanese)	A	G		The AG and GG genotypes are more frequent in patients than in healthy controls [1.85 (1.03–3.36)] and in tumors with distant metastases than those without distant metastases (p=0.003).	Chung	2012	22428065
			Wilms' tumor (97, Han Chinese)	A	G		The G allele was more frequent in Wilms' tumor patients than in healthy controls (p=0.042).	Yu	2015	26141647
			liver cancer [HCC] (104, Egyptian)	G	A		The GG genotype was less frequent in patients than in healthy controls [0.11 (0.03–0.36); <0.001].	Abd-Elfatah	2016	26921475
			liver cancer [HCC] (200, Egyptian)	A	G		The AG and GG genotypes were more frequent in patients than in healthy controls [3.807 (2.512–5.771); <0.001]. The G allele seems to have a dose-dependent positive effect on both lymph node and distant metastases (p<0.001).	Bahgat	2016	25278269
			colorectal cancer (130, Mexican)	G	A		The GG genotype was less frequent in patients than in healthy controls [0.33 (0.16-0.70); 0.006].	Márquez-González	2022	35444107
**rs10972727**	AGAAGATGACCG[**T**/A]CGTACCTTCACA	exon 15 (9:36110066)	oral cancer [SCC] (341, Taiwanese male)	T	A	Arg - Arg (625; 10 aa N-terminal side of K1)	The AT or AA genotype was more frequent in patients than the control among people with betel quid chewing [10.65 (4.35–26.06); <0.0001] or smoking [11.52 (2.31–57.56); 0.0026].	Chung	2011	21565829
			colorectal cancer (130, Mexican)	A	T		The AA genotype was less frequency in patients than in healthy controls [0.38 (0.19-0.77); 0.011].	Márquez-González	2022	35444107

Abbreviations: SNP, single nucleotide polymorphism; GRCh38, reference human genome sequence; SCC, squamous cell carcinoma; HCC, hepatocellular carcinoma; HCV, hepatitis C virus; NSCLC, non-small cell lung cancer; OR, odds ratio; CI, confidence interval; PCR, polymerase chain reaction; n.a., not applicable; N/E, no difference; *Designated "-585 -/T" in Lei et al.; **Roles of the indicated alleles in cancer formation suggested by the referenced study.

Table S2. RECK-interacting proteins

Molecule	Annotation	Methods	Reference		
			First author	Year	PMID
Tgat	Oncoprotein; a splice variant of TRIO (RhoGEF)*	yeast two-hybrid screening using the unique C-terminal 15 amino acid residues of Tgat as a bait / co-immunoprecipitation	Mori	2007	17328864
GRP78 / HSPA5	Glucose-regulated protein; a member of HSP70 family member; ER chaperon	identification of RECK-bingind proteins with LC-MS/MS	Chen	2013	24084596
ITGB1, GAL1, IL6R, IL6ST, uPAR	β1-integrin, galectin-1, interleukin-6 receptor, gp130, urokinase-type plasminogen activator receptor	protein array binding assay with breast cancer (Hs606T) cell proteins co-precipitated with RECK	Walsh	2015	24084596
GPR124	G-protein coupled receptor 124; also known as adhesion G protein-coupled receptor A2 (ADGRA2)	overexpression and cell aurface binding assay	Cho	2017	28803732
Wnt7a/b	wingless-type MMTV integration site family, member 7a	overexpression followed by cell surface binding assay / proximity ligation assay	Eubelen	2018	30026314
ADAMTS10	a disintegrin-like and metalloprotease with thrombospondin type 1 motif 10	yeast two-hybrid screening with near full-length RECK as a bait / proximity ligation assay	Matsuzaki	2018	30287421
GPR124, WNT7(monomer)	(see above)	chemical cross-linking of rat brain blood vessels followed by affinity chromatography with anti-GPR124 resin and MS identification / co-immunoprecipitation	Vallon	2018	30304675
Wnt7a	(see above)	overexpression followed by cell surface binding assay	Cho	2019	31225798
CRMP2	collapsin response mediator protein 2; also known as dihydropyrimidinase like 2 (DPYSL2)*	co-immunoprecipitation	Lin	2020	32778769
KEAP1	Kelch like ECH associated protein 1*	co-immunoprecipitation	Wei	2024	39476567
ADAM10/15, etc.	a disintegrin and metalloproteinase domain 10 and 15; see Table S3 of Heiden et al. for other proteins	induced expression of biotin-tagged RECK in mouse brain endothelial cell line, chemical cross-linking, avidin pulldown, SDS-PAGE, and LC-MS/MS	Heiden	2025	40914247

*cytoplasmic protein

Table S3. Effects of RECK on the behavior of normal cells

Cell type	Source(s)	Effects of RECK / findings	Reference		
			First author	Year	PMID
fibroblast	*Reck* $^{-/-}$ mouse embryo	Stabilizes focal adhesions, anterior-posterior polarity, and directional persistence in migration.	Morioka	2009	19169281
fibroblast	Mouse heart	Inhibition of Angiotensin-II-induced cell migration	Siddesha	2013	24095877
fibroblast	NIH3T3, primary cells from mouse skeletal muscle and skin	Downregulates β1-integrin and inhibits skin wound healing.	Gutierrez	2015	26247610
fibroblast	human forskin	RECK variant 1 (full length) slows cell migration, while RECK variant 5 (see Fig. 1) promotes cell migration and tends to be more abundant in proliferating cells, TGF-β-treated cells, and tumor cells.	Lee	2018	29874120
fibroblast	human forskin	RECK variant 1 decreases acetylated tubulin and decreases detyrosinated (Glu) tubulin; RECK variant 5 has opposing effects.	Lee	2019	30704758
mesenchymal stem cell	adipose tissue of human breast	Data implicate that RECK inhibits tumor-oriented cell migration.	Senst	2013	23143214
smooth muscle cell	human aorta	Suppresses IL-17-induced, TRAF3IP2-dependent MMP13-expression, cell proliferation, and migration.	Mummidi	2019	31074012

Table S4. Effects of RECK on cell proliferation

Cell type	System / cell line	Method*	Effects of RECK	Reference		
				First author	Year	PMID
breast cancer	SKBR3	OE	Suppression of cell proliferation	Hong	2011	21255571
gastric cancer	MKN45-133[+]	OE	Suppression of anchorage-independent proliferation (soft agar) and tumorigenic growth (nude mouse)	Hong	2014	23881612
malignant peripheral nerve sheath tumor	STS26T, 90-8TL, S462, T265T, MC2633	OE	Suppression of cell proliferation	Kumari	2018	29805750
cervical cancer	SiHa, SW756	OE	Suppression of cell proliferation *in vitro* (soft agar and spheroid) and tumorigenic growth in nude mouse	Herbster	2021	34066355
fibroblast (mouse)	MEF	KO	Germline *Reck* knockout results in accelerated cell proliferation and early escape from cellular senescence	Kitajima	2011	20890302
osteosarcoma	SaOS-2	OE	Suppression of cell proliferation, Matrigel invasion, and colony formation (collagen gel) *in vitro* and tumorigenic growth in an orthotopic nude mouse model	Clark	2011	21287525
colon cancer	SW620	OE	Cellular senescence	Yoshida	2012	22158033
kidney epithelial cells	HEK293	(1) KD<br>(2) OE	(1) Increased cell proliferation and tumorigenicity<br>(2) Suppression of hypoxia-induced cell proliferation	Lee	2013	24376819
neuroblastoma	SH-SY5Y, BE(2)-M17	OE	Eenhanced cisplatin-induced cell death	Chen	2013	24084596
breast cancer	SKBR3	OE	Increase sensitivity to anticancer drugs such as cisplatin, camptothecin, and etoposide	Hong	2015	26396917
HRAS^{G12V}-transformed kidney epithelial cell line (mouse)	MKER	TP	Slower tumor growth in wild type mice than in mice with reduced RECK expression (Reck<tm1Noda/ tm2.2Noda>), demonstrating non-cell-autonomous tumor suppression by RECK.	Matsuzaki	2024	39104026

* OE: overexpression; KD: knockdown; KO: knockout; cKO: conditional knockout; TP: transplantation

Table S5. Roles of RECK in animal development

Experimental system / materials	Method*	Phenotype / finding	Reference		
			First author	Year	PMID
mouse	KO	Embryonic death at ~E10.5 with smaller body size, abdominal hemorrhage, reduced tissue integrity, primitive vascular plexus, fragile neural tube, and increased tissue gelatinase activity.	Oh	2001	11747814
**1. Vascular system**					
human microvascular endothelial cell (hMVEC)	Ab	TIMP2-overexpression upregulates RECK and suppresses cell migration.	Oh	2004	15604273
human umbilical vein endothelial cell (HUVEC)	KD	ANG1 induces RECK expression. RECK knockdown results in defective vascular tube formation and cellular senescence.	Miki	2010	20407016
mouse	cKO	*Reck* KO from E11 results in smaller embryos with severe hemorrhage at E15.5.	Chandana	2010	20691046
tissue-specific KO mouse	cKO	*Reck* KO in vascular mural cells results in death at ~E10.5. *Reck* KO in vascular endothelial cells lead to late embryonic lethality with severe intra-cranial hemorrhage.	Almeida	2015	26658478
**2. Limb**					
hypomorphic mutant mouse<br>Reck<tm1Noda/ tm2.2Noda><br>(RECK protein: ~20% of normal level)	Hypo	Limb abnormalities, including right-dominant, forelimb-specific defects in postaxial skeletal elements and frequent outgrowth of nail-like protrusions on the dorsal tips of all limbs. The phenotypes overlap with those of *Wnt7a* KO mice.	Yamamoto	2012	23213437
**3. WNT7 signaling**					
1) zebra fish<br>2) a cell line derived from human embryo kidney (HEK293)	1) KD<br>2) OE	1) *reck* morpholino induces brain vascular defects reminiscent of that induced by *gpr124* mutations.<br>2) GPR124 and RECK enhance Wnt7a/b-triggered canonical WNT signaling detected by TOP-flash reporter assay.	Vanhollebeke	2015	26051822
zebra fish	RM	A fish mutant, *nft* ($Reck^{C254Y}$), lacks most of the intracerebral central arteries, but not other brain blood vessels.	Ulrich	2016	26657775
tissue-specific hypomorphic mutant mouse<br>Reck<tm1.1Noda>/Reck<tm1.1Noda>;Tie2-Cre<br>Reck<tm1.1Noda>/Reck<tm1Ito>/Noda; Tie2-Cre	cKO	Hemorrhage and BBB breakdown in the forebrain and spinal cord at E13.5, a phenotype reminiscent of that in Gpr124-deficient mice, accompanied by attenuation of canonical WNT signaling. Genetic interactions with Gpr124, Wnt7a/b, and Norrin found. *Reck* mutations that impair GPR124-binding partially disrupts *Reck* function in CNS angiogenesis.	Cho	2017	28803732
a cell line derived from human embryo kidney (HEK293)	KO/OE	Loss of WNT7A/B binding to the cell. RECK CC4 domain and four conserved residues (V241, F251, L252, K262) in the linker domain of WNT7A are critical for this binding. GPR124 may act as a switch which turns RECK from an inhibitor to an activator of WNT7A-signaling.	Eubelen	2018	30026314
1) blood vessels from rat brain<br>2) a cell line derived from human embryo kidney (HEK293)	1) PC<br>2) KO/OE	1) RECK was idenfitied as a major binding partner for GPR124.<br>2) A soluble ectodomain of GPR124 promotes RECK/WNT7-dependent canonical WNT signaling. WNT7A binds RECK but not GPR124. RECK may stabilize newly produced WNT7A and relays it to the classical WNT receptor complex (FZD-LRP5/6). GPR124 may promote cell-surface localization of RECK.	Vallon	2018	30304675
1) a cell line derived from human embryo kidney (HEK293)<br>2) *Reck* mutant mouse (2 substitutions):<br>Reck<P256A,W261A/P256A,W261A><br>3) *Reck* mutant mouse (2 substatutions / hypomorph):<br>Reck<P256A,W261A / tm1.2Noda><br>4) hypomorphic *Reck* mutamt mouse<br>Reck<tm1.2Noda / tm1.2Noda>	1) KO/OE<br>2) SSM<br>3) SSM + Hypo<br>4) Hypo	1) Two amino acid residues (P256 and W261) in the CC4 domain of RECK is critical for WNT7-FXD-GPR124-RECK complex formation and signaling.<br>2) Homozygous $Reck^{P256A,W261A}$ mutations result in mid-gestation lethality with cranial hemorrhage like *Reck* KO.<br>3) Heterozygous $Reck^{P256A,W261A/\Delta ex2}$ mutations result in embryonic death starting from E13.5 with cranial hemorrhage and hypoplasia of anterior limbs.<br>4) $Reck^{\Delta ex2/\Delta ex2}$ mice survive up to P0 with severe defects in CNS angiogenesis.	Cho	2019	31225798
1) Neural precursor cell-specific KO mouse:<br>Reck<tm2.1Noda / tm2.1Noda>;Foxg1-Cre<br>2) a cell line derived from human embryo kidney (HEK293)	1) cKO<br>2) KO/OE	1) Reck KO in neural precursor cells (NPCs) results in neonatal death with forebrain hemorrhage like endothelial *Reck* KO.<br>2) NPC-expressed RECK may non-cell-autonomously promote forebrain angiogenesis through contact-dependent enhancement of WNT signaling in endothelial cells.	Li	2019	31445376
hypomorphic mutant mouse	Hypo	Genetic interactions in terms of limb abnormality can be detected between Reck and Wnt7a as well as Reck and Gpr124, indicating that WNT7/GPR124/RECK signaling system functions as an integral unit in limb development.	Wang	2022	35552394
zebra fish	KO	*Reck* inactivation leads to defects in the glut1+ (BBB) vasculature but not in the plvap+ (fenestrated) choroid plexus vasculature in the brain.	Parab	2023	37191285
**4. Neural system**					
mouse	KO	Precocious neuronal differentiation probably due to attenuated Notch signaling caused by deregulated ADAM10 and consequent excessive shedding of Notch-ligands.	Muraguchi	2007	17558399
1) heterozygous global KO mouse<br>2) lineage tracing with mouse carrying a Cre-reporter locus and the Reck<tm3.1(cre/ERT2)Noda> allele	1) Hypo<br>2) LT	1) Increased brain infarction after transient cerebral ischemia. The number of RECK-positive neural precursor-like cells increases progressively in the hippocampus after the ischemia.<br>2) *Reck* expression is induced at different timing in different areas of the hippocampus after ischemia.	1) Wang<br>2) Matsuzaki	2010<br>2018	20796170<br>29508517

zebra fish	1) RM<br>2) KD	1) A mutant, termed *sdp,* with abnormal dorsal root ganglion (DRG) was found to have *reck* mutation. The phenotype suggests that RECK is essential for proper migration of sensory neuron precursors.<br>2) A novel Mmp gene, Mmp17b, showing a segmental expression pattern along the embryonic trunk reminiscent of the intersomitic vasculature was identified. MMP17B is GPI-anchored, exhibits similar expression patterns with RECK, and its knockdown results in a phenotype similar to that of *sdp*, suggesting their cooperative functions in NC cell migration.	1) Prendergast<br>2) Leigh	2012<br>2013	22296847<br>24098510
1) chicken embryo<br>2) a cell line derived from human embryo kidney (HEK293)	1) KD, OE<br>2) OE, PC	1) GDE2, known to promote differentiation of spinal motor neurons, share similar expression pattern with RECK in the ventricular zone of spinal cord. RECK knockdown results in reduced Notch-signaling and precocious neuronal differentiation. GDE2-overexpression shows similar effects which is suppressed by RECK-co-overexpression.<br>2) GDE2-overexpression promotes release of cell surface RECK by cleaving its GPI-anchor.	Park	2013<br>2021	23329048<br>33731436
rat pheochomocytoma cell line (PC12)<br>mouse teratocarcinoma cell line (P19)	OE	*Reck* expression is upregulated in differentiated cells. *Reck* overexpression in P19 cells, however, reduces the efficiency of neuronal differentiation.	Trombetta-Lima	2021	33619662
**5. Muscle**					
1) mouse embryos<br>2) mouse myogenic cell line (C2C12)<br>3) embryo cells in culture	1) HC<br>2) OE<br>3) KO	1) Co-expressed with MRF4 in differentiating myoblasts at E13.5 and E14.5.<br>2) MyoD represses and MRF4 activates *Reck* transcription. Overexpression of RECK suppresses myotube formation.<br>3) The cells from *Reck* KO mice form myotubes more efficiently than the cells from normal mice.	Echizenya	2005	16007210
mouse diaphragm	HC	Expressed at the neuromuscular junctions (secondary folds and subsynaptic intracellular compartments in muscle cells) in the diaphragm from E18.5.	Kawashima	2008	17953659
Drosophila	KD	A direct transcriptional target of Notch signaling in muscle progenitor cells. KD during embryogenesis results in flight-deficiency ("held out wing"). Required for proper wing muscle development in the late stage (cell migration?)	Pézeron	2014	25217625
1) mouse myogenic cell line (C2C12)<br>2) $BaCl_2$-induced muscle damage in mice	1) KD<br>2) Hypo	1) Transient upregulation during myogenic differentiation. RECK-knockdown results in reduced Notch signaling, enhanced Myogenin and Myosin expression, and thicker myotubes.<br>2) Transient upregulation after muscle damage. Accelerated myofiber regeneration in *Reck*$^{+/-}$ mice with reduced fibrotic ECM accumulation.	Gurierrez	2021	33811686
**6. Skeletal system**					
1) mouse embryos<br>2) mouse chondrogenic cell line (ATDC5)	1) ISH<br>2) OE, KD	1, 2) Abundantly expressed in chondrocytes.<br>3) Progressively upregulated during chondrogenic differentiation. Both OE and KD suppress cartilaginous nodule formation, suggesting positive and negative effects of RECK during chondrogenesis, probably at different stages.	Kondo	2007	17298979
mouse osteogenic cell line (MC3T3-E1)	IB	Downregulated after osteogenic differentiation.	Zambuzzi	2009	18989628
bone marrow-derived human mesenchymal stem cell (hMSC)	KD	RECK is downregulated after adipogenic differentiation and upregulated after osteogenic differentiation. *Reck* KD results in upregulation of *TIMP2* mRNA, increased migration speed, increased adipogenic differentiation, decreased steogenic differentiation, decreased level of β-catenin, and attenuated β-catenin signaling.	Mahl	2016	26459448
mouse osteogenic cell line (MC3T3, subclone 4)		Upregulated after osteogenic differentiation induced by fibroblast (NIH3T3-E1)-derived conditioned medium.	Costa Fernandes	2020	31676316
**7. Blood cells**					
bone marrow and peripheral blood (mouse and human)	Ab	Circulating human $CD34^+$ cells have higher MT1-MMP and lower RECK compared with bone marrow (BM) cells. G-CSF increases MT1-MMP and decreases RECK in mouse BM cells in a PI3K/ Akt-dependent manner, mobilizing $CD34^+$ cells. This effect of G-CSF is inhibited by anti–MT1-MMP and promoted by anti-RECK antibodies.	Vagima	2009	19197139
hypomorphic mutant mouse<br>Reck<tm1Noda/ tm2.2Noda>	Hypo	Increased levels of TGFβ1 in the plasma and Treg cells in the spleen	Matsuzaki	2024	39104026
**8. Somatic growth**					
1) hypomorphic mutant mouse<br>Reck<tm1Noda> / Reck<tm2.2Noda><br>2) inducible KO mouse<br>Reck<tm2.1Noda> / Reck<tm3.1(cre/ERT2)Noda>	1) Hypo<br>2) cKO	Smaller body size and decrease in the levels of three cell surface receptors involved in the growth hormone/insulin-like growth factor 1 axis: i.e., GHRHR and GHSR in the pituitary gland and GHR in the liver.	Ogawa	2020	32165171

* KO: knockout; Ab: antibody; KD: knockdown; cKO: conditional knockout; Hypo: hypomorphic mutation; OE: overexpression; RM: random mutagenesis; PC: protein chemistry; SSM: site-specific mutagenesis; LT: lineage tracing; HC: histochemistry; ISH: in situ hybridization; IB: immunoblot assay

Table S6. Mutant alleles of mouse *Reck*

Allele name	Alternative name	Description	Reference			RIKEN ID
			First author	Year	PMID	
Reck<tm1Ito>	-	Exon 1 replaced with PGKneo	Oh	2001	11747814	RBRC05892
Reck<tm1.1Noda>	R2, flex2	Exon 2 floxed	Chandana	2010	20691046	RBRC09352, CDB0488K
Reck<tm1.2Noda>	△ex2	Exon 2 deleted<br>severe hypomorph	Cho	2017	28803732	
Reck<tm1Noda>	Low, R2neo	Exon 2 floxed, PGKneo undeleted<br>mild hypomorph	Yamamoto	2012	23213437	RBRC10232
Reck<tm2.1Noda>	R1, flex1	Exon 1 floxed	Yamamoto	2012	23213437	RBRC06577
Reck<tm2.2Noda>	△	Exon 1 deleted	Yamamoto<br>Almeida	2012<br>2015	23213437<br>26658478	RBRC10233
Reck<tm3.1(cre/ERT2)Noda>	KI, Reck-CreERT2	[Reck N-terminal 7 codons + CreERT2]<br>expressed under the native *Reck* promoter	Matsuzaki	2018	29508517	Not assigned

Table S7. Hyper-methylation of *RECK* in cancer genomes

Site (or origin of cell line)	Reference		
	First author	Year	PMID
Lung	Chang	2006	16951151
	Chang	2007	17233834
	Pesta	2009	20032402
Breast	Hill	2011	21363912
	Li	2015	25636590
	Shi	2016	27058625
Mouth	Long	2008	18485791
	Zhou	2015	25517920
Esophagus	Zhu	2017	28454343
Stomach	Du	2010	20143471
Liver	Zhang	2012	22419890
	Abo El-Khair	2021	34392581
Pancreas	Lu	2013	23749490
Colon	Cho	2007	17443689

Table S8. Primers for detecting *RECK* gene methylation

Reference (PMID)			Method	Species	Target*	Primers	
First author	Year	PMID					
Chang	2006	16951151	methylation-specific PCR (MSP)	Mouse	promoter	Methylated:	
						Forward	5’-GTTTTTGATTTTTCGTTTGAAGATC-3’
						Reverse	5’-CTCTAA- TAATTAACTACGACTCGCT-3’
						Unmethylated:	
						Forward	5’-TTTTGATTTTTTGTTTGAAGATTGT- 3
						Reverse	5’-TCCTAATAATTAACTACAACTCACT-3’
				Human	promoter	Methylated:	
						Forward	5’-AATAAAGAGTTTTGGTACGGGGTAC-3’
						Reverse	5’-AAAACCGCGAAATACTCGAA-3
						Unmethylated:	
						Forward	5’-TAAAGAGTTTTGGTATGGGGTATGT-3’
						Reverse	5’-CTCCAAACCACAAAATACTCAAA-3’
Long	2008	18485791	methylation-specific PCR (MSP)	Human	promoter	Methylated:	
						Forward	5’-GTTAGTTTTTTTTTTTATTTTAGTGGTTCGA-3’
						Reverse	5’-TCCAAAACCTCCCGAAAACGAAAACG-3’
						Unmethylated:	
						Forward	5’-GGTTAGTTTTTTTTTTTATTTTAGTGGTTTGA-3’
						Reverse	5’-ATTTCCAAAACCTCCCAAAAACAAAAACA-3’
Hill	2011	21363912	combined bisulfite restriction analysis (CoBRA)<br>bisulfite sequencing (2-step PCR)	Human	Intron-1	Forward-1	5’-ATTTTGTTTAYGTTYGGYGATTTYGGGATT-3’
						Forward-2	5’-TATTTATYGATAYGGGTTTTTTTTYGGTATTGATT-3’
						Reverse	5’-ATCCCRCCCCCRAAAAACAAAATTACTA-3’
Li	2015	25636590	bisulfite sequencing	Human	promoter	Forward	5’-TTTAGTGATGAATTTTTGTTAGGGG-3’
						Reverse	5’-CCAAAAAAACTTCTCTCCTTCATATAC-3’
Shi	2016	27058625	combined bisulfite restriction analysis (CoBRA)<br>bisulfite sequencing (2-step PCR)	Human	promoter	Forward-1	5’-ATTTTTTGATTTTATTTTGGGAGAA-3’
						Forward-2	5’-TGGGTTATAATAAAGAGTTTTGGTA-3’
						Reverse	5’-TTACTCTAAAAATTACTCACCC-3’

* See Figure 11 for the positions of targert sites in human *RECK* gene.

Table S9. LncRNAs affecting RECK expression

LncRNA	Target(s)	Reference		
		First author	Year	PMID
Artificial i-lncRNA	miR-17, 19, 20, 21, 125, 155, 146, 221/222	Su	2016	27172795
NBAT1	miR-21	Yang	2017	29119050
GAS5	miR-135b	Yang	2019	30733959
	miR-21	Lin	2020	31866421
SNHG8	EZH2	Qu	2020	31961005
MAGI2-AS3	miR-25	Sui	2020	32138716
LINC01419	EZH2	Zhang	2020	32522890
NEAT1	miR-195-5p	Li	2021	34306328
XIST	miR-195-5p	Li	2021	34306328
MEG8	miR-195-5p	Xu	2021	34790697
GATA2-AS1	miR-21-5p	He	2024	38495494

Table S10. Physical stimuli and environmental conditions affecting RECK expression

Category	System / condition	Effects on RECK*	Reference		
			First author	Year	PMID
**1.** Visible light	Human skin / nonablative laser	U	Oh	2007	17650176
	Human cancer cell line / photodynamic therapy	U	Meng	2022	35260928
**2.** Ionizing radiation	Human cancer cell line / gamma ray	U	Kim	2008	18319621
	Human cancer cell line / X-ray	D	Lin	2020	31866421
**3.** Cell density	Mouse fibroblasts / increasing cell density	U	Hatta	2009	19720143
**4.** Hypoxia	Human epithelial cell lines / low ambient O_2	D	Lee<br>Jeon<br>Jeon	2010<br>2010<br>2011	20080132<br>20442303<br>21468925
	Human colon cancer cell line / deferoxamine	D	Loayza-Puch	2010	20154725
	1) Non-malignant human cell lines / $CoCl_2$<br>2) Human cancer cell lines / $CoCl_2$	1) U<br>2) D	Zhang	2017<br>2018	28561419<br>30116394
	Human cancer cell lines / extracellular vesicles derived from human mesenchymal stem cells under low ambient O_2	D	Ren	2019	30736829
	Rat liver / transient ischemia (*in vivo*)	D	Ferrigno	2020	32403397
	Mouse abdominal aorta / chronic intermittent hypoxia (in vivo)	D	Sharma	2024	38867666
**5.** Magnetic stimulation	Rat vascular smooth muscle cells / repetitive transcranial magnetic stimulation	U	Zhang	2024	39229501

*U: upregulation; D: downregulation.

Table S11. Microbial infection affecting RECK expression

Category	System / gene responsible	Effects on RECK*	Reference		
			First author	Year	PMID
Epstein-Barr virus	human cancer cell line / LMP1	D	Liu	2003	14614450
canine distemper virus	dog monocytic cell line	U	Puff	2009	18684651
Newcastle disease virus	human cancer cell line	U	Zhang	2015	25877754
Mycoplasma pneumoniae	human bronchial epithelial cell line	D	Qin	2022	35892136
human papilloma virus 16	human foreskin keratinocytes / E6, E7	D	Cardeal	2012	22438955

*U: upregulation; D: downregulation.

Table S12. Effects of chemical carninogenesis on RECK expression

Carcinogen	System (method) / tissue examined	Effects on RECK	Reference		
			First author	Year	PMID
dimethylaminoazobenzene	Rat (mixed with diet) / liver and lung	D	Murugan	2009	19528495
N-methyl-N'-nitro-N-nitrosoguanidine	Rat (mixed with diet) / stomach	D	Manikandan	2010	20434464
7,12-dimethylbenz[a]anthracene	Hamster buccal pouch (painting) / buccal pouch	D	Kowshik	2017	28515436
asbestos	Mouse (intratracheal instillation) / lung	D	Hiraku	2021	34679210

Table S13. Expression of RECK in miscellaneous clinical conditions

Area	Condition	RECK expression (sample)	References		
			First author	Year	PMID
Orthopedics	arthritis	1) Rheumatoid arthritis: lower (synovial membrane)<br>2) No change (bovine nasal cartilage incubated with IL1 and OSM *in vitro*)<br>3) Osteoarthritis: higher (chondrocytes)	1) van Lent<br>2) Milner<br>3) Kimura	2005<br>2006<br>2010	15485996<br>16919164<br>20395433
Obstetrics and gynecology	endometriosis	1) No change (peritoneal macrophage)<br>2) Higher (ectopic endometriotic lesions)	1) Wu<br>2) Gozdz	2005<br>2024	16192641<br>39519143
	preterm delivery (chorioamnionitis)	Lower (placental and fetal membranes)	Benzon	2014<br>2018	24227542<br>30230382
	genital *Chlamydia* infection	Lower (cervical tissue)	Discacciati	2015	26261088
	preeclampsia	Higher (trophoblasts)	Gutiérrez	2017	29108633
Thoracic	asthma	1) Lower (sputum)<br>2) Lower in eosinophilic asthma (bronchial brush biopsy)	1) Paulissen<br>2) Kozlik-Siwiec	2006<br>2023	17088949<br>36835202
	pulmonary sarcoidosis	Lower (bronchoalveolar cells)	Navratilova	2016	27575817
	chronic obstructive pulmonary disease (COPD)	Lower (sputum and plasma)	Wang	2024	38387446
Neurology	cocaine abuse	Higher (postomortem hippocampal tisseue)	Mash	2007	18000554
	Alzheimer’s disease	Higher (prefrontal cortex)	Nakamura	2021	33731436
Cardiovascular	atrial fibrillation	Higher (right atrial free wall)	Polyakova	2008	18194448
	ventricular fibrosis in aortic stenosis patients	Higher (myocardial biopsy)	Villar	2013	22882958
	hemorrhagic transformation following intravenous thrombolysis in ischemic stroke	No association with RECK SNPs but significant association with some WNT7A and GPR124 SNPs	Ta	2021	32991049
	acute myocardial infarction	Lower (peripheral blood mononuclear cells)	Vancheri	2021	34205376
Digestive	metabolic dysfunction-associated steatotic liver disease (MASLD)	Lower (liver)	Dashek	2024	38797477
Oral	alveolar bone regeneration	Higher (osteoblasts)	Accorsi-Mendonça	2008	17987394
	periodontal diseases	Lower (gingival epithelium) in chronic periodontitis patients	Liu	2017	28043014
Autoimmunity	systemic lupus erythematosus	Lower (periferal blood monocytes) in systemic lupus erythematosus patients	Hou	2008	18652766
	lupus nephritis	Lower (peripheral blood monocytes; speculated from experimental data with glomerular mesangial cells)	Tomita	2025	40045202
Dermatology	hidradenitis suppurativa	Higher (apocrin glands) in hidradenitis suppurativa lesions	Zouboulis	2020	32031713

Table S14. RECK expression in disease models

Disease or condition	System (method*) / tissue examined // finding	Effects on RECK**	Reference		
			First author	Year	PMID
muscular dystrophy	dog ($CXMD_J$, a dystrophin mutant) / muscle fiber	U	Fukushima	2007	17598883
gingivitis	rat (ligature) / gingival tissue	NC	Lorencini	2009	19085832
microgravity	mouse (hind limb unloading) / tibiae	D	Visigalli	2010	20602768
type-1-diabetes-associated periodontal disease	rat (streptozotocin + ligature) / gingival tissue	D	Silva	2012	21826658
cardiac fibrosis	mouse (Angiotensin-II) / heart	D	Siddesha	2013	24095877
aneurysm	mouse (Angiotensin-II) / abdominal aorta	D	Kim<br>Sharma	2014<br>2024	24812324<br>38867666
traumatic brain injury	mouse (surgery) / damaged brain tissue [age-dependent]	U	Sandhir	2014	25277076
osteoporosis	mouse (bilateral oophorectomy) / mesenchymal stem cell	U	Zhao	2015	25893734
blood vessel injury	rabbit (balloon injury) / carotid artery	U	Liu	2015	26004948
atherosclerosis	mouse (*Apoe* KO) / inflamed endothlium<br>[possible involvement of miR-712 (1), PHATR-1 (2), Traf3ip2 (3), IL-32a (4), and PDGF (5)]	D	1) Kheirolomoom<br>2) Jarray<br>3) Sakamuri<br>4) Son<br>5) Alshanwani	2015<br>2015<br>2016<br>2017<br>2018	26308181<br>26362351<br>27237075<br>28740544<br>29659130
renal fibrosis	rat (cyclosporin-A + furosemide) / kidney	U	Cheng	2018	29462885
transient cerebral ischemia	rat (middle cerebral artery occlusion) / brain tissue [enhanced by tredmill exercise]	D	Tang	2018	30035384
diabetic kidney disease	mouse (*db/db*, a *Lepr* mutant) / kidney<br>[restored by Empagliflozin]	D	Aroor<br>Das	2018<br>2020	30060748<br>31862399
epilepsy	rat (LiCl + atropine + pilocarpine) / hippocampal neuron	D	Du	2019	31018114
Parkinson's disease	rat (rotenone into substantia nigra) / several brain areas	V	Spindola	2020	31493243
hepatic ischemia	rat (microvascular clips) / liver	D	Ferrigno	2020<br>2020	32403397<br>32911524
chronic obstructive pulmonary disease (COPD)	rat (cigarette smoke) / lung airway epithelial cell	D	Li	2020	33155231
Alzheimer’s disease	mouse (*Gde2* KO) / neuron	U***	Nakamura	2021	33731436
alcohol dependence	rat (ethanol vapor) / central nucleus of amygdala	U	Kisby	2021	34573170
colitis	mouse (dextran sodium sulgate in drinking water) / colon	D	Zhang	2022	34962394
peripheral nerve injury	mouse (chronic constriction injury) / sciatic nerve	D	Reinhold	2022	35716075
metabolic dysfunction-associated fatty liver disease (MAFLD)	rat (methionine and choline-deficient diet) / liver	U	Palladini	2022	35743260
type 2 diabetes-associated peripheral neuropathy	rat (streptozotocin) / sciatic nerve [restored by Empagliflozin]	D	Abdelkader	2022	35767208
peripheral nerve injury	mouse (*Dap12* KO) / sciatic nerve	D	Liu	2023	36503041
obesity	mouse (*ob/ob* + high fat diet) / liver	D	Di Pasqua	2023	37904642
obesity, metabolic dysfunction-associated steatohepatitis (MASH)	1) mouse (high fat/sugar/cholesterol diet) / liver<br>2) mouse (*Reck* OE in hepatocytes; fed Western diet) / liver // decreased inflammation and fibrosis	1) D<br>2) U	Dashek	2024	38797477
	mouse (*Reck* cKO in hepatocytes; fed control diet) / liver // increased inflammation and fibrosis	D	Dashek	2025	40758574

*KO: knockout; OE: overexpression; cKO: conditional knockout
**D: downregulation; U: upregulation; V: variable; NC: no change
***increase of membrane-tethered RECK
